# Trends of Obesity in Early Adulthood and Later Adulthood Across 196 Countries and Territories, 1990–2022

**DOI:** 10.1002/oby.70290

**Published:** 2026-08-23

**Authors:** Abdiwahab M. Ali, Edward Giovannucci, Jason J. Liu

**Affiliations:** ^1^ International Health Program National Yang Ming Chiao Tung University Taipei Taiwan; ^2^ Department of Nutrition and Epidemiology Harvard T.H. Chan School of Public Health Boston Massachusetts USA; ^3^ Institute of Public Health National Yang Ming Chiao Tung University Taipei Taiwan

**Keywords:** early adulthood, later adulthood, obesity, trend analysis

## Abstract

**Objective:**

We aimed to understand the global burden of obesity in early adulthood and later adulthood over the past three decades.

**Methods:**

We analyzed obesity prevalence across 196 countries/territories from 1990 to 2022 for early adulthood (age 20–49 years) and later adulthood (age ≥ 50 years) using the Non‐Communicable Disease Risk Factor Collaboration dataset. We calculated the magnitude of change between baseline (1990–1994) and endline (2018–2022) periods and estimated the average annual percent change using segmented regression.

**Results:**

Globally, the magnitude of prevalence increase was higher for obesity in later adulthood than in early adulthood in 134 countries/territories. However, obesity prevalence increased faster in early adulthood than in later adulthood in 168 countries/territories. Women and countries/territories with higher income and baseline obesity prevalence had larger increases in prevalence magnitude, whereas the rate of increase was more rapid in men and countries/territories with lower income and baseline prevalence.

**Conclusions:**

Our findings support prevention strategies to slow the rate of increase in early adulthood obesity prevalence alongside nationwide measures to combat obesity at all ages. Further research is needed to understand age‐specific factors promoting obesity in early and later adulthood.

## Study Importance

- What is already known?
  ○ Obesity in early adulthood is associated with early onset chronic diseases and higher lifetime disease risk.
  ○ Previous global studies have not compared the prevalence and trends of obesity in early adulthood (age 20–49 years) and later adulthood (age ≥ 50 years).
- What does this study add?
  ○ Obesity in early adulthood and lower‐income regions had a faster rate of prevalence increase.
  ○ Obesity in later adulthood and higher‐income regions had a larger magnitude of prevalence increase.
- How might these results change the direction of research or the focus of clinical practice?
  ○ Our findings support prevention strategies to slow the rate of increase in early adulthood obesity prevalence, alongside nationwide measures to combat obesity at all ages.
  ○ Future research on age‐specific factors promoting obesity in early adulthood and later adulthood is needed to better understand obesogenic factors disproportionately affecting younger or older adults.

## 1 Introduction

More than 1 billion people worldwide now live with obesity, and the global age‐standardized obesity prevalence has more than doubled since 1990 [1]. Obesity has been a major global health issue in the past few decades and remains a leading contributor of non‐communicable disease burden [2]. The rise in obesity has occurred in almost every global region for both sexes [1]. Previous global studies of obesity have found substantial increases in prevalence across most countries and regions over the last several decades [1, 3, 4]. However, these studies have only focused on obesity trends for all adults combined, without understanding if these trends differed for those at different stages of adulthood.

Understanding early adulthood obesity is crucial because weight gained at this stage of life often persists into later adulthood [5], which contributes to greater cumulative lifetime exposure to hyperglycemia, dyslipidemia, systemic inflammation, and insulin resistance [6]. Obesity in early adulthood also increases the risk of early onset gastrointestinal cancers (commonly defined as gastrointestinal cancers diagnosed before 50 years of age), particularly colorectal cancer, pancreatic cancer, liver cancer, and esophageal adenocarcinoma [7, 8]. Furthermore, higher adiposity in early adulthood increases the lifetime risk of type 2 diabetes, cardiovascular disease, and metabolic disorders [9, 10].

In this study, we aimed to globally assess the magnitude and rate of change in early adulthood (age 20–49 years) and later adulthood (age ≥ 50 years) obesity prevalence from 1990 to 2022 to fill an important gap in prior studies of global obesity burden aggregating all adults. We examined both the magnitude of change to quantify the absolute change in obesity prevalence over time and the rate of change to identify places with potentially rapidly rising obesity prevalence in the future. Our age‐stratified findings can inform age‐specific obesity prevention and intervention strategies.

## 2 Methods

We analyzed the Non‐Communicable Disease Risk Factor Collaboration (NCD‐RisC) global database, which pooled data from over 3600 cross‐sectional population‐based studies involving 222 million participants from 200 countries/territories. NCD‐RisC produced body mass index (BMI) and obesity prevalence estimates from 1990 to 2022 for all countries/territories by synthesizing available national and subnational data with Bayesian hierarchical modeling. The data collection procedures and statistical methods for generating these estimates have been previously described in detail [11]. Obesity was defined using the standard World Health Organization (WHO) definition of BMI ≥ 30 kg/m^2^ [2]. We conducted a sensitivity analysis applying a lower cut point (BMI 25 kg/m^2^) for South‐East Asian and specific Western Pacific countries. National obesity prevalence estimates for early adulthood (age 20–49 years) and later adulthood (age ≥ 50 years) were calculated by combining annual prevalence estimates for 5‐year age groups, weighted using age‐ and sex‐specific population denominators from the United Nations World Population Prospects 2024 edition [12]. We chose the age cut point of 50 years because it is commonly used to define early onset cancers and is when cardiometabolic disease risk accelerates [13, 14].

To understand the change in the magnitude of global obesity prevalence over time, we calculated the difference in the 5‐year average obesity prevalence at the baseline (1990–1994) and endline (2018–2022) periods for each country/territory. These national estimates were summarized as medians and interquartile ranges (IQR) across WHO regions, income groups, and Human Development Index (HDI) categories. To determine the overall magnitude of change, we subtracted the baseline 5‐year average prevalence from the endline 5‐year average prevalence for each country/territory.

Segmented regression was used to obtain the rate of obesity prevalence change from 1990 to 2022 quantified by the average annual percent change (AAPC) level [15]. Calendar year was the independent variable and the natural log of the population‐weighted obesity prevalence was the dependent variable, which allowed the calculation of AAPC using the formula AAPC = (exp(∑*w*_*i*_ *b*_*i*_/∑*w*_*i*_) ‐ 1) × 100, where *b*_*i*_ is the regression coefficient (i.e., slope of the fitted trend line) for the *i*‐th segment, and *w*_*i*_ is the weight for the *i*‐th segment that represents the proportion of the overall time interval from 1990 to 2022 covered by that segment. Segmented regression provides the same slope that linear regression estimates if a single linear trend line (i.e., one segment) can best fit the obesity prevalence data points during our study period. However, for data points with non‐linear patterns, segmented regression provides a better fit because it has the capability to model multiple linear segments with different slopes connected at breakpoints (joinpoints). The segmented regression model we used allowed segment lengths of at least five calendar years and up to three breakpoints. Models with different numbers of breakpoints were compared for a given analysis, and the number of breakpoints resulting in the minimum Bayesian information criterion value was selected as the optimal one. Of the 784 examined trend lines (from a combination of 196 countries/territories, two sexes, and two age groups), the model selected zero breakpoints for 6 trend lines (0.8%), one breakpoint for 160 trend lines (20.4%), two breakpoints for 308 trend lines (39.3%), and three breakpoints for 310 trend lines (39.5%).

We compared changes in the magnitude and rate of obesity prevalence by baseline prevalence, WHO region, World Bank income group, and HDI. Baseline 5‐year average obesity prevalence from 1990 to 1994 was categorized into tertiles. The ranges for low (bottom 33%), middle (middle 33%), and high (top 33%) tertiles of averaged 1990–1994 baseline prevalence were: < 6.15%, 6.15%–12.82%, > 12.82% (for women age 20–49); < 12.65%, 12.65%–25.03%, > 25.03% (for women age ≥ 50); < 2.13%, 2.13%–9.04%, > 9.04% (for men age 20–49); and < 3.26%, 3.26%–14.46%, > 14.46% (for men age ≥ 50). WHO region (African Region, Region of the Americas, South‐East Asia Region, European Region, Eastern Mediterranean Region, and Western Pacific Region) was categorized using WHO's official regional classification. National income was categorized using the World Bank's July 1, 2022, classification of gross national income per capita with the Atlas method. The income ranges were (in US dollars): low income (< $1085), lower‐middle income ($1086–$4255), upper‐middle income ($4256–$13,205), and high income (> $13,205) [16]. HDI categories followed the United Nations Development Programme's classification: low (< 0.550), medium (0.550–0.699), high (0.700–0.799), and very high (≥ 0.800) [17]. State of Palestine, Cook Islands, Niue, and Tokelau were excluded due to their lack of geospatial data for generating map visualization. Venezuela and North Korea were respectively excluded from analyses involving income and HDI because they were unclassified for these variables. Countries and territories were categorized by quartiles of AAPC level for mapping. Analyses were conducted in R (version 4.5.1) using the “segmented” package for regression modeling [15]. This study analyzed publicly available data from the NCD‐RisC database, and no ethical approval was required.

## 3 Results

Globally, the median obesity prevalence was higher in later adulthood than in early adulthood during both the baseline (1990–1994) and endline (2018–2022) periods (Table 1). At the baseline period, the global median prevalence for early adulthood and later adulthood obesity was 9.4% and 19.6% in women and 5.5% and 9.0% in men, respectively. By the endline period, the global median prevalence for early adulthood and later adulthood obesity more than doubled in most demographic groups, reaching 19.7% and 33.7% in women and 17.2% and 22.4% in men, respectively. Across WHO regions, the Americas and Eastern Mediterranean regions consistently exhibited the highest median prevalence at both time periods, whereas the African and South‐East Asian regions had the lowest median prevalence.

**TABLE 1:** Median obesity prevalence at baseline (1990–1994) and endline (2018–2022) by WHO region, World Bank income group, and baseline prevalence level.

Category		*N*	Women, age 20–49	Women, age ≥ 50	Men, age 20–49	Men, age ≥ 50
Baseline prevalence (%) in 1990–1994, median (IQR)						
Global		196	9.4 (4.1–15.0)	19.6 (6.2–27.3)	5.5 (1.3–9.8)	9.0 (2.2–16.0)
WHO region	African	47	3.0 (1.6–6.0)	4.7 (2.5–9.5)	0.9 (0.7–1.3)	1.8 (1.3–2.6)
	Americas	37	13.5 (10.7–18.4)	21.4 (19.2–25.0)	8.3 (5.1–10.7)	8.4 (6.6–14.5)
	Eastern Mediterranean	21	20.4 (5.4–25.2)	26.7 (9.8–34.0)	10.6 (2.3–12.5)	13.4 (3.3–17.2)
	European	52	9.7 (8.1–12.6)	26.1 (20.1–31.3)	8.7 (7.0–9.8)	15.6 (12.6–17.1)
	South‐East Asia	11	2.3 (0.8–3.0)	2.8 (1.4–3.7)	0.7 (0.5–1.2)	0.8 (0.7–1.7)
	Western Pacific	28	14.5 (3.4–39.2)	17.6 (5.7–39.4)	11.0 (2.1–27.7)	12.5 (2.2–30.1)
World Bank income level	Low income	26	1.9 (1.0–2.8)	3.2 (1.6–3.8)	0.8 (0.5–1.1)	1.6 (0.9–1.8)
	Lower‐middle income	50	4.4 (2.6–10.5)	6.7 (4.4–16.6)	1.3 (0.9–5.0)	2.3 (1.5–9.0)
	Upper‐middle income	53	12.4 (9.1–17.3)	21.8 (17.9–30.6)	5.5 (4.2–9.1)	9.1 (5.4–14.6)
	High income	66	12.2 (8.8–19.0)	25.3 (19.8–31.5)	9.8 (7.9–12.7)	16.3 (12.1–19.0)
Baseline (1990–1994) prevalence	Low	66	2.5 (1.4–4.1)	3.7 (2.4–6.2)	0.9 (0.6–1.3)	1.6 (0.9–2.2)
	Medium	65	9.4 (8.1–11.2)	19.6 (16.7–21.5)	5.6 (4.7–7.6)	9.1 (6.6–11.4)
	High	65	19.1 (15.1–25.2)	31.9 (27.3–38.0)	11.5 (9.8–16.2)	17.4 (16.1–20.7)
Endline prevalence (%) in 2018–2022, median (IQR)						
Global		196	19.7 (12.9–32.8)	33.7 (19.6–44.9)	17.2 (7.0–24.2)	22.4 (9.8–32.1)
WHO region	African	47	13.7 (9.7–19.7)	19.8 (13.3–25.4)	4.7 (3.5–6.7)	8.5 (6.0–11.3)
	Americas	37	34.7 (29.9–41.8)	43.3 (37.5–46.2)	22.5 (19.5–27.7)	23.9 (20.0–31.5)
	Eastern Mediterranean	21	32.7 (22.4–38.9)	49.5 (27.0–63.0)	24.1 (12.2–28.3)	30.8 (16.3–40.0)
	European	52	16.7 (14.2–20.1)	36.9 (27.9–44.0)	19.1 (15.9–22.8)	30.4 (27.8–35.5)
	South‐East Asia	11	12.5 (7.6–15.3)	12.2 (9.7–13.4)	6.1 (4.4–8.0)	5.8 (4.4–6.8)
	Western Pacific	28	26.6 (9.5–54.0)	33.2 (10.9–56.2)	20.9 (12.4–39.0)	26.3 (6.6–38.4)
World Bank income level	Low income	26	10.1 (6.9–13.4)	13.4 (8.4–21.7)	4.2 (3.0–7.6)	7.0 (4.7–10.3)
	Lower‐middle income	50	17.8 (12.2–25.1)	23.9 (15.7–39.5)	6.5 (4.6–15.8)	10.5 (6.7–20.2)
	Upper‐middle income	53	28.1 (20.4–39.4)	42.8 (33.9–48.4)	18.2 (14.3–23.4)	25.1 (17.7–31.2)
	High income	66	21.3 (15.1–38.4)	38.2 (26.7–45.8)	23.3 (18.4–30.6)	31.4 (24.4–39.0)
Baseline (1990–1994) prevalence	Low	66	11.0 (7.8–15.4)	14.4 (10.2–21.8)	4.9 (3.6–7.0)	6.9 (4.8–9.8)
	Medium	65	20.4 (15.5–27.8)	36.3 (28.5–42.8)	17.9 (15.4–20.9)	22.7 (19.4–28.2)
	High	65	38.7 (27.9–45.8)	46.4 (42.4–57.4)	27.3 (22.4–35.8)	36.1 (31.1–40.4)

*Note:* *N*: number of countries/territories. IQR consists of quartile 1–quartile 3 values. Prevalence estimates are 5‐year averages. World Bank income was categorized using 2022 fiscal year data, with Venezuela unclassified and excluded from the analysis.

Table 2 provides the changes in the magnitude and rate of obesity prevalence from 1990 to 2022. The country/territory‐specific results are presented in Table S1. Globally, the median increase in prevalence magnitude was larger for later adulthood obesity than early adulthood obesity in both sexes (women: 14.5% vs. 10.8%; men: 11.8% vs. 10.1%). Regionally, the largest median prevalence change for early adulthood obesity occurred in the Americas, while the Eastern Mediterranean region had the largest increase for later adulthood obesity. Across World Bank income categories, prevalence change was greatest in upper‐middle‐income settings for early and later adulthood women, while upper‐middle‐income and high‐income settings had the largest prevalence change for men in both age groups. The HDI‐specific results were broadly consistent (Table S2). When examined by baseline obesity prevalence, countries/territories in the high‐baseline and middle‐baseline tertiles experienced larger changes in prevalence magnitude than those in the low‐baseline tertile, especially for men.

**TABLE 2:** Magnitude and rate of obesity prevalence change from 1990–2022 by WHO region, World Bank income group, and baseline prevalence level.

		*N*	Women, age 20–49	Women, age ≥ 50	Men, age 20–49	Men, age ≥ 50
Prevalence change (%) from 1990–1994 to 2018–2022, median (IQR)						
Global		196	10.8 (6.4–17.2)	14.5 (7.2–20.6)	10.1 (5.2–14.4)	11.8 (6.8–16.8)
WHO region	African	47	10.4 (7.6–14.6)	14.1 (9.0–17.4)	3.7 (2.8–5.3)	6.4 (4.5–8.9)
	Americas	37	21.8 (19.2–24.9)	21.9 (17.6–22.7)	15.8 (12.9–18.0)	14.4 (12.5–17.8)
	Eastern Mediterranean	21	12.9 (10.5–16.5)	21.9 (18.6–26.2)	13.5 (10.5–15.2)	17.4 (11.4–20.8)
	European	52	5.5 (4.1–9.1)	6.3 (1.8–13.3)	10.8 (8.2–12.9)	15.5 (12.0–19.1)
	South‐East Asia	11	9.5 (6.6–12.6)	8.3 (7.4–10.1)	5.5 (3.7–6.9)	4.9 (3.7–5.2)
	Western Pacific	28	10.8 (6.2–15.2)	11.5 (4.8–15.9)	11.0 (6.2–14.2)	8.2 (4.6–14.3)
World Bank income level	Low income	26	7.9 (5.8–11.2)	10.2 (6.7–17.0)	3.4 (2.7–6.3)	5.5 (4.0–8.5)
	Lower‐middle income	50	10.8 (8.4–14.7)	14.8 (10.5–20.7)	5.1 (3.4–8.3)	7.6 (5.1–11.9)
	Upper‐middle income	53	16.5 (11.4–20.4)	18.1 (14.7–22.6)	12.6 (9.4–14.8)	14.4 (11.9–18.2)
	High income	66	8.8 (4.6–15.0)	8.6 (2.0–19.2)	12.4 (9.7–16.9)	14.5 (10.5–18.8)
Baseline (1990–1994) prevalence	Low	66	8.4 (5.8–12.6)	10.8 (7.1–15.8)	4.1 (2.9–5.8)	5.4 (4.0–7.3)
	Medium	65	10.4 (5.4–18.7)	17.4 (8.3–22.7)	12.3 (9.0–14.6)	14.4 (11.4–17.2)
	High	65	14.9 (10.3–23.1)	16.4 (6.8–21.9)	13.5 (11.2–17.7)	15.6 (12.6–20.3)
AAPC 1990–2022, median (IQR)						
Global		196	3.2 (1.8–4.8)	2.4 (1.2–4.3)	4.2 (2.9–6.0)	3.7 (2.6–5.3)
WHO region	African	47	5.6 (4.3–6.5)	5.0 (3.7–5.6)	6.1 (5.1–6.5)	5.4 (4.9–6.1)
	Americas	37	3.4 (3.2–4.0)	2.4 (2.1–2.8)	4.1 (3.6–4.9)	3.7 (2.7–4.0)
	Eastern Mediterranean	21	2.1 (1.7–4.0)	2.4 (2.2–3.6)	3.1 (2.9–4.7)	3.2 (2.8–4.8)
	European	52	1.7 (1.2–2.4)	0.7 (0.2–1.8)	3.0 (2.4–3.7)	2.6 (2.0–3.2)
	South‐East Asia	11	6.8 (5.4–7.3)	5.7 (4.1–6.6)	7.9 (7.4–8.4)	5.9 (5.5–6.9)
	Western Pacific	28	1.9 (1.3–3.9)	1.8 (0.9–2.9)	2.8 (1.2–6.5)	2.5 (1.3–4.7)
World Bank income level	Low income	26	6.3 (5.6–6.9)	5.5 (4.8–6.4)	6.3 (5.9–7.2)	5.7 (5.4–6.4)
	Lower‐middle income	50	4.6 (3.1–5.7)	4.4 (2.9–5.1)	5.7 (4.0–6.6)	5.2 (3.7–5.9)
	Upper‐middle income	53	3.2 (1.7–3.9)	2.4 (1.3–2.8)	4.1 (3.4–5.0)	3.7 (2.8–4.3)
	High income	66	2.0 (1.3–2.9)	1.1 (0.3–2.1)	2.9 (2.3–3.8)	2.6 (1.9–3.3)
Baseline (1990–1994) prevalence	Low	66	5.7 (4.5–6.8)	5.1 (4.3–5.9)	6.4 (5.9–7.7)	5.6 (5.2–6.2)
	Medium	65	2.8 (1.7–3.8)	2.4 (1.3–2.8)	4.1 (3.5–4.9)	3.7 (3.0–4.2)
	High	65	2.0 (1.2–2.8)	1.1 (0.7–2.1)	2.8 (1.9–3.1)	2.3 (1.6–2.8)

*Note:* *N*: number of countries/territories. IQR consists of quartile 1–quartile 3 values. World Bank income was categorized using 2022 fiscal year data, with Venezuela unclassified and excluded from the analysis.

For the rate of prevalence change, early adulthood obesity increased faster than later adulthood obesity for both women (AAPC_20–49_ = 3.2%; AAPC_≥ 50_ = 2.4%) and men (AAPC_20–49_ = 4.2%; AAPC_≥ 50_ = 3.7%) (Table 2). Regionally, the fastest increases occurred in the African and South‐East Asian regions, whereas the slowest increases occurred in the European and Western Pacific regions. Across World Bank income categories, low‐income and lower‐middle‐income countries/territories had the fastest increases in obesity prevalence. A similar pattern was observed by HDI categories, with faster increases in countries/territories with low and medium HDI (Table S2). When examined by baseline obesity prevalence, countries/territories with lower baseline prevalence had higher AAPC values.

Table S3 shows the magnitude and rate of change for the prevalence of BMI ≥ 25 kg/m^2^ in the South‐East Asian and Western Pacific regions, which have countries that use lower BMI cut points (25–27.5 kg/m^2^) to define obesity. After applying the lower BMI cut point, we found larger increases in prevalence magnitude and slower rates of increase over time.

To understand the prevalence and trends of obesity in finer age groups of early and later adulthood, we examined those of age 20–29, 30–39, 40–49, 50–59, 60–69, and ≥ 70 years (Table S4). Globally, obesity prevalence at both the baseline and endline periods was highest for those age 40–69. The median magnitude of prevalence change increased across age groups from age 20–29 to 60–69 and then decreased in the age ≥ 70 group. This pattern was consistent by region; however, the Americas, South‐East Asian, and Western Pacific regions also had a high magnitude of prevalence change for those age 30–39, whereas the Eastern Mediterranean and European regions also had a high magnitude of prevalence change for those age ≥ 70. In contrast with the magnitude of prevalence increase over time, the rate of prevalence increase had a U‐shaped pattern across age groups, with the fastest increase in the age 20–29 and ≥ 70 groups, and the slowest increase for those age 40–69. This U‐shaped pattern was also apparent by region and income level.

**FIGURE 1:**
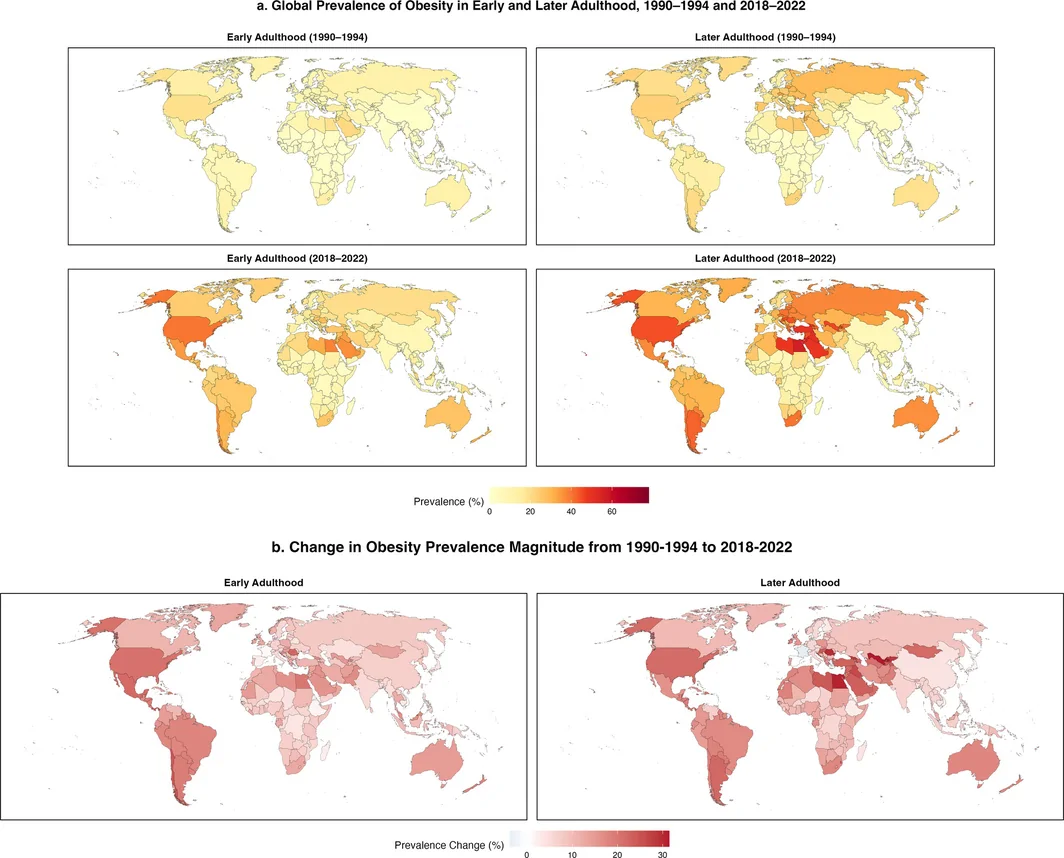
Global obesity prevalence in early adulthood and later adulthood in 1990–1994 and 2018–2022 and the change in prevalence magnitude from 1990–1994 to 2018–2022. (a) Global prevalence of obesity in early adulthood (age 20–49 years) and later adulthood (age ≥ 50 years) in 1990–1994 and 2018–2022. (b) Change in early adulthood and later adulthood obesity prevalence magnitude from 1990–1994 to 2018–2022. Prevalence estimates represent 5‐year averages (1990–1994 vs. 2018–2022) to minimize single‐year fluctuations. [Color figure can be viewed at wileyonlinelibrary.com]

Figure 1 maps the global distributions of averaged obesity prevalence in the baseline (1990–1994) and endline (2018–2022) periods, as well as the change in prevalence magnitude between these periods. At the baseline period, higher obesity prevalence was largely confined to the Americas and parts of the Eastern Mediterranean, while most other regions showed low levels, particularly in early adulthood (Figure 1a). By the endline period, countries/territories with high‐prevalence obesity in early and later adulthood expanded globally, with several places exceeding 40% prevalence for later adulthood obesity (Figure 1a). Figure 1b shows the magnitude of prevalence change between the averaged prevalence at the baseline (1990–1994) and endline (2018–2022) periods. The largest prevalence changes were in the Americas, North Africa, the Eastern Mediterranean, Central Asia, and Eastern Europe. In contrast, Western Europe, East Asia, and Central Africa had smaller prevalence changes (Figure 1b). The sex‐specific maps are provided in Figure S1.

**FIGURE 2:**
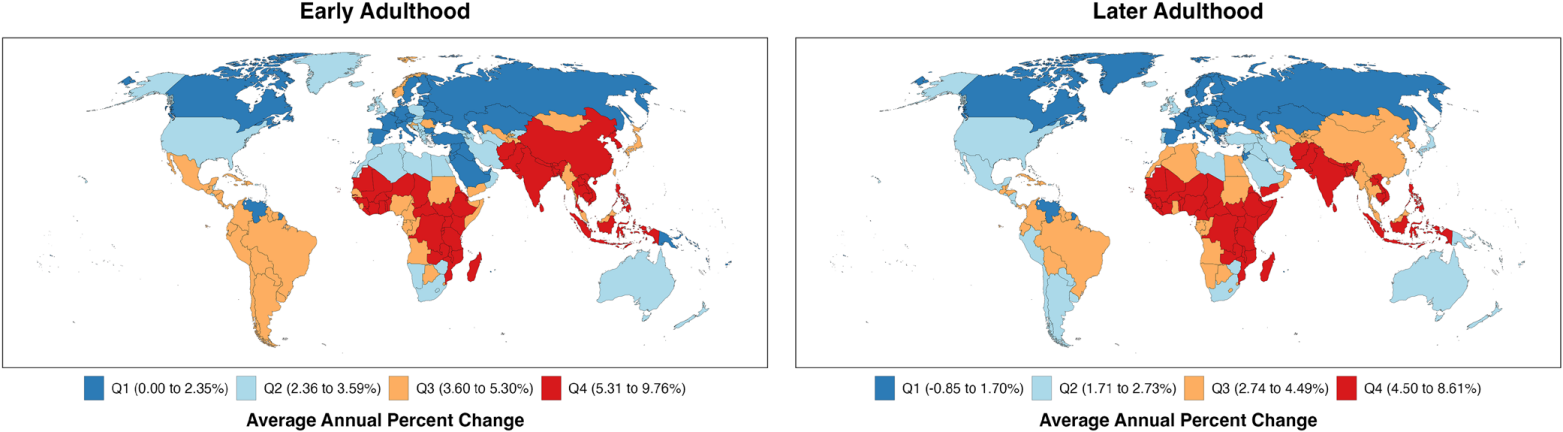
Quartiles of average annual percent change (AAPC) for obesity prevalence in early adulthood and later adulthood from 1990 to 2022. AAPC by quartiles for early adulthood (age 20–49 years) and later adulthood (age ≥ 50 years) obesity. Q4 (red) represents the quartile with the fastest increasing prevalence (highest AAPC values), whereas Q1 (blue) represents the quartile with the slowest increasing prevalence (lowest AAPC values). [Color figure can be viewed at wileyonlinelibrary.com]

Figure 2 maps the rate of change in obesity prevalence from 1990 to 2022 globally. Countries/territories with the highest rate of prevalence increase (AAPC in the fourth quartile) were in South Asia, the Horn of Africa, Eastern and Central Sub‐Saharan Africa, and parts of East and South‐East Asia for both early adulthood and later adulthood obesity. In contrast, Western Europe and parts of North America had the lowest rate of prevalence increase. The sex‐specific maps are presented in Figure S2.

**FIGURE 3:**
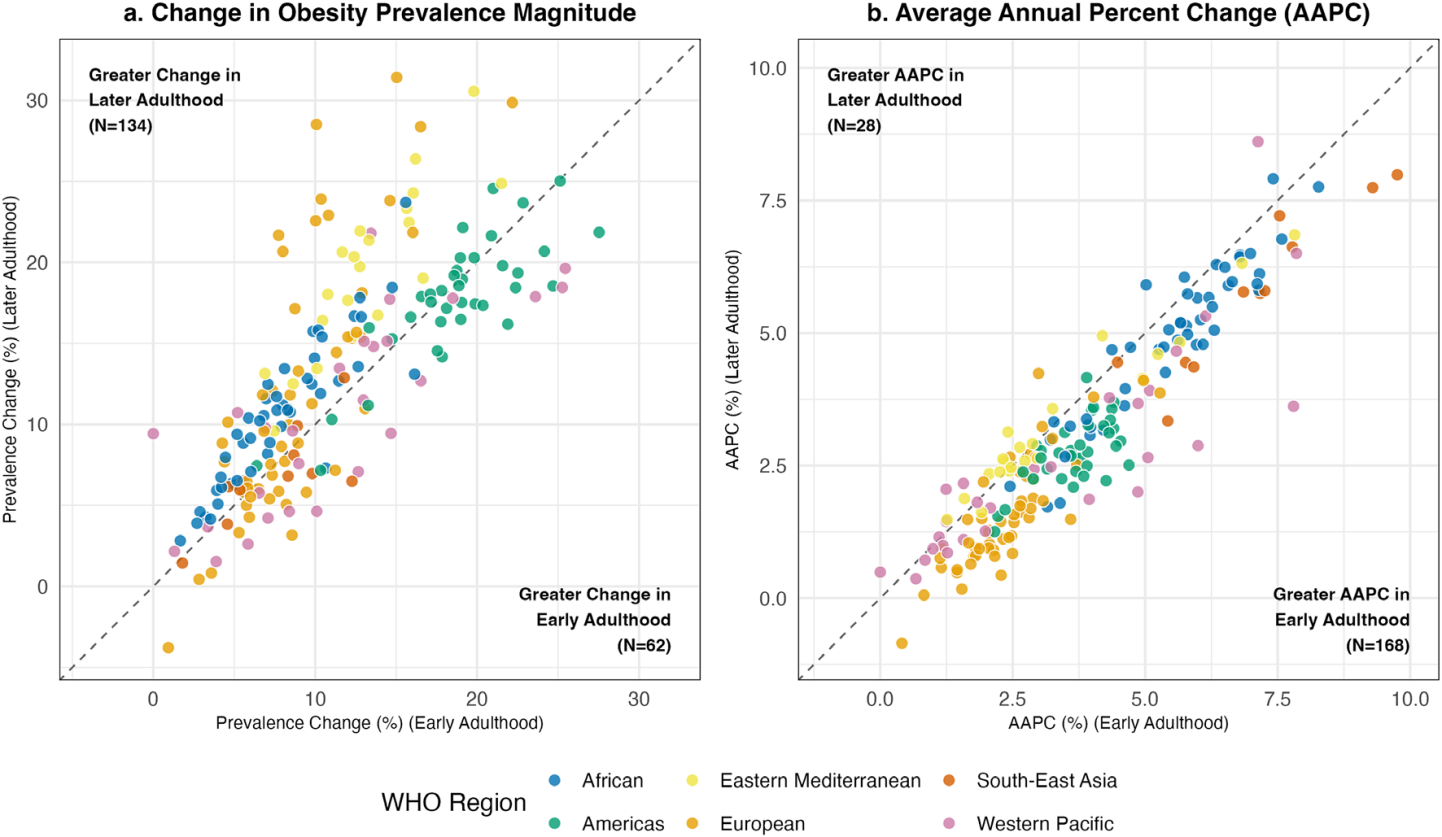
Comparison of changes in the magnitude and rate (average annual percent change [AAPC]) of early adulthood and later adulthood obesity prevalence over time for 196 countries/territories. (a) Change in prevalence magnitude from 1990–1994 to 2018–2022 for early adulthood (age 20–49 years) and later adulthood (age ≥ 50 years) obesity. (b) AAPC from 1990–2022 for early adulthood and later adulthood obesity. *N*: Number of countries/territories. The dashed diagonal line represents equal change in prevalence over time for early adulthood and later adulthood obesity. Points below the diagonal represent countries/territories where the increase in magnitude or rate over time was greater for obesity prevalence in early adulthood than in later adulthood, whereas points above the diagonal indicate the opposite. [Color figure can be viewed at wileyonlinelibrary.com]

Figure 3 compares the magnitude and rate of prevalence change for early adulthood and later adulthood obesity across WHO regions. In Figure 3a, the magnitude of change in prevalence was generally larger for later adulthood obesity (in 134 countries/territories) than early adulthood obesity (in 62 countries/territories). Figure 3b shows that the rate of change was more often higher for early adulthood obesity (in 168 countries/territories) than later adulthood obesity (in 28 countries/territories). The sex‐specific scatterplots are presented in Figure S3.

## 4 Discussion

To our knowledge, this is the first global analysis comparing early adulthood and later adulthood obesity prevalence trends. Between 1990 and 2022, the burden of obesity increased substantially worldwide, with the largest increases in magnitude in later adulthood and higher‐income regions, such as the Americas and Eastern Mediterranean. In contrast, obesity prevalence increased faster in early adulthood and lower‐income settings, particularly in the South‐East Asian and African regions.

The faster rise of early adulthood obesity than later adulthood obesity in many locations globally may be due to the lower baseline obesity prevalence of younger adults and this population's increasing susceptibility to the obesogenic environment. Processed foods high in added sugars, refined carbohydrates, saturated fats, and salt have been shown to contribute a larger share of total energy intake in younger adults than older adults [18, 19, 20], which may be reinforced by the greater exposure of younger adults to digital food advertising that is disproportionately directed toward younger people [21]. Despite the faster increase of early adulthood obesity, older adults have higher current obesity prevalence and substantial exposure to obesity risk factors such as processed foods, which highlights the equal importance of obesity prevention in older adults. Elderly adults are also likely to have higher proportions of harmful visceral and ectopic fat than younger adults given the same BMI, because aging leads to the loss of muscle mass and normal subcutaneous fat [22].

Obesity intervention in early adulthood has important implications. Weight gained in early adulthood increases cumulative exposure to hyperglycemia, dyslipidemia, systemic inflammation, and insulin resistance, raising lifetime risk of type 2 diabetes, cardiovascular disease, and early onset gastrointestinal cancers including colorectal cancer, pancreatic cancer, liver cancer, and esophageal adenocarcinoma [5, 6, 7, 8]. Although obesity prevention and intervention can benefit adults at any age, it can be especially important in early adulthood, when dietary and physical activity routines are being established and weight gain can be relatively rapid [23]. Establishing obesity prevention behaviors in childhood is also crucial for reducing obesity throughout adulthood, because childhood is the most important stage of life for forming health behaviors [24]. Preventing obesity during childhood and early adulthood can be more beneficial for chronic disease prevention by establishing healthier routines early in life before cardiometabolic disease occurs [25, 26].

In our sex‐specific analyses, women generally experienced larger increases in obesity prevalence magnitude than men across most regions and income groups. Sex differences in obesity prevalence can be contributed to by several factors. Social factors can contribute to obesity throughout adulthood, particularly in lower‐resource settings, where gender inequality, lower educational attainment, greater domestic workload, and reduced opportunities for physical activity disproportionately affect women beginning in early adult life [27]. In early adulthood, female obesity is also driven by reproductive factors such as pregnancy‐related weight gain and postpartum weight retention [28, 29]. In later adulthood, menopausal changes in estrogen‐related regulation of appetite, energy expenditure, and fat distribution contribute to increases in central and total adiposity [30, 31].

Countries/territories experiencing the fastest increases in obesity prevalence potentially have high future obesity burden, so obesity prevention strategies should be implemented even if current obesity burden is not high. The fastest increases in obesity prevalence have occurred in low‐income and lower‐middle‐income countries/territories, where possible rapid urbanization and changes in food availability may have contributed to changes in physical activity and dietary patterns [32]. Active commuting has been displaced by motorized transport in some countries/territories, particularly in rapidly urbanizing Asian and African cities, which can translate to reduced daily energy expenditure [33, 34]. Meanwhile, occupational energy expenditure has declined in some countries/territories due to mechanization and moving from physically demanding agricultural work to sedentary service‐sector employment [35, 36]. Furthermore, the increasing intake of processed foods over time has affected countries/territories globally [37, 38]. Although higher‐income countries/territories can also be affected by the obesity‐related factors described earlier, their higher baseline obesity prevalence limited fast rates of obesity prevalence increase relative to the baseline prevalence level.

Countries/territories with the largest increases in obesity prevalence magnitude face a substantial current burden. The obesity burden remains substantial in higher‐income countries/territories. Strategies on reducing obesity and limiting downstream diseases include strengthening clinical and community weight management services, improving access to healthy foods for lower‐income groups, creating physical activity‐friendly built environments, and continuing regulatory measures that reduce intake of processed products high in sugar [39]. Effective interventions to reduce the purchase of unhealthy foods include taxation of sugar‐sweetened beverages, mandatory front‐of‐pack nutrition labeling, and elimination of industrially produced trans fats [40, 41, 42]. Recently, the use of glucagon‐like peptide‐1 (GLP‐1) receptor agonists has been conditionally recommended by WHO for treating obesity in nonpregnant individuals [43]. However, the high cost of GLP‐1 remains a barrier for widespread use, and usage side effects such as gastrointestinal issues require consideration [43]. Even with the availability of GLP‐1, it remains important to eat healthily and exercise regularly, because these behaviors provide health benefits beyond those obtained from lowering BMI [43, 44, 45].

The strength of this study is its global coverage, using both age‐specific and sex‐specific obesity prevalence estimates across 196 countries/territories spanning over more than 30 years. We also provided both the magnitude and rate of change in obesity prevalence to respectively quantify how much and how quickly obesity prevalence has changed globally. Our study also has some limitations. NCD‐RisC estimates come from pooled surveys with heterogeneous measurement protocols and uneven coverage across calendar years, and some countries/territories rely more heavily on statistical modeling because of sparse direct measurements. Another limitation is that the NCD‐RisC data universally applied the WHO definition of obesity to all countries/territories, even though some Asian countries/territories use lower BMI cut points (25–27.5 kg/m^2^) for defining obesity [46]. Although our data did not allow us to apply the 27.5 kg/m^2^ cut point, our sensitivity analysis for the South‐East Asian and Western Pacific regions using the WHO definition of overweight and obesity (≥ 25 kg/m^2^) resulted in larger increases in prevalence magnitude and slower rates of increase over time, compared with using the WHO definition of obesity (≥ 30 kg/m^2^). Because BMI does not directly measure body composition or metabolic health, global studies comparing central obesity and metabolic health in early adulthood and later adulthood are needed.

## 5 Conclusion

Obesity prevalence increased substantially worldwide from 1990 to 2022, with larger‐magnitude increases in later adulthood and higher‐income countries/territories and faster increases in early adulthood and lower‐income countries/territories. Future research can better understand which obesogenic factors disproportionately affect younger or older adults. Furthermore, cohort studies with longitudinal follow‐up data can clarify how changes in obesity status from early adulthood to later adulthood affect chronic disease risk. Our findings support prevention strategies to slow the rate of increase in early adulthood obesity prevalence, alongside nationwide measures to combat obesity at all ages.

## Author Contributions

**Abdiwahab M. Ali:** conceptualization, methodology, formal analysis, writing – original draft, writing – review and editing. **Edward Giovannucci:** writing – review and editing. **Jason J. Liu:** conceptualization, methodology, formal analysis, supervision, writing – original draft, writing – review and editing.

## Funding

The authors have nothing to report.

## Conflicts of Interest

The authors declare no conflicts of interest.

## Supporting information


**Table S1:** Country/territory‐specific obesity prevalence, prevalence change, and average annual percent change (AAPC) from 1990 to 2022.
**Table S2:** Median obesity prevalence at baseline (1990–1994) and endline (2018–2022), prevalence change, and average annual percent change (AAPC) by Human Development Index (HDI).
**Table S3:** Prevalence of BMI ≥ 25 kg/m^2^ and ≥ 30 kg/m^2^ in South‐East Asian and Western Pacific regions.
**Table S4:** Median obesity prevalence at baseline (1990–1994) and endline (2018–2022), prevalence change, and average annual percent change (AAPC) by 10‐year age groups.
**Figure S1:** Global obesity prevalence in early adulthood and later adulthood at 1990–1994 and 2018–2022 and the change in prevalence magnitude from 1990–1994 to 2018–2022, for women and men.
**Figure S2:** Quartiles of average annual percent change (AAPC) for obesity prevalence in early adulthood and later adulthood from 1990 to 2022 among women and men.
**Figure S3:** Comparing changes in the magnitude and rate (average annual percent change [AAPC]) of early adulthood and later adulthood obesity prevalence over time across 196 countries and territories for women and men.

## Data Availability

This study analyzed publicly available data from the Non‐Communicable Disease Risk Factor Collaboration (NCD‐RisC) database. The country‐specific obesity prevalence estimates are freely available from the NCD‐RisC data download page (https://ncdrisc.org/data‐downloads‐adiposity.html).
