# Supplementary material for: Trends of Obesity in Early Adulthood and Later Adulthood Across 196 Countries and Territories, 1990–2022
Source: Obesity (Silver Spring). 2026 Aug 23;34(10):1979–88. doi: 10.1002/oby.70290 (PMC13615427; doi:10.1002/oby.70290)
Supplement: Supplementary file 1 [file OBY-34-1979-s001.docx]

**Trends of Obesity in Early Adulthood and Later Adulthood across 196 Countries and Territories, 1990–2022**

Abdiwahab M. Ali^1^, Edward Giovannucci^2^, Jason J. Liu^1,3^

^1^International Health Program, National Yang Ming Chiao Tung University, Taipei, Taiwan

^2^Department of Nutrition and Epidemiology, Harvard TH Chan School of Public Health, Boston, USA.

^3^Institute of Public Health, National Yang Ming Chiao Tung University, Taipei, Taiwan

**Corresponding author:**

Jason J. Liu, ScD, Institute of Public Health, National Yang Ming Chiao Tung University, No. 155, Sec. 2, Linong St., Beitou District, Taipei City 112, Taiwan. Email: [jasonjliu.jjl@gmail.com](mailto:jasonjliu.jjl@gmail.com)

**Supplementary Table S1. Country/territory-specific obesity prevalence, prevalence change, and average annual percent change (AAPC) from 1990 to 2022**

| **Country/Territory** | **Sex** | **Age Group** | **Baseline (1990-1994) Prevalence (%)** | **Endline (2018-2022) Prevalence (%)** | **Prevalence Change (%)** | **Number of Joinpoints** | **AAPC (95% CI)** | **WHO Region** | **World Bank Income** | **HDI** |
| --- | --- | --- | --- | --- | --- | --- | --- | --- | --- | --- |
| Afghanistan | Men | Early Adulthood | 1.69 | 12.21 | 10.52 | 3 | 7.28 (7.25 to 7.30) | Eastern Mediterranean | Low income | Low |
| Afghanistan | Men | Later Adulthood | 2.71 | 14.59 | 11.88 | 2 | 6.19 (6.17 to 6.20) | Eastern Mediterranean | Low income | Low |
| Afghanistan | Women | Early Adulthood | 2.08 | 19.37 | 17.29 | 3 | 8.24 (8.22 to 8.25) | Eastern Mediterranean | Low income | Low |
| Afghanistan | Women | Later Adulthood | 3.38 | 24.15 | 20.77 | 3 | 7.22 (7.20 to 7.23) | Eastern Mediterranean | Low income | Low |
| Albania | Men | Early Adulthood | 5.83 | 15.62 | 9.79 | 2 | 3.67 (3.63 to 3.71) | European | Upper middle income | Very High |
| Albania | Men | Later Adulthood | 12.22 | 29.43 | 17.2 | 3 | 3.15 (3.15 to 3.16) | European | Upper middle income | Very High |
| Albania | Women | Early Adulthood | 8.44 | 16.11 | 7.67 | 2 | 2.26 (2.22 to 2.29) | European | Upper middle income | Very High |
| Albania | Women | Later Adulthood | 26.28 | 43.33 | 17.05 | 2 | 1.77 (1.76 to 1.78) | European | Upper middle income | Very High |
| Algeria | Men | Early Adulthood | 3.94 | 14.29 | 10.34 | 3 | 4.68 (4.67 to 4.69) | African | Upper middle income | High |
| Algeria | Men | Later Adulthood | 5.39 | 17.65 | 12.26 | 3 | 4.27 (4.25 to 4.29) | African | Upper middle income | High |
| Algeria | Women | Early Adulthood | 14.13 | 28.51 | 14.38 | 2 | 2.55 (2.54 to 2.56) | African | Upper middle income | High |
| Algeria | Women | Later Adulthood | 20 | 41.43 | 21.42 | 3 | 2.61 (2.60 to 2.62) | African | Upper middle income | High |
| American Samoa | Men | Early Adulthood | 60.73 | 72.15 | 11.42 | 2 | 0.66 (0.64 to 0.67) | Western Pacific | High income | Very High |
| American Samoa | Men | Later Adulthood | 61.7 | 69.01 | 7.31 | 2 | 0.43 (0.42 to 0.44) | Western Pacific | High income | Very High |
| American Samoa | Women | Early Adulthood | 68.49 | 82.37 | 13.88 | 3 | 0.69 (0.68 to 0.70) | Western Pacific | High income | Very High |
| American Samoa | Women | Later Adulthood | 73.23 | 79.5 | 6.27 | 3 | 0.29 (0.29 to 0.29) | Western Pacific | High income | Very High |
| Andorra | Men | Early Adulthood | 8.28 | 18.3 | 10.02 | 2 | 2.88 (2.84 to 2.92) | European | High income | Very High |
| Andorra | Men | Later Adulthood | 14.75 | 27.31 | 12.56 | 1 | 2.22 (2.20 to 2.25) | European | High income | Very High |
| Andorra | Women | Early Adulthood | 8.1 | 12.65 | 4.56 | 2 | 1.61 (1.55 to 1.66) | European | High income | Very High |
| Andorra | Women | Later Adulthood | 23.74 | 24.56 | 0.82 | 1 | 0.13 (0.12 to 0.14) | European | High income | Very High |
| Angola | Men | Early Adulthood | 1.28 | 4.68 | 3.4 | 3 | 4.85 (4.83 to 4.87) | African | Lower middle income | Medium |
| Angola | Men | Later Adulthood | 2.48 | 8.07 | 5.58 | 2 | 4.31 (4.27 to 4.35) | African | Lower middle income | Medium |
| Angola | Women | Early Adulthood | 4.32 | 14.93 | 10.61 | 2 | 4.55 (4.54 to 4.57) | African | Lower middle income | Medium |
| Angola | Women | Later Adulthood | 6.74 | 17.18 | 10.44 | 2 | 3.41 (3.40 to 3.42) | African | Lower middle income | Medium |
| Antigua and Barbuda | Men | Early Adulthood | 7.58 | 23.36 | 15.78 | 2 | 4.14 (4.12 to 4.16) | Americas | High income | Very High |
| Antigua and Barbuda | Men | Later Adulthood | 7.72 | 23.54 | 15.82 | 2 | 4.07 (4.05 to 4.08) | Americas | High income | Very High |
| Antigua and Barbuda | Women | Early Adulthood | 17.13 | 40.68 | 23.54 | 3 | 3.16 (3.14 to 3.17) | Americas | High income | Very High |
| Antigua and Barbuda | Women | Later Adulthood | 20.39 | 43.86 | 23.47 | 3 | 2.76 (2.74 to 2.77) | Americas | High income | Very High |
| Argentina | Men | Early Adulthood | 11.9 | 29.7 | 17.8 | 2 | 3.36 (3.35 to 3.36) | Americas | Upper middle income | Very High |
| Argentina | Men | Later Adulthood | 18.99 | 41.27 | 22.27 | 2 | 2.83 (2.82 to 2.83) | Americas | Upper middle income | Very High |
| Argentina | Women | Early Adulthood | 10.38 | 30.82 | 20.44 | 2 | 3.98 (3.97 to 3.99) | Americas | Upper middle income | Very High |
| Argentina | Women | Later Adulthood | 21.68 | 43.78 | 22.1 | 2 | 2.54 (2.54 to 2.55) | Americas | Upper middle income | Very High |
| Armenia | Men | Early Adulthood | 4.53 | 13.88 | 9.35 | 2 | 4.22 (4.19 to 4.24) | European | Upper middle income | Very High |
| Armenia | Men | Later Adulthood | 9.09 | 28 | 18.91 | 2 | 4.03 (4.02 to 4.05) | European | Upper middle income | Very High |
| Armenia | Women | Early Adulthood | 13.07 | 19.27 | 6.2 | 3 | 1.62 (1.59 to 1.65) | European | Upper middle income | Very High |
| Armenia | Women | Later Adulthood | 28.14 | 50.76 | 22.62 | 3 | 2.13 (2.12 to 2.14) | European | Upper middle income | Very High |
| Australia | Men | Early Adulthood | 12.61 | 27.74 | 15.12 | 3 | 2.93 (2.91 to 2.95) | Western Pacific | High income | Very High |
| Australia | Men | Later Adulthood | 17.51 | 37.41 | 19.9 | 2 | 2.78 (2.76 to 2.79) | Western Pacific | High income | Very High |
| Australia | Women | Early Adulthood | 12.01 | 26.11 | 14.1 | 2 | 2.91 (2.89 to 2.94) | Western Pacific | High income | Very High |
| Australia | Women | Later Adulthood | 19.95 | 35.72 | 15.77 | 3 | 2.15 (2.14 to 2.16) | Western Pacific | High income | Very High |
| Austria | Men | Early Adulthood | 7.9 | 16.03 | 8.13 | 2 | 2.56 (2.54 to 2.59) | European | High income | Very High |
| Austria | Men | Later Adulthood | 13.18 | 23 | 9.82 | 3 | 2.05 (2.04 to 2.06) | European | High income | Very High |
| Austria | Women | Early Adulthood | 7.1 | 10.42 | 3.31 | 2 | 1.39 (1.37 to 1.42) | European | High income | Very High |
| Austria | Women | Later Adulthood | 16.74 | 17.86 | 1.11 | 1 | 0.23 (0.22 to 0.25) | European | High income | Very High |
| Azerbaijan | Men | Early Adulthood | 4.18 | 12.89 | 8.71 | 2 | 4.12 (4.10 to 4.15) | European | Upper middle income | High |
| Azerbaijan | Men | Later Adulthood | 9.09 | 29.91 | 20.82 | 1 | 4.32 (4.29 to 4.35) | European | Upper middle income | High |
| Azerbaijan | Women | Early Adulthood | 12.44 | 23.86 | 11.42 | 2 | 2.38 (2.34 to 2.41) | European | Upper middle income | High |
| Azerbaijan | Women | Later Adulthood | 28.24 | 52.87 | 24.63 | 3 | 2.26 (2.25 to 2.27) | European | Upper middle income | High |
| Bahamas | Men | Early Adulthood | 17.04 | 38.51 | 21.47 | 2 | 2.98 (2.96 to 3.00) | Americas | High income | Very High |
| Bahamas | Men | Later Adulthood | 19.55 | 39.83 | 20.28 | 3 | 2.62 (2.60 to 2.63) | Americas | High income | Very High |
| Bahamas | Women | Early Adulthood | 23.07 | 56.06 | 33 | 3 | 3.23 (3.22 to 3.24) | Americas | High income | Very High |
| Bahamas | Women | Later Adulthood | 26.42 | 49.09 | 22.67 | 3 | 2.25 (2.25 to 2.26) | Americas | High income | Very High |
| Bahrain | Men | Early Adulthood | 14.54 | 31.19 | 16.65 | 2 | 2.81 (2.76 to 2.85) | Eastern Mediterranean | High income | Very High |
| Bahrain | Men | Later Adulthood | 16.16 | 36.97 | 20.81 | 3 | 3.00 (2.97 to 3.03) | Eastern Mediterranean | High income | Very High |
| Bahrain | Women | Early Adulthood | 24.17 | 38.86 | 14.69 | 2 | 1.74 (1.72 to 1.76) | Eastern Mediterranean | High income | Very High |
| Bahrain | Women | Later Adulthood | 29.01 | 54.12 | 25.11 | 2 | 2.25 (2.23 to 2.26) | Eastern Mediterranean | High income | Very High |
| Bangladesh | Men | Early Adulthood | 0.21 | 2.76 | 2.55 | 1 | 9.56 (9.52 to 9.59) | South-East Asia | Lower middle income | Medium |
| Bangladesh | Men | Later Adulthood | 0.35 | 2.21 | 1.86 | 1 | 6.79 (6.77 to 6.81) | South-East Asia | Lower middle income | Medium |
| Bangladesh | Women | Early Adulthood | 0.5 | 6.91 | 6.41 | 3 | 9.70 (9.68 to 9.72) | South-East Asia | Lower middle income | Medium |
| Bangladesh | Women | Later Adulthood | 0.65 | 6.43 | 5.79 | 3 | 8.43 (8.41 to 8.45) | South-East Asia | Lower middle income | Medium |
| Barbados | Men | Early Adulthood | 9.46 | 27.44 | 17.98 | 2 | 3.90 (3.89 to 3.91) | Americas | High income | Very High |
| Barbados | Men | Later Adulthood | 8.02 | 24.86 | 16.84 | 2 | 4.12 (4.10 to 4.14) | Americas | High income | Very High |
| Barbados | Women | Early Adulthood | 23.42 | 48.35 | 24.93 | 3 | 2.63 (2.62 to 2.64) | Americas | High income | Very High |
| Barbados | Women | Later Adulthood | 23.78 | 46.51 | 22.73 | 2 | 2.41 (2.39 to 2.42) | Americas | High income | Very High |
| Belarus | Men | Early Adulthood | 7.8 | 15.59 | 7.78 | 1 | 2.49 (2.45 to 2.52) | European | Upper middle income | Very High |
| Belarus | Men | Later Adulthood | 15.89 | 30.21 | 14.32 | 2 | 2.31 (2.29 to 2.33) | European | Upper middle income | Very High |
| Belarus | Women | Early Adulthood | 15.21 | 16.01 | 0.8 | 3 | 0.22 (0.20 to 0.25) | European | Upper middle income | Very High |
| Belarus | Women | Later Adulthood | 38.55 | 44.08 | 5.52 | 2 | 0.48 (0.47 to 0.48) | European | Upper middle income | Very High |
| Belgium | Men | Early Adulthood | 9.78 | 16.45 | 6.68 | 2 | 1.88 (1.86 to 1.90) | European | High income | Very High |
| Belgium | Men | Later Adulthood | 15.97 | 26.18 | 10.21 | 1 | 1.78 (1.76 to 1.79) | European | High income | Very High |
| Belgium | Women | Early Adulthood | 9.45 | 18.27 | 8.83 | 1 | 2.38 (2.38 to 2.39) | European | High income | Very High |
| Belgium | Women | Later Adulthood | 23.67 | 26.04 | 2.38 | 3 | 0.34 (0.33 to 0.34) | European | High income | Very High |
| Belize | Men | Early Adulthood | 11.41 | 31.31 | 19.9 | 2 | 3.69 (3.68 to 3.70) | Americas | Upper middle income | High |
| Belize | Men | Later Adulthood | 12.1 | 33.25 | 21.15 | 2 | 3.68 (3.67 to 3.70) | Americas | Upper middle income | High |
| Belize | Women | Early Adulthood | 25.26 | 50.94 | 25.68 | 3 | 2.55 (2.54 to 2.56) | Americas | Upper middle income | High |
| Belize | Women | Later Adulthood | 27.29 | 53.12 | 25.82 | 3 | 2.40 (2.39 to 2.41) | Americas | Upper middle income | High |
| Benin | Men | Early Adulthood | 0.91 | 5.39 | 4.48 | 1 | 6.55 (6.50 to 6.61) | African | Lower middle income | Low |
| Benin | Men | Later Adulthood | 1.69 | 8.73 | 7.04 | 1 | 6.03 (5.98 to 6.07) | African | Lower middle income | Low |
| Benin | Women | Early Adulthood | 2.75 | 11.98 | 9.22 | 2 | 5.43 (5.41 to 5.46) | African | Lower middle income | Low |
| Benin | Women | Later Adulthood | 4.71 | 18.59 | 13.88 | 2 | 5.08 (5.06 to 5.10) | African | Lower middle income | Low |
| Bermuda | Men | Early Adulthood | 8.26 | 24.51 | 16.25 | 3 | 4.00 (3.99 to 4.02) | Americas | High income | Very High |
| Bermuda | Men | Later Adulthood | 8.33 | 22.73 | 14.4 | 3 | 3.67 (3.66 to 3.68) | Americas | High income | Very High |
| Bermuda | Women | Early Adulthood | 18.38 | 41.83 | 23.45 | 2 | 3.00 (2.97 to 3.04) | Americas | High income | Very High |
| Bermuda | Women | Later Adulthood | 21.5 | 41.82 | 20.31 | 2 | 2.40 (2.39 to 2.41) | Americas | High income | Very High |
| Bhutan | Men | Early Adulthood | 1.14 | 7.7 | 6.56 | 3 | 7.07 (7.06 to 7.08) | South-East Asia | Lower middle income | Medium |
| Bhutan | Men | Later Adulthood | 2.08 | 10.23 | 8.14 | 2 | 5.85 (5.85 to 5.86) | South-East Asia | Lower middle income | Medium |
| Bhutan | Women | Early Adulthood | 2.25 | 14.1 | 11.85 | 1 | 6.79 (6.77 to 6.81) | South-East Asia | Lower middle income | Medium |
| Bhutan | Women | Later Adulthood | 3.24 | 15.02 | 11.78 | 3 | 5.66 (5.65 to 5.68) | South-East Asia | Lower middle income | Medium |
| Bolivia | Men | Early Adulthood | 5.39 | 20.96 | 15.57 | 2 | 4.97 (4.96 to 4.98) | Americas | Lower middle income | High |
| Bolivia | Men | Later Adulthood | 8.24 | 22.66 | 14.41 | 2 | 3.67 (3.67 to 3.68) | Americas | Lower middle income | High |
| Bolivia | Women | Early Adulthood | 9.81 | 29.94 | 20.13 | 2 | 4.07 (4.06 to 4.08) | Americas | Lower middle income | High |
| Bolivia | Women | Later Adulthood | 16.99 | 39.05 | 22.06 | 3 | 3.01 (3.01 to 3.02) | Americas | Lower middle income | High |
| Bosnia and Herzegovina | Men | Early Adulthood | 7.07 | 18 | 10.93 | 1 | 3.41 (3.34 to 3.48) | European | Upper middle income | Very High |
| Bosnia and Herzegovina | Men | Later Adulthood | 13.72 | 30.07 | 16.35 | 3 | 2.84 (2.83 to 2.84) | European | Upper middle income | Very High |
| Bosnia and Herzegovina | Women | Early Adulthood | 9.38 | 15.09 | 5.71 | 1 | 1.71 (1.62 to 1.79) | European | Upper middle income | Very High |
| Bosnia and Herzegovina | Women | Later Adulthood | 29.48 | 36.26 | 6.79 | 2 | 0.75 (0.75 to 0.76) | European | Upper middle income | Very High |
| Botswana | Men | Early Adulthood | 1.66 | 6.62 | 4.96 | 2 | 5.09 (5.06 to 5.12) | African | Upper middle income | High |
| Botswana | Men | Later Adulthood | 3.03 | 10.2 | 7.17 | 1 | 4.42 (4.39 to 4.45) | African | Upper middle income | High |
| Botswana | Women | Early Adulthood | 8.18 | 23.19 | 15.01 | 3 | 3.84 (3.82 to 3.85) | African | Upper middle income | High |
| Botswana | Women | Later Adulthood | 14.79 | 32.17 | 17.39 | 2 | 2.83 (2.81 to 2.84) | African | Upper middle income | High |
| Brazil | Men | Early Adulthood | 5.44 | 22.48 | 17.04 | 3 | 5.23 (5.22 to 5.24) | Americas | Upper middle income | High |
| Brazil | Men | Later Adulthood | 8.4 | 25.07 | 16.67 | 1 | 4.01 (4.00 to 4.02) | Americas | Upper middle income | High |
| Brazil | Women | Early Adulthood | 9.24 | 28.41 | 19.17 | 3 | 4.07 (4.06 to 4.08) | Americas | Upper middle income | High |
| Brazil | Women | Later Adulthood | 18.21 | 35.77 | 17.56 | 3 | 2.41 (2.41 to 2.42) | Americas | Upper middle income | High |
| Brunei Darussalam | Men | Early Adulthood | 6.88 | 30.68 | 23.81 | 3 | 5.51 (5.49 to 5.53) | Western Pacific | High income | Very High |
| Brunei Darussalam | Men | Later Adulthood | 8.02 | 25.37 | 17.35 | 1 | 4.20 (4.17 to 4.23) | Western Pacific | High income | Very High |
| Brunei Darussalam | Women | Early Adulthood | 10.65 | 33.9 | 23.26 | 3 | 4.25 (4.23 to 4.26) | Western Pacific | High income | Very High |
| Brunei Darussalam | Women | Later Adulthood | 12.65 | 30.77 | 18.12 | 3 | 3.21 (3.20 to 3.22) | Western Pacific | High income | Very High |
| Bulgaria | Men | Early Adulthood | 10.03 | 21.21 | 11.18 | 3 | 2.70 (2.69 to 2.71) | European | High income | Very High |
| Bulgaria | Men | Later Adulthood | 15.05 | 30.61 | 15.56 | 1 | 2.57 (2.55 to 2.59) | European | High income | Very High |
| Bulgaria | Women | Early Adulthood | 8.84 | 14.23 | 5.39 | 2 | 1.70 (1.68 to 1.72) | European | High income | Very High |
| Bulgaria | Women | Later Adulthood | 23.33 | 28.55 | 5.22 | 2 | 0.69 (0.68 to 0.69) | European | High income | Very High |
| Burkina Faso | Men | Early Adulthood | 0.36 | 3.04 | 2.68 | 1 | 7.97 (7.92 to 8.02) | African | Low income | Low |
| Burkina Faso | Men | Later Adulthood | 0.63 | 4.64 | 4.01 | 1 | 7.41 (7.37 to 7.44) | African | Low income | Low |
| Burkina Faso | Women | Early Adulthood | 0.8 | 7.77 | 6.97 | 1 | 8.47 (8.44 to 8.50) | African | Low income | Low |
| Burkina Faso | Women | Later Adulthood | 1.11 | 9.27 | 8.16 | 1 | 7.89 (7.86 to 7.91) | African | Low income | Low |
| Burundi | Men | Early Adulthood | 0.84 | 4.5 | 3.66 | 3 | 6.27 (6.25 to 6.29) | African | Low income | Low |
| Burundi | Men | Later Adulthood | 1.64 | 7.39 | 5.75 | 2 | 5.58 (5.57 to 5.60) | African | Low income | Low |
| Burundi | Women | Early Adulthood | 0.74 | 3.55 | 2.81 | 2 | 5.79 (5.77 to 5.81) | African | Low income | Low |
| Burundi | Women | Later Adulthood | 1.12 | 4.1 | 2.98 | 1 | 4.76 (4.73 to 4.79) | African | Low income | Low |
| Cabo Verde | Men | Early Adulthood | 1.32 | 6.48 | 5.15 | 2 | 5.89 (5.82 to 5.95) | African | Lower middle income | Medium |
| Cabo Verde | Men | Later Adulthood | 2.65 | 11.37 | 8.72 | 2 | 5.35 (5.33 to 5.38) | African | Lower middle income | Medium |
| Cabo Verde | Women | Early Adulthood | 4.5 | 20.15 | 15.65 | 3 | 5.61 (5.59 to 5.64) | African | Lower middle income | Medium |
| Cabo Verde | Women | Later Adulthood | 6.19 | 24.91 | 18.73 | 2 | 5.12 (5.10 to 5.13) | African | Lower middle income | Medium |
| Cambodia | Men | Early Adulthood | 0.31 | 2.72 | 2.41 | 3 | 8.14 (8.11 to 8.16) | Western Pacific | Lower middle income | Medium |
| Cambodia | Men | Later Adulthood | 0.5 | 2.88 | 2.38 | 1 | 6.45 (6.43 to 6.47) | Western Pacific | Lower middle income | Medium |
| Cambodia | Women | Early Adulthood | 0.61 | 4.91 | 4.31 | 3 | 7.81 (7.77 to 7.85) | Western Pacific | Lower middle income | Medium |
| Cambodia | Women | Later Adulthood | 0.92 | 5.6 | 4.67 | 2 | 6.58 (6.56 to 6.60) | Western Pacific | Lower middle income | Medium |
| Cameroon | Men | Early Adulthood | 1.67 | 6.68 | 5.01 | 1 | 5.07 (5.02 to 5.12) | African | Lower middle income | Medium |
| Cameroon | Men | Later Adulthood | 3.58 | 12.96 | 9.39 | 2 | 4.72 (4.70 to 4.75) | African | Lower middle income | Medium |
| Cameroon | Women | Early Adulthood | 4.31 | 18.3 | 13.99 | 2 | 5.33 (5.31 to 5.35) | African | Lower middle income | Medium |
| Cameroon | Women | Later Adulthood | 6.12 | 22.14 | 16.02 | 2 | 4.73 (4.71 to 4.75) | African | Lower middle income | Medium |
| Canada | Men | Early Adulthood | 13.93 | 25.68 | 11.74 | 2 | 2.27 (2.24 to 2.30) | Americas | High income | Very High |
| Canada | Men | Later Adulthood | 19.25 | 31.8 | 12.55 | 2 | 1.83 (1.81 to 1.85) | Americas | High income | Very High |
| Canada | Women | Early Adulthood | 13.16 | 23.45 | 10.29 | 3 | 2.17 (2.14 to 2.20) | Americas | High income | Very High |
| Canada | Women | Later Adulthood | 20.25 | 28.52 | 8.27 | 3 | 1.26 (1.25 to 1.28) | Americas | High income | Very High |
| Central African Republic | Men | Early Adulthood | 0.98 | 4.16 | 3.19 | 2 | 5.37 (5.32 to 5.42) | African | Low income | Low |
| Central African Republic | Men | Later Adulthood | 1.77 | 7.2 | 5.43 | 1 | 5.13 (5.09 to 5.16) | African | Low income | Low |
| Central African Republic | Women | Early Adulthood | 1.8 | 9.36 | 7.56 | 3 | 6.12 (6.10 to 6.14) | African | Low income | Low |
| Central African Republic | Women | Later Adulthood | 3.11 | 14.32 | 11.21 | 2 | 5.60 (5.59 to 5.61) | African | Low income | Low |
| Chad | Men | Early Adulthood | 0.93 | 5.35 | 4.42 | 1 | 6.44 (6.38 to 6.50) | African | Low income | Low |
| Chad | Men | Later Adulthood | 1.87 | 9.52 | 7.65 | 1 | 5.98 (5.94 to 6.02) | African | Low income | Low |
| Chad | Women | Early Adulthood | 0.8 | 4.73 | 3.93 | 1 | 6.56 (6.51 to 6.61) | African | Low income | Low |
| Chad | Women | Later Adulthood | 1.23 | 7.13 | 5.9 | 1 | 6.48 (6.45 to 6.52) | African | Low income | Low |
| Chile | Men | Early Adulthood | 8.9 | 31.2 | 22.3 | 2 | 4.68 (4.67 to 4.70) | Americas | High income | Very High |
| Chile | Men | Later Adulthood | 16.44 | 34.2 | 17.76 | 1 | 2.70 (2.68 to 2.72) | Americas | High income | Very High |
| Chile | Women | Early Adulthood | 14.03 | 41.2 | 27.17 | 3 | 3.98 (3.96 to 3.99) | Americas | High income | Very High |
| Chile | Women | Later Adulthood | 27.41 | 46.88 | 19.47 | 3 | 1.95 (1.94 to 1.96) | Americas | High income | Very High |
| China | Men | Early Adulthood | 0.75 | 9.1 | 8.35 | 3 | 9.36 (9.34 to 9.39) | Western Pacific | Upper middle income | High |
| China | Men | Later Adulthood | 1.29 | 5.48 | 4.19 | 0 | 5.29 (5.27 to 5.32) | Western Pacific | Upper middle income | High |
| China | Women | Early Adulthood | 1.24 | 6.97 | 5.72 | 3 | 6.37 (6.36 to 6.38) | Western Pacific | Upper middle income | High |
| China | Women | Later Adulthood | 3.57 | 7.78 | 4.21 | 2 | 2.80 (2.79 to 2.81) | Western Pacific | Upper middle income | High |
| Colombia | Men | Early Adulthood | 4.16 | 16.5 | 12.34 | 3 | 5.06 (5.04 to 5.08) | Americas | Upper middle income | High |
| Colombia | Men | Later Adulthood | 6.49 | 19.96 | 13.48 | 3 | 4.08 (4.06 to 4.09) | Americas | Upper middle income | High |
| Colombia | Women | Early Adulthood | 8.34 | 25.47 | 17.13 | 3 | 4.11 (4.09 to 4.13) | Americas | Upper middle income | High |
| Colombia | Women | Later Adulthood | 17.92 | 34.81 | 16.89 | 3 | 2.40 (2.39 to 2.41) | Americas | Upper middle income | High |
| Comoros | Men | Early Adulthood | 1.23 | 6.54 | 5.3 | 3 | 6.21 (6.18 to 6.24) | African | Lower middle income | Medium |
| Comoros | Men | Later Adulthood | 2.23 | 8.9 | 6.67 | 3 | 5.10 (5.08 to 5.11) | African | Lower middle income | Medium |
| Comoros | Women | Early Adulthood | 4.52 | 22.32 | 17.79 | 2 | 5.83 (5.81 to 5.86) | African | Lower middle income | Medium |
| Comoros | Women | Later Adulthood | 6.73 | 24.77 | 18.04 | 3 | 4.70 (4.69 to 4.72) | African | Lower middle income | Medium |
| Congo | Men | Early Adulthood | 0.92 | 3.15 | 2.23 | 1 | 4.49 (4.46 to 4.53) | African | Lower middle income | Medium |
| Congo | Men | Later Adulthood | 2.08 | 5.81 | 3.73 | 2 | 3.74 (3.71 to 3.77) | African | Lower middle income | Medium |
| Congo | Women | Early Adulthood | 3.81 | 11.75 | 7.94 | 3 | 4.17 (4.13 to 4.20) | African | Lower middle income | Medium |
| Congo | Women | Later Adulthood | 6.6 | 15.44 | 8.84 | 3 | 3.11 (3.09 to 3.13) | African | Lower middle income | Medium |
| Costa Rica | Men | Early Adulthood | 9.5 | 24.85 | 15.34 | 3 | 3.55 (3.54 to 3.57) | Americas | Upper middle income | Very High |
| Costa Rica | Men | Later Adulthood | 11.96 | 23.89 | 11.92 | 3 | 2.52 (2.51 to 2.53) | Americas | Upper middle income | Very High |
| Costa Rica | Women | Early Adulthood | 11.97 | 34.73 | 22.75 | 3 | 3.93 (3.91 to 3.94) | Americas | Upper middle income | Very High |
| Costa Rica | Women | Later Adulthood | 20.45 | 42.82 | 22.37 | 3 | 2.68 (2.67 to 2.69) | Americas | Upper middle income | Very High |
| Cote d'Ivoire | Men | Early Adulthood | 1.15 | 6.43 | 5.28 | 1 | 6.36 (6.32 to 6.41) | African | Lower middle income | Medium |
| Cote d'Ivoire | Men | Later Adulthood | 2 | 8.9 | 6.91 | 1 | 5.47 (5.43 to 5.52) | African | Lower middle income | Medium |
| Cote d'Ivoire | Women | Early Adulthood | 3.05 | 13.67 | 10.62 | 1 | 5.51 (5.46 to 5.56) | African | Lower middle income | Medium |
| Cote d'Ivoire | Women | Later Adulthood | 4.53 | 17.58 | 13.05 | 3 | 5.00 (4.98 to 5.01) | African | Lower middle income | Medium |
| Croatia | Men | Early Adulthood | 9.74 | 29.97 | 20.23 | 1 | 4.10 (4.08 to 4.11) | European | High income | Very High |
| Croatia | Men | Later Adulthood | 15.64 | 40.64 | 24.99 | 2 | 3.41 (3.40 to 3.42) | European | High income | Very High |
| Croatia | Women | Early Adulthood | 8.35 | 20.01 | 11.66 | 1 | 3.17 (3.14 to 3.20) | European | High income | Very High |
| Croatia | Women | Later Adulthood | 25.25 | 44.9 | 19.65 | 3 | 2.03 (2.02 to 2.03) | European | High income | Very High |
| Cuba | Men | Early Adulthood | 4.59 | 17.33 | 12.74 | 2 | 4.87 (4.84 to 4.90) | Americas | Upper middle income | High |
| Cuba | Men | Later Adulthood | 5.05 | 20.33 | 15.28 | 3 | 5.09 (5.08 to 5.10) | Americas | Upper middle income | High |
| Cuba | Women | Early Adulthood | 8.89 | 22.84 | 13.95 | 2 | 3.43 (3.40 to 3.47) | Americas | Upper middle income | High |
| Cuba | Women | Later Adulthood | 14.11 | 30.35 | 16.24 | 2 | 2.77 (2.76 to 2.77) | Americas | Upper middle income | High |
| Cyprus | Men | Early Adulthood | 10.73 | 22.1 | 11.37 | 3 | 2.65 (2.63 to 2.67) | European | High income | Very High |
| Cyprus | Men | Later Adulthood | 19.53 | 33.79 | 14.26 | 2 | 1.98 (1.96 to 1.99) | European | High income | Very High |
| Cyprus | Women | Early Adulthood | 10.9 | 15.55 | 4.65 | 3 | 1.30 (1.29 to 1.31) | European | High income | Very High |
| Cyprus | Women | Later Adulthood | 31.23 | 33.13 | 1.89 | 1 | 0.21 (0.20 to 0.22) | European | High income | Very High |
| Czechia | Men | Early Adulthood | 13.77 | 25.34 | 11.57 | 3 | 2.16 (2.14 to 2.18) | European | High income | Very High |
| Czechia | Men | Later Adulthood | 28.16 | 42.82 | 14.65 | 2 | 1.47 (1.46 to 1.47) | European | High income | Very High |
| Czechia | Women | Early Adulthood | 14.24 | 16.77 | 2.53 | 2 | 0.56 (0.53 to 0.58) | European | High income | Very High |
| Czechia | Women | Later Adulthood | 40.54 | 38.78 | -1.77 | 2 | -0.18 (-0.19 to -0.17) | European | High income | Very High |
| DR Congo | Men | Early Adulthood | 0.77 | 2.89 | 2.12 | 1 | 4.82 (4.76 to 4.88) | African | Low income | Low |
| DR Congo | Men | Later Adulthood | 1.63 | 5.59 | 3.97 | 2 | 4.52 (4.48 to 4.56) | African | Low income | Low |
| DR Congo | Women | Early Adulthood | 1.4 | 7.09 | 5.69 | 1 | 5.97 (5.95 to 6.00) | African | Low income | Low |
| DR Congo | Women | Later Adulthood | 2.54 | 10.23 | 7.69 | 1 | 5.10 (5.09 to 5.11) | African | Low income | Low |
| Denmark | Men | Early Adulthood | 7.08 | 13.28 | 6.2 | 2 | 2.29 (2.26 to 2.31) | European | High income | Very High |
| Denmark | Men | Later Adulthood | 13.11 | 20.03 | 6.92 | 2 | 1.54 (1.52 to 1.56) | European | High income | Very High |
| Denmark | Women | Early Adulthood | 5.83 | 10.2 | 4.37 | 1 | 2.02 (2.00 to 2.05) | European | High income | Very High |
| Denmark | Women | Later Adulthood | 13.81 | 13.84 | 0.03 | 2 | 0.01 (0.00 to 0.02) | European | High income | Very High |
| Djibouti | Men | Early Adulthood | 0.85 | 4.64 | 3.79 | 3 | 6.24 (6.23 to 6.26) | Eastern Mediterranean | Lower middle income | Low |
| Djibouti | Men | Later Adulthood | 1.63 | 7.36 | 5.73 | 2 | 5.61 (5.59 to 5.63) | Eastern Mediterranean | Lower middle income | Low |
| Djibouti | Women | Early Adulthood | 3.12 | 14.23 | 11.12 | 3 | 5.50 (5.48 to 5.51) | Eastern Mediterranean | Lower middle income | Low |
| Djibouti | Women | Later Adulthood | 5.19 | 18.22 | 13.03 | 1 | 4.60 (4.58 to 4.62) | Eastern Mediterranean | Lower middle income | Low |
| Dominica | Men | Early Adulthood | 4.72 | 17.61 | 12.89 | 3 | 4.88 (4.86 to 4.91) | Americas | Upper middle income | High |
| Dominica | Men | Later Adulthood | 4.63 | 16.55 | 11.93 | 3 | 4.66 (4.64 to 4.68) | Americas | Upper middle income | High |
| Dominica | Women | Early Adulthood | 18.35 | 43.48 | 25.13 | 2 | 3.16 (3.15 to 3.18) | Americas | Upper middle income | High |
| Dominica | Women | Later Adulthood | 21.86 | 44.84 | 22.98 | 3 | 2.59 (2.58 to 2.61) | Americas | Upper middle income | High |
| Dominican Republic | Men | Early Adulthood | 5.73 | 21.54 | 15.81 | 2 | 4.88 (4.86 to 4.91) | Americas | Upper middle income | High |
| Dominican Republic | Men | Later Adulthood | 6.7 | 24.64 | 17.94 | 3 | 4.78 (4.77 to 4.80) | Americas | Upper middle income | High |
| Dominican Republic | Women | Early Adulthood | 10.72 | 32.86 | 22.15 | 3 | 4.12 (4.10 to 4.14) | Americas | Upper middle income | High |
| Dominican Republic | Women | Later Adulthood | 15.84 | 38.04 | 22.2 | 3 | 3.18 (3.17 to 3.18) | Americas | Upper middle income | High |
| Ecuador | Men | Early Adulthood | 5.06 | 19.67 | 14.61 | 1 | 4.96 (4.95 to 4.96) | Americas | Upper middle income | High |
| Ecuador | Men | Later Adulthood | 8.24 | 24.06 | 15.82 | 1 | 3.90 (3.89 to 3.91) | Americas | Upper middle income | High |
| Ecuador | Women | Early Adulthood | 11.43 | 28.65 | 17.22 | 2 | 3.34 (3.34 to 3.35) | Americas | Upper middle income | High |
| Ecuador | Women | Later Adulthood | 20.05 | 37.48 | 17.43 | 3 | 2.25 (2.25 to 2.26) | Americas | Upper middle income | High |
| Egypt | Men | Early Adulthood | 12 | 26.93 | 14.93 | 3 | 2.94 (2.92 to 2.96) | Eastern Mediterranean | Lower middle income | High |
| Egypt | Men | Later Adulthood | 17.23 | 40.07 | 22.83 | 3 | 3.05 (3.04 to 3.07) | Eastern Mediterranean | Lower middle income | High |
| Egypt | Women | Early Adulthood | 25.8 | 50.64 | 24.83 | 2 | 2.46 (2.44 to 2.47) | Eastern Mediterranean | Lower middle income | High |
| Egypt | Women | Later Adulthood | 32.47 | 70.57 | 38.09 | 3 | 2.81 (2.80 to 2.82) | Eastern Mediterranean | Lower middle income | High |
| El Salvador | Men | Early Adulthood | 8.28 | 21.62 | 13.35 | 2 | 3.52 (3.50 to 3.54) | Americas | Upper middle income | Medium |
| El Salvador | Men | Later Adulthood | 10.99 | 21.88 | 10.9 | 2 | 2.52 (2.50 to 2.54) | Americas | Upper middle income | Medium |
| El Salvador | Women | Early Adulthood | 14.18 | 35.4 | 21.22 | 3 | 3.35 (3.33 to 3.36) | Americas | Upper middle income | Medium |
| El Salvador | Women | Later Adulthood | 21.84 | 38.38 | 16.54 | 3 | 2.06 (2.04 to 2.07) | Americas | Upper middle income | Medium |
| Equatorial Guinea | Men | Early Adulthood | 1.54 | 6.31 | 4.77 | 3 | 5.28 (5.26 to 5.29) | African | Upper middle income | Medium |
| Equatorial Guinea | Men | Later Adulthood | 2.83 | 9.88 | 7.05 | 1 | 4.57 (4.53 to 4.60) | African | Upper middle income | Medium |
| Equatorial Guinea | Women | Early Adulthood | 9.15 | 27.81 | 18.66 | 3 | 4.11 (4.09 to 4.13) | African | Upper middle income | Medium |
| Equatorial Guinea | Women | Later Adulthood | 12.86 | 31.27 | 18.41 | 3 | 3.24 (3.23 to 3.25) | African | Upper middle income | Medium |
| Eritrea | Men | Early Adulthood | 0.4 | 2.05 | 1.65 | 3 | 6.08 (6.04 to 6.11) | African | Low income | Low |
| Eritrea | Men | Later Adulthood | 0.71 | 3.06 | 2.35 | 2 | 5.47 (5.44 to 5.50) | African | Low income | Low |
| Eritrea | Women | Early Adulthood | 0.73 | 4.82 | 4.09 | 3 | 6.84 (6.83 to 6.86) | African | Low income | Low |
| Eritrea | Women | Later Adulthood | 1.52 | 7.89 | 6.37 | 3 | 5.99 (5.98 to 6.00) | African | Low income | Low |
| Estonia | Men | Early Adulthood | 9.81 | 20.09 | 10.28 | 1 | 2.59 (2.57 to 2.61) | European | High income | Very High |
| Estonia | Men | Later Adulthood | 16.95 | 31.37 | 14.41 | 1 | 2.23 (2.20 to 2.25) | European | High income | Very High |
| Estonia | Women | Early Adulthood | 11.16 | 15.49 | 4.33 | 1 | 1.18 (1.15 to 1.21) | European | High income | Very High |
| Estonia | Women | Later Adulthood | 27.6 | 38.41 | 10.8 | 3 | 1.13 (1.12 to 1.14) | European | High income | Very High |
| Eswatini | Men | Early Adulthood | 2.34 | 10.67 | 8.33 | 3 | 5.59 (5.57 to 5.60) | African | Lower middle income | Medium |
| Eswatini | Men | Later Adulthood | 4.77 | 19.36 | 14.59 | 1 | 5.13 (5.10 to 5.16) | African | Lower middle income | Medium |
| Eswatini | Women | Early Adulthood | 13.47 | 36.93 | 23.47 | 3 | 3.70 (3.69 to 3.71) | African | Lower middle income | Medium |
| Eswatini | Women | Later Adulthood | 24.27 | 53.66 | 29.4 | 2 | 2.88 (2.86 to 2.89) | African | Lower middle income | Medium |
| Ethiopia | Men | Early Adulthood | 0.15 | 0.68 | 0.53 | 2 | 5.81 (5.78 to 5.84) | African | Low income | Low |
| Ethiopia | Men | Later Adulthood | 0.33 | 1.35 | 1.02 | 2 | 5.33 (5.30 to 5.35) | African | Low income | Low |
| Ethiopia | Women | Early Adulthood | 0.4 | 3.21 | 2.82 | 2 | 7.59 (7.56 to 7.62) | African | Low income | Low |
| Ethiopia | Women | Later Adulthood | 0.92 | 5.31 | 4.39 | 3 | 6.37 (6.35 to 6.39) | African | Low income | Low |
| Federated States of Micronesia | Men | Early Adulthood | 24.43 | 36.55 | 12.11 | 1 | 1.41 (1.35 to 1.46) | Western Pacific | Lower middle income | Medium |
| Federated States of Micronesia | Men | Later Adulthood | 26.55 | 38.95 | 12.4 | 1 | 1.40 (1.36 to 1.45) | Western Pacific | Lower middle income | Medium |
| Federated States of Micronesia | Women | Early Adulthood | 42.71 | 53.55 | 10.84 | 2 | 0.84 (0.82 to 0.85) | Western Pacific | Lower middle income | Medium |
| Federated States of Micronesia | Women | Later Adulthood | 44.44 | 58.67 | 14.23 | 1 | 0.99 (0.98 to 1.01) | Western Pacific | Lower middle income | Medium |
| Fiji | Men | Early Adulthood | 11.1 | 23.43 | 12.33 | 3 | 2.71 (2.69 to 2.72) | Western Pacific | Upper middle income | High |
| Fiji | Men | Later Adulthood | 15.67 | 30 | 14.33 | 2 | 2.32 (2.32 to 2.33) | Western Pacific | Upper middle income | High |
| Fiji | Women | Early Adulthood | 24.58 | 39.48 | 14.9 | 3 | 1.74 (1.74 to 1.74) | Western Pacific | Upper middle income | High |
| Fiji | Women | Later Adulthood | 32.79 | 47.48 | 14.7 | 1 | 1.32 (1.32 to 1.33) | Western Pacific | Upper middle income | High |
| Finland | Men | Early Adulthood | 10.99 | 19.22 | 8.23 | 3 | 2.10 (2.07 to 2.12) | European | High income | Very High |
| Finland | Men | Later Adulthood | 19.85 | 25.97 | 6.12 | 1 | 0.96 (0.95 to 0.98) | European | High income | Very High |
| Finland | Women | Early Adulthood | 9.48 | 18.4 | 8.91 | 2 | 2.47 (2.45 to 2.48) | European | High income | Very High |
| Finland | Women | Later Adulthood | 28.39 | 29.61 | 1.22 | 1 | 0.15 (0.14 to 0.15) | European | High income | Very High |
| France | Men | Early Adulthood | 7.02 | 8.19 | 1.17 | 2 | 0.55 (0.52 to 0.58) | European | High income | Very High |
| France | Men | Later Adulthood | 16.19 | 15.85 | -0.34 | 2 | -0.09 (-0.10 to -0.07) | European | High income | Very High |
| France | Women | Early Adulthood | 8.32 | 9.01 | 0.69 | 3 | 0.29 (0.27 to 0.30) | European | High income | Very High |
| France | Women | Later Adulthood | 19.5 | 12.91 | -6.6 | 2 | -1.48 (-1.49 to -1.47) | European | High income | Very High |
| French Polynesia | Men | Early Adulthood | 33.09 | 47.22 | 14.13 | 3 | 1.32 (1.31 to 1.32) | Western Pacific | High income | Very High |
| French Polynesia | Men | Later Adulthood | 35.21 | 45.68 | 10.47 | 3 | 0.97 (0.96 to 0.97) | Western Pacific | High income | Very High |
| French Polynesia | Women | Early Adulthood | 37.91 | 52.97 | 15.06 | 2 | 1.22 (1.21 to 1.23) | Western Pacific | High income | Very High |
| French Polynesia | Women | Later Adulthood | 37.29 | 45.66 | 8.37 | 2 | 0.74 (0.74 to 0.75) | Western Pacific | High income | Very High |
| Gabon | Men | Early Adulthood | 2.57 | 8.95 | 6.38 | 3 | 4.61 (4.59 to 4.62) | African | Upper middle income | High |
| Gabon | Men | Later Adulthood | 4.55 | 13.92 | 9.38 | 2 | 4.09 (4.07 to 4.11) | African | Upper middle income | High |
| Gabon | Women | Early Adulthood | 7.56 | 27.75 | 20.19 | 2 | 4.76 (4.75 to 4.77) | African | Upper middle income | High |
| Gabon | Women | Later Adulthood | 11.83 | 36.15 | 24.32 | 2 | 4.07 (4.06 to 4.08) | African | Upper middle income | High |
| Gambia | Men | Early Adulthood | 0.61 | 7.38 | 6.76 | 1 | 9.31 (9.24 to 9.38) | African | Low income | Low |
| Gambia | Men | Later Adulthood | 1.06 | 10.6 | 9.54 | 1 | 8.58 (8.52 to 8.63) | African | Low income | Low |
| Gambia | Women | Early Adulthood | 2.41 | 15.42 | 13.01 | 1 | 6.87 (6.82 to 6.92) | African | Low income | Low |
| Gambia | Women | Later Adulthood | 3.26 | 24.11 | 20.85 | 2 | 7.42 (7.40 to 7.45) | African | Low income | Low |
| Georgia | Men | Early Adulthood | 9.81 | 29.03 | 19.23 | 0 | 3.96 (3.94 to 3.97) | European | Upper middle income | Very High |
| Georgia | Men | Later Adulthood | 14.58 | 40.3 | 25.73 | 2 | 3.63 (3.61 to 3.65) | European | Upper middle income | Very High |
| Georgia | Women | Early Adulthood | 13.11 | 27.14 | 14.03 | 3 | 2.68 (2.66 to 2.70) | European | Upper middle income | Very High |
| Georgia | Women | Later Adulthood | 26.48 | 56.5 | 30.02 | 3 | 2.72 (2.71 to 2.73) | European | Upper middle income | Very High |
| Germany | Men | Early Adulthood | 11.33 | 18.74 | 7.42 | 2 | 1.84 (1.81 to 1.86) | European | High income | Very High |
| Germany | Men | Later Adulthood | 21.02 | 32.3 | 11.29 | 2 | 1.56 (1.54 to 1.58) | European | High income | Very High |
| Germany | Women | Early Adulthood | 9.47 | 15 | 5.53 | 2 | 1.69 (1.66 to 1.72) | European | High income | Very High |
| Germany | Women | Later Adulthood | 27.31 | 29.15 | 1.83 | 2 | 0.24 (0.23 to 0.25) | European | High income | Very High |
| Ghana | Men | Early Adulthood | 0.93 | 4.14 | 3.21 | 1 | 5.46 (5.39 to 5.53) | African | Lower middle income | Medium |
| Ghana | Men | Later Adulthood | 1.6 | 6.67 | 5.07 | 1 | 5.21 (5.16 to 5.26) | African | Lower middle income | Medium |
| Ghana | Women | Early Adulthood | 4.15 | 17.92 | 13.76 | 2 | 5.40 (5.37 to 5.42) | African | Lower middle income | Medium |
| Ghana | Women | Later Adulthood | 7.88 | 23.81 | 15.93 | 2 | 4.07 (4.05 to 4.08) | African | Lower middle income | Medium |
| Greece | Men | Early Adulthood | 8.8 | 25.13 | 16.33 | 1 | 3.81 (3.78 to 3.85) | European | High income | Very High |
| Greece | Men | Later Adulthood | 16.65 | 39.31 | 22.66 | 2 | 3.12 (3.10 to 3.13) | European | High income | Very High |
| Greece | Women | Early Adulthood | 13.15 | 21.32 | 8.17 | 3 | 1.69 (1.68 to 1.70) | European | High income | Very High |
| Greece | Women | Later Adulthood | 35.72 | 44.6 | 8.88 | 1 | 0.80 (0.79 to 0.80) | European | High income | Very High |
| Greenland | Men | Early Adulthood | 9.61 | 20.77 | 11.15 | 2 | 2.81 (2.79 to 2.84) | European | High income | Very High |
| Greenland | Men | Later Adulthood | 16.49 | 29.96 | 13.47 | 1 | 2.15 (2.13 to 2.17) | European | High income | Very High |
| Greenland | Women | Early Adulthood | 13.49 | 28.39 | 14.9 | 2 | 2.73 (2.72 to 2.75) | European | High income | Very High |
| Greenland | Women | Later Adulthood | 25.97 | 34.33 | 8.37 | 2 | 1.01 (1.00 to 1.01) | European | High income | Very High |
| Grenada | Men | Early Adulthood | 5.6 | 18.18 | 12.58 | 3 | 4.39 (4.37 to 4.40) | Americas | Upper middle income | High |
| Grenada | Men | Later Adulthood | 5.88 | 17.37 | 11.5 | 2 | 3.94 (3.93 to 3.95) | Americas | Upper middle income | High |
| Grenada | Women | Early Adulthood | 16.32 | 39.43 | 23.11 | 2 | 3.27 (3.25 to 3.29) | Americas | Upper middle income | High |
| Grenada | Women | Later Adulthood | 21.64 | 43.99 | 22.35 | 3 | 2.55 (2.54 to 2.56) | Americas | Upper middle income | High |
| Guatemala | Men | Early Adulthood | 5.13 | 19.54 | 14.41 | 3 | 4.90 (4.89 to 4.91) | Americas | Upper middle income | Medium |
| Guatemala | Men | Later Adulthood | 8.03 | 22.26 | 14.23 | 3 | 3.71 (3.70 to 3.71) | Americas | Upper middle income | Medium |
| Guatemala | Women | Early Adulthood | 9.32 | 28.06 | 18.74 | 3 | 4.06 (4.04 to 4.08) | Americas | Upper middle income | Medium |
| Guatemala | Women | Later Adulthood | 13.28 | 33.89 | 20.61 | 3 | 3.40 (3.39 to 3.41) | Americas | Upper middle income | Medium |
| Guinea | Men | Early Adulthood | 0.7 | 3.97 | 3.28 | 1 | 6.41 (6.34 to 6.49) | African | Lower middle income | Low |
| Guinea | Men | Later Adulthood | 1.35 | 7.62 | 6.26 | 1 | 6.36 (6.32 to 6.40) | African | Lower middle income | Low |
| Guinea | Women | Early Adulthood | 1.56 | 10.17 | 8.61 | 1 | 6.94 (6.91 to 6.98) | African | Lower middle income | Low |
| Guinea | Women | Later Adulthood | 2.38 | 13.77 | 11.39 | 1 | 6.48 (6.45 to 6.50) | African | Lower middle income | Low |
| Guinea Bissau | Men | Early Adulthood | 1.06 | 5.88 | 4.82 | 1 | 6.32 (6.28 to 6.36) | African | Low income | Low |
| Guinea Bissau | Men | Later Adulthood | 1.72 | 9.15 | 7.42 | 1 | 6.14 (6.09 to 6.18) | African | Low income | Low |
| Guinea Bissau | Women | Early Adulthood | 2.52 | 11.72 | 9.2 | 1 | 5.67 (5.64 to 5.70) | African | Low income | Low |
| Guinea Bissau | Women | Later Adulthood | 3.79 | 18.15 | 14.36 | 1 | 5.77 (5.73 to 5.81) | African | Low income | Low |
| Guyana | Men | Early Adulthood | 4.76 | 16.68 | 11.92 | 2 | 4.60 (4.58 to 4.61) | Americas | High income | High |
| Guyana | Men | Later Adulthood | 5.15 | 16.9 | 11.74 | 2 | 4.35 (4.33 to 4.36) | Americas | High income | High |
| Guyana | Women | Early Adulthood | 12.88 | 34.71 | 21.83 | 2 | 3.62 (3.61 to 3.63) | Americas | High income | High |
| Guyana | Women | Later Adulthood | 19.21 | 42.08 | 22.87 | 2 | 2.84 (2.83 to 2.85) | Americas | High income | High |
| Haiti | Men | Early Adulthood | 1.97 | 6 | 4.02 | 1 | 4.06 (4.02 to 4.10) | Americas | Lower middle income | Medium |
| Haiti | Men | Later Adulthood | 2.38 | 6.82 | 4.44 | 2 | 3.87 (3.84 to 3.89) | Americas | Lower middle income | Medium |
| Haiti | Women | Early Adulthood | 4.44 | 13.19 | 8.75 | 2 | 4.00 (3.99 to 4.01) | Americas | Lower middle income | Medium |
| Haiti | Women | Later Adulthood | 6.21 | 16.31 | 10.1 | 2 | 3.53 (3.52 to 3.55) | Americas | Lower middle income | Medium |
| Honduras | Men | Early Adulthood | 7.1 | 21.32 | 14.22 | 3 | 4.02 (4.01 to 4.03) | Americas | Lower middle income | Medium |
| Honduras | Men | Later Adulthood | 9.73 | 24.04 | 14.31 | 3 | 3.30 (3.29 to 3.31) | Americas | Lower middle income | Medium |
| Honduras | Women | Early Adulthood | 9.41 | 32.81 | 23.39 | 3 | 4.58 (4.56 to 4.60) | Americas | Lower middle income | Medium |
| Honduras | Women | Later Adulthood | 15.78 | 39.69 | 23.91 | 3 | 3.35 (3.33 to 3.36) | Americas | Lower middle income | Medium |
| Hungary | Men | Early Adulthood | 12.08 | 30.89 | 18.81 | 2 | 3.40 (3.39 to 3.41) | European | High income | Very High |
| Hungary | Men | Later Adulthood | 17.38 | 45.32 | 27.94 | 2 | 3.49 (3.48 to 3.50) | European | High income | Very High |
| Hungary | Women | Early Adulthood | 11.14 | 21.36 | 10.22 | 3 | 2.33 (2.31 to 2.34) | European | High income | Very High |
| Hungary | Women | Later Adulthood | 25.22 | 46.02 | 20.8 | 3 | 2.14 (2.13 to 2.15) | European | High income | Very High |
| Iceland | Men | Early Adulthood | 8.34 | 19.07 | 10.73 | 2 | 3.03 (3.00 to 3.06) | European | High income | Very High |
| Iceland | Men | Later Adulthood | 14.8 | 30.82 | 16.02 | 2 | 2.66 (2.65 to 2.68) | European | High income | Very High |
| Iceland | Women | Early Adulthood | 8.13 | 16.89 | 8.76 | 2 | 2.70 (2.67 to 2.72) | European | High income | Very High |
| Iceland | Women | Later Adulthood | 18.18 | 24.97 | 6.8 | 2 | 1.16 (1.15 to 1.17) | European | High income | Very High |
| India | Men | Early Adulthood | 0.49 | 4.63 | 4.15 | 2 | 8.38 (8.36 to 8.40) | South-East Asia | Lower middle income | Medium |
| India | Men | Later Adulthood | 0.69 | 5.13 | 4.44 | 3 | 7.36 (7.33 to 7.38) | South-East Asia | Lower middle income | Medium |
| India | Women | Early Adulthood | 1.08 | 8.05 | 6.96 | 2 | 7.49 (7.46 to 7.51) | South-East Asia | Lower middle income | Medium |
| India | Women | Later Adulthood | 1.88 | 10.37 | 8.49 | 3 | 6.31 (6.29 to 6.32) | South-East Asia | Lower middle income | Medium |
| Indonesia | Men | Early Adulthood | 0.66 | 6.51 | 5.85 | 2 | 8.42 (8.39 to 8.44) | South-East Asia | Upper middle income | High |
| Indonesia | Men | Later Adulthood | 0.65 | 4.47 | 3.82 | 2 | 7.00 (6.98 to 7.03) | South-East Asia | Upper middle income | High |
| Indonesia | Women | Early Adulthood | 2.53 | 16.5 | 13.97 | 3 | 6.82 (6.80 to 6.84) | South-East Asia | Upper middle income | High |
| Indonesia | Women | Later Adulthood | 2.8 | 12.57 | 9.77 | 1 | 5.50 (5.44 to 5.56) | South-East Asia | Upper middle income | High |
| Iran | Men | Early Adulthood | 5.52 | 17.28 | 11.76 | 1 | 4.24 (4.20 to 4.29) | Eastern Mediterranean | Upper middle income | High |
| Iran | Men | Later Adulthood | 7.63 | 19.06 | 11.44 | 2 | 3.33 (3.31 to 3.35) | Eastern Mediterranean | Upper middle income | High |
| Iran | Women | Early Adulthood | 15 | 27.67 | 12.67 | 2 | 2.29 (2.27 to 2.31) | Eastern Mediterranean | Upper middle income | High |
| Iran | Women | Later Adulthood | 21.1 | 40.57 | 19.47 | 2 | 2.37 (2.36 to 2.39) | Eastern Mediterranean | Upper middle income | High |
| Iraq | Men | Early Adulthood | 12.51 | 27.28 | 14.77 | 3 | 2.80 (2.79 to 2.81) | Eastern Mediterranean | Upper middle income | Medium |
| Iraq | Men | Later Adulthood | 16.75 | 40.04 | 23.29 | 3 | 3.17 (3.16 to 3.19) | Eastern Mediterranean | Upper middle income | Medium |
| Iraq | Women | Early Adulthood | 22.97 | 41.01 | 18.04 | 2 | 2.09 (2.08 to 2.10) | Eastern Mediterranean | Upper middle income | Medium |
| Iraq | Women | Later Adulthood | 30.57 | 59.9 | 29.33 | 2 | 2.42 (2.41 to 2.43) | Eastern Mediterranean | Upper middle income | Medium |
| Ireland | Men | Early Adulthood | 12.74 | 26.11 | 13.36 | 2 | 2.61 (2.58 to 2.63) | European | High income | Very High |
| Ireland | Men | Later Adulthood | 18.79 | 40.43 | 21.64 | 2 | 2.80 (2.78 to 2.81) | European | High income | Very High |
| Ireland | Women | Early Adulthood | 11.62 | 24.07 | 12.45 | 3 | 2.67 (2.65 to 2.68) | European | High income | Very High |
| Ireland | Women | Later Adulthood | 20.41 | 35.29 | 14.88 | 3 | 2.00 (1.99 to 2.00) | European | High income | Very High |
| Israel | Men | Early Adulthood | 9.8 | 19.45 | 9.64 | 1 | 2.47 (2.45 to 2.50) | European | High income | Very High |
| Israel | Men | Later Adulthood | 16.98 | 31.06 | 14.07 | 1 | 2.18 (2.15 to 2.20) | European | High income | Very High |
| Israel | Women | Early Adulthood | 12.6 | 17.48 | 4.89 | 3 | 1.20 (1.18 to 1.22) | European | High income | Very High |
| Israel | Women | Later Adulthood | 31.58 | 33.49 | 1.91 | 3 | 0.21 (0.20 to 0.22) | European | High income | Very High |
| Italy | Men | Early Adulthood | 8.58 | 15.54 | 6.96 | 1 | 2.14 (2.08 to 2.19) | European | High income | Very High |
| Italy | Men | Later Adulthood | 16.09 | 24.18 | 8.09 | 1 | 1.46 (1.43 to 1.50) | European | High income | Very High |
| Italy | Women | Early Adulthood | 8.87 | 12.99 | 4.13 | 2 | 1.28 (1.25 to 1.31) | European | High income | Very High |
| Italy | Women | Later Adulthood | 25.44 | 29.6 | 4.16 | 3 | 0.50 (0.48 to 0.51) | European | High income | Very High |
| Jamaica | Men | Early Adulthood | 4.23 | 18.57 | 14.34 | 2 | 5.44 (5.42 to 5.46) | Americas | Upper middle income | High |
| Jamaica | Men | Later Adulthood | 3.79 | 16.94 | 13.15 | 3 | 5.50 (5.49 to 5.52) | Americas | Upper middle income | High |
| Jamaica | Women | Early Adulthood | 17.56 | 45.79 | 28.23 | 2 | 3.50 (3.49 to 3.51) | Americas | Upper middle income | High |
| Jamaica | Women | Later Adulthood | 17.91 | 51.97 | 34.07 | 3 | 3.86 (3.85 to 3.88) | Americas | Upper middle income | High |
| Japan | Men | Early Adulthood | 1.94 | 8.2 | 6.25 | 2 | 5.23 (5.22 to 5.25) | Western Pacific | High income | Very High |
| Japan | Men | Later Adulthood | 1.28 | 3.96 | 2.68 | 1 | 4.12 (4.09 to 4.15) | Western Pacific | High income | Very High |
| Japan | Women | Early Adulthood | 1.96 | 3.41 | 1.45 | 2 | 1.98 (1.97 to 1.99) | Western Pacific | High income | Very High |
| Japan | Women | Later Adulthood | 3.22 | 3.79 | 0.57 | 2 | 0.61 (0.60 to 0.62) | Western Pacific | High income | Very High |
| Jordan | Men | Early Adulthood | 12.49 | 28.96 | 16.47 | 2 | 3.06 (3.03 to 3.08) | Eastern Mediterranean | Lower middle income | High |
| Jordan | Men | Later Adulthood | 21.2 | 42 | 20.81 | 3 | 2.47 (2.45 to 2.49) | Eastern Mediterranean | Lower middle income | High |
| Jordan | Women | Early Adulthood | 25.18 | 33.47 | 8.29 | 2 | 1.03 (1.02 to 1.05) | Eastern Mediterranean | Lower middle income | High |
| Jordan | Women | Later Adulthood | 48.41 | 67.46 | 19.06 | 3 | 1.21 (1.20 to 1.22) | Eastern Mediterranean | Lower middle income | High |
| Kazakhstan | Men | Early Adulthood | 4.59 | 13.62 | 9.03 | 2 | 3.97 (3.95 to 3.98) | European | Upper middle income | Very High |
| Kazakhstan | Men | Later Adulthood | 9.01 | 26.16 | 17.15 | 1 | 3.84 (3.82 to 3.87) | European | Upper middle income | Very High |
| Kazakhstan | Women | Early Adulthood | 12.34 | 12.6 | 0.26 | 3 | 0.19 (0.17 to 0.20) | European | Upper middle income | Very High |
| Kazakhstan | Women | Later Adulthood | 27.61 | 33.35 | 5.74 | 3 | 0.69 (0.68 to 0.70) | European | Upper middle income | Very High |
| Kenya | Men | Early Adulthood | 0.89 | 4.06 | 3.17 | 1 | 5.56 (5.53 to 5.59) | African | Lower middle income | Medium |
| Kenya | Men | Later Adulthood | 2.04 | 8.45 | 6.41 | 1 | 5.21 (5.18 to 5.25) | African | Lower middle income | Medium |
| Kenya | Women | Early Adulthood | 2.64 | 15.46 | 12.82 | 2 | 6.49 (6.46 to 6.52) | African | Lower middle income | Medium |
| Kenya | Women | Later Adulthood | 5.07 | 20.76 | 15.7 | 3 | 5.11 (5.09 to 5.12) | African | Lower middle income | Medium |
| Kiribati | Men | Early Adulthood | 28.12 | 36.45 | 8.33 | 2 | 1.01 (0.99 to 1.03) | Western Pacific | Lower middle income | Medium |
| Kiribati | Men | Later Adulthood | 26.18 | 35.07 | 8.89 | 3 | 1.08 (1.07 to 1.10) | Western Pacific | Lower middle income | Medium |
| Kiribati | Women | Early Adulthood | 38.91 | 56.17 | 17.26 | 1 | 1.37 (1.35 to 1.39) | Western Pacific | Lower middle income | Medium |
| Kiribati | Women | Later Adulthood | 35.96 | 55.52 | 19.56 | 3 | 1.60 (1.59 to 1.61) | Western Pacific | Lower middle income | Medium |
| Kuwait | Men | Early Adulthood | 23.9 | 39.15 | 15.24 | 1 | 1.77 (1.69 to 1.86) | Eastern Mediterranean | High income | Very High |
| Kuwait | Men | Later Adulthood | 25.49 | 44.52 | 19.03 | 3 | 2.04 (2.02 to 2.05) | Eastern Mediterranean | High income | Very High |
| Kuwait | Women | Early Adulthood | 36.61 | 44.15 | 7.54 | 0 | 0.64 (0.57 to 0.71) | Eastern Mediterranean | High income | Very High |
| Kuwait | Women | Later Adulthood | 50.91 | 68.39 | 17.48 | 3 | 1.08 (1.07 to 1.08) | Eastern Mediterranean | High income | Very High |
| Kyrgyzstan | Men | Early Adulthood | 7.54 | 18.08 | 10.54 | 2 | 3.14 (3.09 to 3.18) | European | Lower middle income | High |
| Kyrgyzstan | Men | Later Adulthood | 10.05 | 33.28 | 23.23 | 3 | 4.27 (4.26 to 4.29) | European | Lower middle income | High |
| Kyrgyzstan | Women | Early Adulthood | 8.15 | 17.77 | 9.63 | 2 | 2.82 (2.79 to 2.86) | European | Lower middle income | High |
| Kyrgyzstan | Women | Later Adulthood | 14.51 | 48 | 33.48 | 2 | 4.33 (4.31 to 4.35) | European | Lower middle income | High |
| Lao PDR | Men | Early Adulthood | 0.73 | 5.1 | 4.36 | 1 | 7.17 (7.14 to 7.20) | Western Pacific | Lower middle income | Medium |
| Lao PDR | Men | Later Adulthood | 1.21 | 5.94 | 4.73 | 3 | 5.84 (5.82 to 5.86) | Western Pacific | Lower middle income | Medium |
| Lao PDR | Women | Early Adulthood | 1.85 | 8.68 | 6.83 | 1 | 5.68 (5.67 to 5.69) | Western Pacific | Lower middle income | Medium |
| Lao PDR | Women | Later Adulthood | 2.69 | 10.86 | 8.17 | 0 | 5.12 (5.10 to 5.14) | Western Pacific | Lower middle income | Medium |
| Latvia | Men | Early Adulthood | 9.43 | 22.2 | 12.76 | 1 | 3.11 (3.07 to 3.15) | European | High income | Very High |
| Latvia | Men | Later Adulthood | 17.03 | 32.56 | 15.53 | 3 | 2.29 (2.28 to 2.30) | European | High income | Very High |
| Latvia | Women | Early Adulthood | 12.62 | 16.73 | 4.11 | 1 | 1.00 (0.97 to 1.04) | European | High income | Very High |
| Latvia | Women | Later Adulthood | 32.72 | 42.39 | 9.66 | 3 | 0.89 (0.89 to 0.90) | European | High income | Very High |
| Lebanon | Men | Early Adulthood | 11.49 | 25.42 | 13.93 | 3 | 2.86 (2.84 to 2.88) | Eastern Mediterranean | Lower middle income | High |
| Lebanon | Men | Later Adulthood | 16.19 | 35.41 | 19.22 | 3 | 2.83 (2.81 to 2.84) | Eastern Mediterranean | Lower middle income | High |
| Lebanon | Women | Early Adulthood | 12.82 | 22.41 | 9.59 | 1 | 2.06 (2.03 to 2.08) | Eastern Mediterranean | Lower middle income | High |
| Lebanon | Women | Later Adulthood | 26.7 | 48.65 | 21.95 | 2 | 2.17 (2.16 to 2.18) | Eastern Mediterranean | Lower middle income | High |
| Lesotho | Men | Early Adulthood | 1.2 | 5.79 | 4.6 | 2 | 5.92 (5.89 to 5.95) | African | Lower middle income | Medium |
| Lesotho | Men | Later Adulthood | 2.54 | 10.85 | 8.31 | 3 | 5.34 (5.33 to 5.35) | African | Lower middle income | Medium |
| Lesotho | Women | Early Adulthood | 12.55 | 28.35 | 15.8 | 2 | 2.99 (2.98 to 3.00) | African | Lower middle income | Medium |
| Lesotho | Women | Later Adulthood | 16.84 | 36.62 | 19.78 | 3 | 2.77 (2.76 to 2.78) | African | Lower middle income | Medium |
| Liberia | Men | Early Adulthood | 1.32 | 11.15 | 9.84 | 2 | 8.01 (7.98 to 8.04) | African | Low income | Low |
| Liberia | Men | Later Adulthood | 1.99 | 11.5 | 9.51 | 1 | 6.44 (6.35 to 6.53) | African | Low income | Low |
| Liberia | Women | Early Adulthood | 2.95 | 18.41 | 15.46 | 2 | 6.78 (6.77 to 6.80) | African | Low income | Low |
| Liberia | Women | Later Adulthood | 5.06 | 22.03 | 16.96 | 3 | 5.42 (5.40 to 5.43) | African | Low income | Low |
| Libya | Men | Early Adulthood | 10.76 | 24.94 | 14.18 | 2 | 3.04 (3.02 to 3.06) | Eastern Mediterranean | Upper middle income | High |
| Libya | Men | Later Adulthood | 14.36 | 33.22 | 18.86 | 3 | 3.05 (3.03 to 3.06) | Eastern Mediterranean | Upper middle income | High |
| Libya | Women | Early Adulthood | 23.19 | 39.7 | 16.52 | 1 | 1.89 (1.88 to 1.91) | Eastern Mediterranean | Upper middle income | High |
| Libya | Women | Later Adulthood | 36.59 | 63.41 | 26.82 | 3 | 1.98 (1.97 to 1.98) | Eastern Mediterranean | Upper middle income | High |
| Lithuania | Men | Early Adulthood | 11.99 | 23.17 | 11.18 | 3 | 2.28 (2.26 to 2.31) | European | High income | Very High |
| Lithuania | Men | Later Adulthood | 21.06 | 36.14 | 15.08 | 2 | 1.88 (1.87 to 1.89) | European | High income | Very High |
| Lithuania | Women | Early Adulthood | 15.81 | 15.99 | 0.18 | 2 | -0.06 (-0.08 to -0.03) | European | High income | Very High |
| Lithuania | Women | Later Adulthood | 43.25 | 43.98 | 0.74 | 2 | 0.03 (0.02 to 0.04) | European | High income | Very High |
| Luxembourg | Men | Early Adulthood | 10.53 | 17.26 | 6.73 | 2 | 1.80 (1.77 to 1.82) | European | High income | Very High |
| Luxembourg | Men | Later Adulthood | 19.57 | 29.22 | 9.66 | 2 | 1.46 (1.44 to 1.47) | European | High income | Very High |
| Luxembourg | Women | Early Adulthood | 9.25 | 14.36 | 5.11 | 2 | 1.60 (1.58 to 1.62) | European | High income | Very High |
| Luxembourg | Women | Later Adulthood | 23.79 | 23.29 | -0.51 | 2 | -0.07 (-0.08 to -0.06) | European | High income | Very High |
| Madagascar | Men | Early Adulthood | 0.51 | 3.23 | 2.72 | 0 | 6.73 (6.65 to 6.80) | African | Low income | Low |
| Madagascar | Men | Later Adulthood | 0.89 | 5.13 | 4.24 | 3 | 6.45 (6.43 to 6.47) | African | Low income | Low |
| Madagascar | Women | Early Adulthood | 0.56 | 3.23 | 2.68 | 3 | 6.39 (6.38 to 6.40) | African | Low income | Low |
| Madagascar | Women | Later Adulthood | 1 | 4.59 | 3.59 | 3 | 5.50 (5.49 to 5.51) | African | Low income | Low |
| Malawi | Men | Early Adulthood | 0.5 | 1.89 | 1.4 | 2 | 5.01 (5.00 to 5.03) | African | Low income | Low |
| Malawi | Men | Later Adulthood | 0.99 | 4.25 | 3.26 | 3 | 5.46 (5.45 to 5.48) | African | Low income | Low |
| Malawi | Women | Early Adulthood | 1.41 | 8.74 | 7.33 | 2 | 6.75 (6.71 to 6.78) | African | Low income | Low |
| Malawi | Women | Later Adulthood | 2.43 | 13.92 | 11.49 | 2 | 6.41 (6.39 to 6.43) | African | Low income | Low |
| Malaysia | Men | Early Adulthood | 3.76 | 18.28 | 14.52 | 2 | 5.83 (5.81 to 5.85) | Western Pacific | Upper middle income | Very High |
| Malaysia | Men | Later Adulthood | 3.89 | 13.86 | 9.97 | 2 | 4.65 (4.63 to 4.67) | Western Pacific | Upper middle income | Very High |
| Malaysia | Women | Early Adulthood | 7.4 | 26.5 | 19.1 | 3 | 4.69 (4.68 to 4.70) | Western Pacific | Upper middle income | Very High |
| Malaysia | Women | Later Adulthood | 9.13 | 24.64 | 15.51 | 3 | 3.61 (3.60 to 3.63) | Western Pacific | Upper middle income | Very High |
| Maldives | Men | Early Adulthood | 1.51 | 11.53 | 10.01 | 2 | 7.56 (7.53 to 7.58) | South-East Asia | Upper middle income | High |
| Maldives | Men | Later Adulthood | 2.75 | 12.82 | 10.07 | 1 | 5.65 (5.63 to 5.67) | South-East Asia | Upper middle income | High |
| Maldives | Women | Early Adulthood | 4.86 | 21.73 | 16.87 | 3 | 5.50 (5.49 to 5.51) | South-East Asia | Upper middle income | High |
| Maldives | Women | Later Adulthood | 9.01 | 24.87 | 15.86 | 3 | 3.68 (3.67 to 3.70) | South-East Asia | Upper middle income | High |
| Mali | Men | Early Adulthood | 1.26 | 7.7 | 6.44 | 1 | 6.68 (6.65 to 6.71) | African | Low income | Low |
| Mali | Men | Later Adulthood | 2.3 | 11.81 | 9.51 | 1 | 6.01 (5.97 to 6.05) | African | Low income | Low |
| Mali | Women | Early Adulthood | 1.64 | 10.52 | 8.88 | 1 | 6.86 (6.82 to 6.91) | African | Low income | Low |
| Mali | Women | Later Adulthood | 2.33 | 14.48 | 12.16 | 1 | 6.77 (6.73 to 6.82) | African | Low income | Low |
| Malta | Men | Early Adulthood | 18.44 | 32.13 | 13.69 | 1 | 2.00 (1.98 to 2.02) | European | High income | Very High |
| Malta | Men | Later Adulthood | 27.04 | 42.46 | 15.43 | 1 | 1.62 (1.60 to 1.64) | European | High income | Very High |
| Malta | Women | Early Adulthood | 19.48 | 24.04 | 4.56 | 1 | 0.75 (0.73 to 0.77) | European | High income | Very High |
| Malta | Women | Later Adulthood | 43.98 | 41.96 | -2.01 | 3 | -0.18 (-0.18 to -0.18) | European | High income | Very High |
| Marshall Islands | Men | Early Adulthood | 27.53 | 38.2 | 10.66 | 3 | 1.21 (1.19 to 1.22) | Western Pacific | Upper middle income | High |
| Marshall Islands | Men | Later Adulthood | 29.76 | 38.25 | 8.49 | 2 | 0.91 (0.89 to 0.92) | Western Pacific | Upper middle income | High |
| Marshall Islands | Women | Early Adulthood | 40.19 | 55.29 | 15.1 | 3 | 1.16 (1.15 to 1.16) | Western Pacific | Upper middle income | High |
| Marshall Islands | Women | Later Adulthood | 43.11 | 58.14 | 15.03 | 2 | 1.08 (1.07 to 1.09) | Western Pacific | Upper middle income | High |
| Mauritania | Men | Early Adulthood | 1.29 | 6.65 | 5.36 | 1 | 6.05 (6.01 to 6.10) | African | Lower middle income | Medium |
| Mauritania | Men | Later Adulthood | 2.6 | 11.31 | 8.72 | 3 | 5.45 (5.43 to 5.47) | African | Lower middle income | Medium |
| Mauritania | Women | Early Adulthood | 7.63 | 30.53 | 22.91 | 1 | 5.09 (5.06 to 5.12) | African | Lower middle income | Medium |
| Mauritania | Women | Later Adulthood | 11.06 | 38.32 | 27.26 | 2 | 4.57 (4.56 to 4.59) | African | Lower middle income | Medium |
| Mauritius | Men | Early Adulthood | 4.67 | 12.81 | 8.14 | 2 | 3.77 (3.72 to 3.81) | African | Upper middle income | Very High |
| Mauritius | Men | Later Adulthood | 5.3 | 12.41 | 7.11 | 2 | 3.12 (3.09 to 3.15) | African | Upper middle income | Very High |
| Mauritius | Women | Early Adulthood | 11.65 | 25 | 13.34 | 3 | 2.88 (2.85 to 2.91) | African | Upper middle income | Very High |
| Mauritius | Women | Later Adulthood | 18.12 | 25.92 | 7.8 | 2 | 1.31 (1.30 to 1.32) | African | Upper middle income | Very High |
| Mexico | Men | Early Adulthood | 10.66 | 31.4 | 20.74 | 3 | 3.96 (3.95 to 3.97) | Americas | Upper middle income | High |
| Mexico | Men | Later Adulthood | 15.76 | 29.28 | 13.53 | 3 | 2.26 (2.25 to 2.27) | Americas | Upper middle income | High |
| Mexico | Women | Early Adulthood | 15.06 | 38.02 | 22.96 | 3 | 3.42 (3.40 to 3.44) | Americas | Upper middle income | High |
| Mexico | Women | Later Adulthood | 26.12 | 44.24 | 18.12 | 2 | 1.94 (1.92 to 1.96) | Americas | Upper middle income | High |
| Moldova | Men | Early Adulthood | 8.02 | 17.1 | 9.08 | 3 | 2.80 (2.78 to 2.83) | European | Upper middle income | High |
| Moldova | Men | Later Adulthood | 16.33 | 32.22 | 15.88 | 2 | 2.44 (2.41 to 2.47) | European | Upper middle income | High |
| Moldova | Women | Early Adulthood | 17.34 | 17.54 | 0.19 | 3 | 0.12 (0.10 to 0.15) | European | Upper middle income | High |
| Moldova | Women | Later Adulthood | 43.49 | 45.51 | 2.02 | 3 | 0.15 (0.14 to 0.16) | European | Upper middle income | High |
| Mongolia | Men | Early Adulthood | 3.88 | 17.94 | 14.06 | 3 | 5.48 (5.46 to 5.51) | Western Pacific | Upper middle income | High |
| Mongolia | Men | Later Adulthood | 7.64 | 27.32 | 19.68 | 2 | 4.56 (4.53 to 4.59) | Western Pacific | Upper middle income | High |
| Mongolia | Women | Early Adulthood | 7.54 | 20.44 | 12.9 | 3 | 3.57 (3.56 to 3.58) | Western Pacific | Upper middle income | High |
| Mongolia | Women | Later Adulthood | 15.05 | 38.25 | 23.2 | 2 | 3.35 (3.34 to 3.36) | Western Pacific | Upper middle income | High |
| Montenegro | Men | Early Adulthood | 5.58 | 16.36 | 10.79 | 3 | 3.99 (3.98 to 4.01) | European | Upper middle income | Very High |
| Montenegro | Men | Later Adulthood | 10.92 | 29.14 | 18.22 | 1 | 3.57 (3.56 to 3.58) | European | Upper middle income | Very High |
| Montenegro | Women | Early Adulthood | 7.07 | 9.84 | 2.77 | 2 | 1.20 (1.18 to 1.22) | European | Upper middle income | Very High |
| Montenegro | Women | Later Adulthood | 23.04 | 29.92 | 6.89 | 2 | 0.93 (0.92 to 0.93) | European | Upper middle income | Very High |
| Morocco | Men | Early Adulthood | 4.08 | 11.75 | 7.66 | 3 | 3.85 (3.84 to 3.87) | Eastern Mediterranean | Lower middle income | High |
| Morocco | Men | Later Adulthood | 5.83 | 16.35 | 10.52 | 2 | 3.74 (3.72 to 3.75) | Eastern Mediterranean | Lower middle income | High |
| Morocco | Women | Early Adulthood | 10.76 | 24.89 | 14.13 | 3 | 3.02 (3.01 to 3.03) | Eastern Mediterranean | Lower middle income | High |
| Morocco | Women | Later Adulthood | 15.34 | 40.57 | 25.23 | 3 | 3.48 (3.47 to 3.49) | Eastern Mediterranean | Lower middle income | High |
| Mozambique | Men | Early Adulthood | 0.77 | 4.33 | 3.57 | 3 | 6.33 (6.31 to 6.35) | African | Low income | Low |
| Mozambique | Men | Later Adulthood | 1.55 | 7.48 | 5.93 | 2 | 5.85 (5.84 to 5.86) | African | Low income | Low |
| Mozambique | Women | Early Adulthood | 2.13 | 10.22 | 8.08 | 1 | 5.77 (5.75 to 5.79) | African | Low income | Low |
| Mozambique | Women | Later Adulthood | 3.76 | 16.68 | 12.93 | 3 | 5.40 (5.38 to 5.42) | African | Low income | Low |
| Myanmar | Men | Early Adulthood | 0.85 | 4.64 | 3.78 | 1 | 6.22 (6.18 to 6.25) | South-East Asia | Lower middle income | Medium |
| Myanmar | Men | Later Adulthood | 1.42 | 6.33 | 4.92 | 1 | 5.50 (5.47 to 5.52) | South-East Asia | Lower middle income | Medium |
| Myanmar | Women | Early Adulthood | 3.04 | 8.59 | 5.55 | 3 | 3.81 (3.80 to 3.82) | South-East Asia | Lower middle income | Medium |
| Myanmar | Women | Later Adulthood | 3.61 | 10.82 | 7.21 | 3 | 3.99 (3.98 to 4.01) | South-East Asia | Lower middle income | Medium |
| Namibia | Men | Early Adulthood | 1.91 | 7.07 | 5.16 | 1 | 4.77 (4.72 to 4.82) | African | Lower middle income | Medium |
| Namibia | Men | Later Adulthood | 4.48 | 14.48 | 9.99 | 2 | 4.30 (4.27 to 4.32) | African | Lower middle income | Medium |
| Namibia | Women | Early Adulthood | 7.83 | 18.9 | 11.08 | 3 | 3.27 (3.25 to 3.29) | African | Lower middle income | Medium |
| Namibia | Women | Later Adulthood | 12.99 | 29.08 | 16.09 | 2 | 2.95 (2.93 to 2.97) | African | Lower middle income | Medium |
| Nauru | Men | Early Adulthood | 71.93 | 70.17 | -1.75 | 2 | -0.08 (-0.10 to -0.07) | Western Pacific | High income | High |
| Nauru | Men | Later Adulthood | 59.56 | 70.93 | 11.37 | 2 | 0.61 (0.59 to 0.64) | Western Pacific | High income | High |
| Nauru | Women | Early Adulthood | 70.41 | 72.22 | 1.82 | 2 | 0.12 (0.11 to 0.12) | Western Pacific | High income | High |
| Nauru | Women | Later Adulthood | 68.82 | 73.69 | 4.86 | 2 | 0.25 (0.24 to 0.26) | Western Pacific | High income | High |
| Nepal | Men | Early Adulthood | 0.49 | 4.18 | 3.69 | 1 | 7.94 (7.90 to 7.98) | South-East Asia | Lower middle income | Medium |
| Nepal | Men | Later Adulthood | 0.83 | 4.37 | 3.54 | 2 | 6.12 (6.12 to 6.13) | South-East Asia | Lower middle income | Medium |
| Nepal | Women | Early Adulthood | 0.47 | 7.22 | 6.74 | 2 | 10.18 (10.16 to 10.21) | South-East Asia | Lower middle income | Medium |
| Nepal | Women | Later Adulthood | 0.85 | 8.96 | 8.11 | 2 | 8.75 (8.73 to 8.78) | South-East Asia | Lower middle income | Medium |
| Netherlands | Men | Early Adulthood | 5.44 | 11.7 | 6.27 | 2 | 2.80 (2.77 to 2.83) | European | High income | Very High |
| Netherlands | Men | Later Adulthood | 10.14 | 21.5 | 11.36 | 2 | 2.74 (2.72 to 2.75) | European | High income | Very High |
| Netherlands | Women | Early Adulthood | 6.58 | 11.93 | 5.35 | 2 | 2.18 (2.16 to 2.20) | European | High income | Very High |
| Netherlands | Women | Later Adulthood | 20.27 | 22.05 | 1.78 | 2 | 0.30 (0.30 to 0.31) | European | High income | Very High |
| New Zealand | Men | Early Adulthood | 12.53 | 30.19 | 17.67 | 2 | 3.23 (3.20 to 3.26) | Western Pacific | High income | Very High |
| New Zealand | Men | Later Adulthood | 17.05 | 36.06 | 19 | 2 | 2.74 (2.72 to 2.76) | Western Pacific | High income | Very High |
| New Zealand | Women | Early Adulthood | 14.13 | 33.48 | 19.35 | 3 | 3.20 (3.18 to 3.21) | Western Pacific | High income | Very High |
| New Zealand | Women | Later Adulthood | 19.6 | 36.35 | 16.75 | 3 | 2.26 (2.24 to 2.27) | Western Pacific | High income | Very High |
| Nicaragua | Men | Early Adulthood | 9.04 | 26.02 | 16.97 | 3 | 3.85 (3.84 to 3.86) | Americas | Lower middle income | High |
| Nicaragua | Men | Later Adulthood | 14.12 | 29.74 | 15.62 | 3 | 2.73 (2.71 to 2.74) | Americas | Lower middle income | High |
| Nicaragua | Women | Early Adulthood | 12.66 | 33.85 | 21.19 | 3 | 3.60 (3.59 to 3.61) | Americas | Lower middle income | High |
| Nicaragua | Women | Later Adulthood | 25.46 | 47.39 | 21.93 | 3 | 2.26 (2.25 to 2.28) | Americas | Lower middle income | High |
| Niger | Men | Early Adulthood | 0.42 | 3.5 | 3.07 | 1 | 7.83 (7.76 to 7.90) | African | Low income | Low |
| Niger | Men | Later Adulthood | 0.74 | 4.72 | 3.97 | 1 | 6.81 (6.75 to 6.86) | African | Low income | Low |
| Niger | Women | Early Adulthood | 1.41 | 6.38 | 4.97 | 1 | 5.55 (5.51 to 5.59) | African | Low income | Low |
| Niger | Women | Later Adulthood | 1.87 | 8.14 | 6.27 | 1 | 5.39 (5.36 to 5.43) | African | Low income | Low |
| Nigeria | Men | Early Adulthood | 0.98 | 6.11 | 5.12 | 2 | 6.75 (6.72 to 6.78) | African | Lower middle income | Medium |
| Nigeria | Men | Later Adulthood | 1.59 | 10.7 | 9.1 | 2 | 7.07 (7.03 to 7.11) | African | Lower middle income | Medium |
| Nigeria | Women | Early Adulthood | 3.87 | 13.04 | 9.17 | 3 | 4.49 (4.47 to 4.50) | African | Lower middle income | Medium |
| Nigeria | Women | Later Adulthood | 4.59 | 20.34 | 15.75 | 3 | 5.52 (5.51 to 5.54) | African | Lower middle income | Medium |
| North Korea | Men | Early Adulthood | 1.17 | 8.36 | 7.19 | 1 | 7.30 (7.26 to 7.35) | South-East Asia | Low income | Unclassified |
| North Korea | Men | Later Adulthood | 1.56 | 6.86 | 5.3 | 3 | 5.45 (5.45 to 5.46) | South-East Asia | Low income | Unclassified |
| North Korea | Women | Early Adulthood | 3.02 | 12.52 | 9.5 | 2 | 5.21 (5.19 to 5.24) | South-East Asia | Low income | Unclassified |
| North Korea | Women | Later Adulthood | 3.85 | 12.19 | 8.34 | 2 | 4.25 (4.23 to 4.27) | South-East Asia | Low income | Unclassified |
| North Macedonia | Men | Early Adulthood | 9.11 | 23.91 | 14.81 | 2 | 3.53 (3.51 to 3.55) | European | Upper middle income | Very High |
| North Macedonia | Men | Later Adulthood | 16.52 | 36.51 | 19.99 | 2 | 2.84 (2.84 to 2.85) | European | Upper middle income | Very High |
| North Macedonia | Women | Early Adulthood | 12.57 | 20.45 | 7.87 | 1 | 1.73 (1.71 to 1.75) | European | Upper middle income | Very High |
| North Macedonia | Women | Later Adulthood | 32.38 | 42.8 | 10.42 | 3 | 0.98 (0.97 to 0.98) | European | Upper middle income | Very High |
| Norway | Men | Early Adulthood | 7.02 | 19.33 | 12.31 | 2 | 3.70 (3.68 to 3.71) | European | High income | Very High |
| Norway | Men | Later Adulthood | 10.62 | 22.29 | 11.68 | 2 | 2.69 (2.68 to 2.70) | European | High income | Very High |
| Norway | Women | Early Adulthood | 6.37 | 16.47 | 10.1 | 2 | 3.47 (3.46 to 3.48) | European | High income | Very High |
| Norway | Women | Later Adulthood | 16.93 | 20.23 | 3.3 | 2 | 0.64 (0.62 to 0.65) | European | High income | Very High |
| Oman | Men | Early Adulthood | 10.57 | 24.07 | 13.5 | 2 | 2.99 (2.95 to 3.02) | Eastern Mediterranean | High income | Very High |
| Oman | Men | Later Adulthood | 11.29 | 29.99 | 18.7 | 3 | 3.55 (3.53 to 3.57) | Eastern Mediterranean | High income | Very High |
| Oman | Women | Early Adulthood | 21.71 | 35.48 | 13.77 | 3 | 1.77 (1.76 to 1.78) | Eastern Mediterranean | High income | Very High |
| Oman | Women | Later Adulthood | 21.72 | 47.92 | 26.2 | 3 | 2.84 (2.83 to 2.85) | Eastern Mediterranean | High income | Very High |
| Pakistan | Men | Early Adulthood | 1.58 | 17.11 | 15.54 | 3 | 8.78 (8.76 to 8.80) | Eastern Mediterranean | Lower middle income | Low |
| Pakistan | Men | Later Adulthood | 2.31 | 19.03 | 16.72 | 3 | 7.71 (7.69 to 7.73) | Eastern Mediterranean | Lower middle income | Low |
| Pakistan | Women | Early Adulthood | 4.76 | 22.58 | 17.82 | 1 | 5.75 (5.72 to 5.78) | Eastern Mediterranean | Lower middle income | Low |
| Pakistan | Women | Later Adulthood | 6.25 | 27.05 | 20.8 | 3 | 5.35 (5.34 to 5.35) | Eastern Mediterranean | Lower middle income | Low |
| Palau | Men | Early Adulthood | 32.61 | 41.46 | 8.85 | 2 | 0.89 (0.87 to 0.92) | Western Pacific | High income | High |
| Palau | Men | Later Adulthood | 30.94 | 38.24 | 7.31 | 3 | 0.78 (0.77 to 0.79) | Western Pacific | High income | High |
| Palau | Women | Early Adulthood | 36.67 | 45.91 | 9.24 | 2 | 0.84 (0.82 to 0.85) | Western Pacific | High income | High |
| Palau | Women | Later Adulthood | 38.16 | 46.39 | 8.23 | 2 | 0.70 (0.69 to 0.70) | Western Pacific | High income | High |
| Panama | Men | Early Adulthood | 8.86 | 27.96 | 19.1 | 3 | 4.18 (4.18 to 4.19) | Americas | High income | Very High |
| Panama | Men | Later Adulthood | 10.52 | 28.8 | 18.28 | 3 | 3.67 (3.66 to 3.67) | Americas | High income | Very High |
| Panama | Women | Early Adulthood | 12.47 | 41.76 | 29.29 | 3 | 4.42 (4.40 to 4.43) | Americas | High income | Very High |
| Panama | Women | Later Adulthood | 19.64 | 42.35 | 22.71 | 3 | 2.78 (2.77 to 2.79) | Americas | High income | Very High |
| Papua New Guinea | Men | Early Adulthood | 10.95 | 14.47 | 3.53 | 2 | 1.05 (1.04 to 1.06) | Western Pacific | Lower middle income | Medium |
| Papua New Guinea | Men | Later Adulthood | 10.65 | 18.63 | 7.98 | 2 | 2.03 (2.02 to 2.04) | Western Pacific | Lower middle income | Medium |
| Papua New Guinea | Women | Early Adulthood | 14.95 | 25.22 | 10.27 | 2 | 1.90 (1.89 to 1.90) | Western Pacific | Lower middle income | Medium |
| Papua New Guinea | Women | Later Adulthood | 13.45 | 25.29 | 11.83 | 1 | 2.28 (2.28 to 2.29) | Western Pacific | Lower middle income | Medium |
| Paraguay | Men | Early Adulthood | 8.42 | 26.28 | 17.86 | 2 | 4.20 (4.18 to 4.22) | Americas | Upper middle income | High |
| Paraguay | Men | Later Adulthood | 14.6 | 33.38 | 18.78 | 1 | 3.02 (3.01 to 3.03) | Americas | Upper middle income | High |
| Paraguay | Women | Early Adulthood | 11.26 | 30.58 | 19.33 | 2 | 3.66 (3.66 to 3.67) | Americas | Upper middle income | High |
| Paraguay | Women | Later Adulthood | 23.85 | 43.29 | 19.43 | 2 | 2.15 (2.15 to 2.16) | Americas | Upper middle income | High |
| Peru | Men | Early Adulthood | 4.16 | 20.89 | 16.74 | 1 | 5.91 (5.90 to 5.92) | Americas | Upper middle income | High |
| Peru | Men | Later Adulthood | 8.39 | 22.93 | 14.54 | 2 | 3.67 (3.67 to 3.67) | Americas | Upper middle income | High |
| Peru | Women | Early Adulthood | 9.36 | 28.31 | 18.94 | 2 | 4.05 (4.03 to 4.06) | Americas | Upper middle income | High |
| Peru | Women | Later Adulthood | 20 | 33.84 | 13.84 | 3 | 1.92 (1.92 to 1.93) | Americas | Upper middle income | High |
| Philippines | Men | Early Adulthood | 1.2 | 7.25 | 6.05 | 3 | 6.67 (6.65 to 6.68) | Western Pacific | Lower middle income | High |
| Philippines | Men | Later Adulthood | 1.27 | 5.67 | 4.4 | 1 | 5.51 (5.48 to 5.54) | Western Pacific | Lower middle income | High |
| Philippines | Women | Early Adulthood | 2.62 | 9.68 | 7.06 | 3 | 4.97 (4.95 to 5.00) | Western Pacific | Lower middle income | High |
| Philippines | Women | Later Adulthood | 3.1 | 10.15 | 7.05 | 3 | 4.46 (4.43 to 4.49) | Western Pacific | Lower middle income | High |
| Poland | Men | Early Adulthood | 9.72 | 27.44 | 17.73 | 3 | 3.84 (3.84 to 3.85) | European | High income | Very High |
| Poland | Men | Later Adulthood | 18.06 | 39.87 | 21.8 | 1 | 2.88 (2.86 to 2.89) | European | High income | Very High |
| Poland | Women | Early Adulthood | 10.97 | 17.11 | 6.14 | 3 | 1.66 (1.65 to 1.66) | European | High income | Very High |
| Poland | Women | Later Adulthood | 30.53 | 41.19 | 10.66 | 2 | 1.06 (1.05 to 1.06) | European | High income | Very High |
| Portugal | Men | Early Adulthood | 6.46 | 17.54 | 11.07 | 2 | 3.64 (3.62 to 3.66) | European | High income | Very High |
| Portugal | Men | Later Adulthood | 12.67 | 31.44 | 18.77 | 2 | 3.32 (3.31 to 3.34) | European | High income | Very High |
| Portugal | Women | Early Adulthood | 10.2 | 17.12 | 6.92 | 3 | 1.79 (1.78 to 1.80) | European | High income | Very High |
| Portugal | Women | Later Adulthood | 28.48 | 37.44 | 8.96 | 3 | 0.96 (0.95 to 0.96) | European | High income | Very High |
| Puerto Rico | Men | Early Adulthood | 16.24 | 36.71 | 20.47 | 3 | 2.99 (2.97 to 3.01) | Americas | High income | Very High |
| Puerto Rico | Men | Later Adulthood | 15.96 | 31.54 | 15.58 | 3 | 2.49 (2.48 to 2.50) | Americas | High income | Very High |
| Puerto Rico | Women | Early Adulthood | 20.62 | 44.8 | 24.18 | 2 | 2.83 (2.81 to 2.85) | Americas | High income | Very High |
| Puerto Rico | Women | Later Adulthood | 26.03 | 46.57 | 20.54 | 1 | 2.13 (2.10 to 2.15) | Americas | High income | Very High |
| Qatar | Men | Early Adulthood | 17.14 | 39.73 | 22.59 | 3 | 3.08 (3.05 to 3.12) | Eastern Mediterranean | High income | Very High |
| Qatar | Men | Later Adulthood | 19.45 | 42.21 | 22.76 | 3 | 2.79 (2.78 to 2.80) | Eastern Mediterranean | High income | Very High |
| Qatar | Women | Early Adulthood | 26.21 | 44.99 | 18.78 | 2 | 1.98 (1.93 to 2.03) | Eastern Mediterranean | High income | Very High |
| Qatar | Women | Later Adulthood | 36.18 | 66.23 | 30.05 | 2 | 2.17 (2.16 to 2.18) | Eastern Mediterranean | High income | Very High |
| Romania | Men | Early Adulthood | 5.64 | 32.91 | 27.27 | 2 | 6.26 (6.22 to 6.31) | European | High income | Very High |
| Romania | Men | Later Adulthood | 10.12 | 43.43 | 33.31 | 3 | 5.17 (5.14 to 5.20) | European | High income | Very High |
| Romania | Women | Early Adulthood | 7.18 | 24.05 | 16.86 | 3 | 4.23 (4.18 to 4.28) | European | High income | Very High |
| Romania | Women | Later Adulthood | 18.75 | 45.81 | 27.06 | 2 | 3.14 (3.12 to 3.17) | European | High income | Very High |
| Russian Federation | Men | Early Adulthood | 6.77 | 21.47 | 14.7 | 1 | 4.21 (4.18 to 4.23) | European | High income | Very High |
| Russian Federation | Men | Later Adulthood | 14.36 | 29.52 | 15.16 | 2 | 2.51 (2.49 to 2.53) | European | High income | Very High |
| Russian Federation | Women | Early Adulthood | 17.4 | 18.56 | 1.15 | 3 | 0.32 (0.30 to 0.34) | European | High income | Very High |
| Russian Federation | Women | Later Adulthood | 38.11 | 43.06 | 4.95 | 2 | 0.43 (0.42 to 0.44) | European | High income | Very High |
| Rwanda | Men | Early Adulthood | 0.18 | 1.03 | 0.85 | 1 | 6.49 (6.44 to 6.54) | African | Low income | Medium |
| Rwanda | Men | Later Adulthood | 0.41 | 2.4 | 1.99 | 3 | 6.56 (6.53 to 6.59) | African | Low income | Medium |
| Rwanda | Women | Early Adulthood | 0.85 | 6.9 | 6.04 | 1 | 7.69 (7.65 to 7.72) | African | Low income | Medium |
| Rwanda | Women | Later Adulthood | 1.16 | 6.88 | 5.72 | 2 | 6.44 (6.41 to 6.47) | African | Low income | Medium |
| Saint Kitts and Nevis | Men | Early Adulthood | 14.25 | 36.27 | 22.02 | 3 | 3.45 (3.43 to 3.47) | Americas | High income | Very High |
| Saint Kitts and Nevis | Men | Later Adulthood | 14.46 | 37.86 | 23.4 | 3 | 3.50 (3.49 to 3.52) | Americas | High income | Very High |
| Saint Kitts and Nevis | Women | Early Adulthood | 24.88 | 52.43 | 27.55 | 2 | 2.73 (2.71 to 2.75) | Americas | High income | Very High |
| Saint Kitts and Nevis | Women | Later Adulthood | 29.93 | 57.4 | 27.46 | 3 | 2.33 (2.32 to 2.34) | Americas | High income | Very High |
| Saint Lucia | Men | Early Adulthood | 4.85 | 20.62 | 15.78 | 2 | 5.34 (5.31 to 5.36) | Americas | Upper middle income | High |
| Saint Lucia | Men | Later Adulthood | 5.09 | 17.72 | 12.63 | 2 | 4.57 (4.56 to 4.58) | Americas | Upper middle income | High |
| Saint Lucia | Women | Early Adulthood | 15.88 | 45.38 | 29.5 | 2 | 3.84 (3.83 to 3.86) | Americas | Upper middle income | High |
| Saint Lucia | Women | Later Adulthood | 20.06 | 46.19 | 26.13 | 3 | 3.02 (3.01 to 3.03) | Americas | Upper middle income | High |
| Saint Vincent and the Grenadines | Men | Early Adulthood | 5.63 | 18.13 | 12.51 | 2 | 4.29 (4.28 to 4.31) | Americas | Upper middle income | High |
| Saint Vincent and the Grenadines | Men | Later Adulthood | 6.3 | 18.61 | 12.3 | 3 | 3.96 (3.95 to 3.97) | Americas | Upper middle income | High |
| Saint Vincent and the Grenadines | Women | Early Adulthood | 19.09 | 47.57 | 28.49 | 3 | 3.33 (3.32 to 3.34) | Americas | Upper middle income | High |
| Saint Vincent and the Grenadines | Women | Later Adulthood | 23.87 | 48.4 | 24.53 | 3 | 2.54 (2.53 to 2.54) | Americas | Upper middle income | High |
| Samoa | Men | Early Adulthood | 24.45 | 47.83 | 23.38 | 2 | 2.45 (2.44 to 2.46) | Western Pacific | Upper middle income | High |
| Samoa | Men | Later Adulthood | 36.9 | 58.6 | 21.7 | 3 | 1.69 (1.68 to 1.69) | Western Pacific | Upper middle income | High |
| Samoa | Women | Early Adulthood | 46.7 | 72.78 | 26.08 | 2 | 1.61 (1.60 to 1.61) | Western Pacific | Upper middle income | High |
| Samoa | Women | Later Adulthood | 56.73 | 74.35 | 17.62 | 3 | 0.97 (0.97 to 0.98) | Western Pacific | Upper middle income | High |
| Sao Tome and Principe | Men | Early Adulthood | 1.3 | 7.27 | 5.97 | 2 | 6.40 (6.37 to 6.42) | African | Lower middle income | Medium |
| Sao Tome and Principe | Men | Later Adulthood | 2.61 | 12.76 | 10.14 | 1 | 5.83 (5.80 to 5.86) | African | Lower middle income | Medium |
| Sao Tome and Principe | Women | Early Adulthood | 4.43 | 19.33 | 14.9 | 2 | 5.44 (5.41 to 5.47) | African | Lower middle income | Medium |
| Sao Tome and Principe | Women | Later Adulthood | 7.14 | 27.55 | 20.4 | 2 | 4.96 (4.95 to 4.97) | African | Lower middle income | Medium |
| Saudi Arabia | Men | Early Adulthood | 15.87 | 35.75 | 19.88 | 1 | 2.92 (2.85 to 3.00) | Eastern Mediterranean | High income | Very High |
| Saudi Arabia | Men | Later Adulthood | 18.91 | 41.62 | 22.71 | 3 | 2.83 (2.82 to 2.84) | Eastern Mediterranean | High income | Very High |
| Saudi Arabia | Women | Early Adulthood | 28.21 | 38.71 | 10.5 | 2 | 1.19 (1.17 to 1.21) | Eastern Mediterranean | High income | Very High |
| Saudi Arabia | Women | Later Adulthood | 34.01 | 63.04 | 29.03 | 3 | 2.23 (2.23 to 2.24) | Eastern Mediterranean | High income | Very High |
| Senegal | Men | Early Adulthood | 0.42 | 2.96 | 2.53 | 1 | 7.21 (7.16 to 7.25) | African | Lower middle income | Low |
| Senegal | Men | Later Adulthood | 0.85 | 4.61 | 3.77 | 1 | 6.24 (6.19 to 6.29) | African | Lower middle income | Low |
| Senegal | Women | Early Adulthood | 3.5 | 11.33 | 7.83 | 2 | 4.33 (4.31 to 4.35) | African | Lower middle income | Low |
| Senegal | Women | Later Adulthood | 6.12 | 21.06 | 14.94 | 3 | 4.60 (4.57 to 4.63) | African | Lower middle income | Low |
| Serbia | Men | Early Adulthood | 8.97 | 22.68 | 13.72 | 3 | 3.39 (3.38 to 3.40) | European | Upper middle income | Very High |
| Serbia | Men | Later Adulthood | 14.46 | 31.18 | 16.72 | 3 | 2.75 (2.74 to 2.76) | European | Upper middle income | Very High |
| Serbia | Women | Early Adulthood | 9.94 | 14.2 | 4.26 | 3 | 1.31 (1.31 to 1.32) | European | Upper middle income | Very High |
| Serbia | Women | Later Adulthood | 30.58 | 33.1 | 2.52 | 2 | 0.28 (0.27 to 0.28) | European | Upper middle income | Very High |
| Seychelles | Men | Early Adulthood | 4.98 | 19.4 | 14.42 | 2 | 5.05 (5.00 to 5.10) | African | High income | Very High |
| Seychelles | Men | Later Adulthood | 6.84 | 20.97 | 14.13 | 2 | 4.11 (4.07 to 4.14) | African | High income | Very High |
| Seychelles | Women | Early Adulthood | 16.99 | 37.52 | 20.53 | 2 | 2.89 (2.86 to 2.92) | African | High income | Very High |
| Seychelles | Women | Later Adulthood | 31.85 | 45.94 | 14.09 | 3 | 1.33 (1.32 to 1.33) | African | High income | Very High |
| Sierra Leone | Men | Early Adulthood | 0.57 | 2.1 | 1.52 | 1 | 4.75 (4.64 to 4.86) | African | Low income | Low |
| Sierra Leone | Men | Later Adulthood | 0.9 | 3.51 | 2.61 | 1 | 4.99 (4.91 to 5.06) | African | Low income | Low |
| Sierra Leone | Women | Early Adulthood | 3.02 | 9.96 | 6.94 | 1 | 4.35 (4.30 to 4.40) | African | Low income | Low |
| Sierra Leone | Women | Later Adulthood | 3.69 | 12.89 | 9.2 | 2 | 4.60 (4.58 to 4.63) | African | Low income | Low |
| Singapore | Men | Early Adulthood | 2.76 | 16.24 | 13.48 | 1 | 6.45 (6.42 to 6.47) | Western Pacific | High income | Very High |
| Singapore | Men | Later Adulthood | 2.68 | 10.02 | 7.33 | 2 | 4.70 (4.66 to 4.74) | Western Pacific | High income | Very High |
| Singapore | Women | Early Adulthood | 4.31 | 10.65 | 6.34 | 3 | 3.24 (3.22 to 3.26) | Western Pacific | High income | Very High |
| Singapore | Women | Later Adulthood | 8.89 | 11.01 | 2.12 | 3 | 0.70 (0.68 to 0.71) | Western Pacific | High income | Very High |
| Slovakia | Men | Early Adulthood | 9.56 | 26.56 | 17 | 3 | 3.72 (3.72 to 3.73) | European | High income | Very High |
| Slovakia | Men | Later Adulthood | 19.07 | 39.38 | 20.31 | 3 | 2.58 (2.58 to 2.59) | European | High income | Very High |
| Slovakia | Women | Early Adulthood | 9.2 | 17.07 | 7.87 | 3 | 2.23 (2.22 to 2.23) | European | High income | Very High |
| Slovakia | Women | Later Adulthood | 25.97 | 38.09 | 12.12 | 3 | 1.34 (1.33 to 1.35) | European | High income | Very High |
| Slovenia | Men | Early Adulthood | 9.74 | 22.44 | 12.7 | 2 | 3.12 (3.10 to 3.14) | European | High income | Very High |
| Slovenia | Men | Later Adulthood | 17.99 | 30.12 | 12.13 | 2 | 1.94 (1.93 to 1.96) | European | High income | Very High |
| Slovenia | Women | Early Adulthood | 7.62 | 10.7 | 3.07 | 2 | 1.25 (1.24 to 1.27) | European | High income | Very High |
| Slovenia | Women | Later Adulthood | 23.09 | 22.35 | -0.73 | 1 | -0.09 (-0.11 to -0.08) | European | High income | Very High |
| Solomon Islands | Men | Early Adulthood | 9.68 | 16.06 | 6.38 | 2 | 1.79 (1.78 to 1.80) | Western Pacific | Lower middle income | Medium |
| Solomon Islands | Men | Later Adulthood | 12.27 | 19.93 | 7.66 | 1 | 1.75 (1.73 to 1.77) | Western Pacific | Lower middle income | Medium |
| Solomon Islands | Women | Early Adulthood | 15.83 | 26.64 | 10.81 | 2 | 1.84 (1.83 to 1.85) | Western Pacific | Lower middle income | Medium |
| Solomon Islands | Women | Later Adulthood | 17.92 | 29.05 | 11.13 | 0 | 1.74 (1.73 to 1.75) | Western Pacific | Lower middle income | Medium |
| Somalia | Men | Early Adulthood | 0.81 | 4.13 | 3.32 | 3 | 6.09 (6.08 to 6.10) | Eastern Mediterranean | Low income | Low |
| Somalia | Men | Later Adulthood | 1.57 | 7.04 | 5.47 | 3 | 5.56 (5.55 to 5.58) | Eastern Mediterranean | Low income | Low |
| Somalia | Women | Early Adulthood | 4.55 | 18.53 | 13.98 | 2 | 5.12 (5.11 to 5.14) | Eastern Mediterranean | Low income | Low |
| Somalia | Women | Later Adulthood | 7.88 | 26.51 | 18.63 | 3 | 4.38 (4.36 to 4.39) | Eastern Mediterranean | Low income | Low |
| South Africa | Men | Early Adulthood | 5.16 | 10.65 | 5.49 | 2 | 2.71 (2.66 to 2.76) | African | Upper middle income | High |
| South Africa | Men | Later Adulthood | 9.27 | 21.54 | 12.27 | 2 | 3.10 (3.06 to 3.14) | African | Upper middle income | High |
| South Africa | Women | Early Adulthood | 22.48 | 41.6 | 19.12 | 1 | 2.24 (2.23 to 2.25) | African | Upper middle income | High |
| South Africa | Women | Later Adulthood | 34.57 | 54.24 | 19.67 | 3 | 1.63 (1.63 to 1.64) | African | Upper middle income | High |
| South Korea | Men | Early Adulthood | 1.11 | 9.33 | 8.23 | 3 | 7.93 (7.92 to 7.94) | Western Pacific | High income | Very High |
| South Korea | Men | Later Adulthood | 0.86 | 3.78 | 2.92 | 3 | 5.42 (5.41 to 5.43) | Western Pacific | High income | Very High |
| South Korea | Women | Early Adulthood | 1.81 | 5.09 | 3.29 | 2 | 3.82 (3.82 to 3.83) | Western Pacific | High income | Very High |
| South Korea | Women | Later Adulthood | 3.26 | 5.7 | 2.44 | 3 | 2.05 (2.04 to 2.06) | Western Pacific | High income | Very High |
| South Sudan | Men | Early Adulthood | 0.73 | 3.98 | 3.24 | 2 | 6.17 (6.11 to 6.23) | African | Low income | Low |
| South Sudan | Men | Later Adulthood | 1.48 | 6.27 | 4.78 | 2 | 5.33 (5.32 to 5.34) | African | Low income | Low |
| South Sudan | Women | Early Adulthood | 2.11 | 10.77 | 8.66 | 2 | 5.90 (5.80 to 5.99) | African | Low income | Low |
| South Sudan | Women | Later Adulthood | 3.52 | 12.52 | 9 | 1 | 4.66 (4.63 to 4.68) | African | Low income | Low |
| Spain | Men | Early Adulthood | 10.75 | 17.18 | 6.43 | 2 | 1.67 (1.64 to 1.70) | European | High income | Very High |
| Spain | Men | Later Adulthood | 18.08 | 28.04 | 9.96 | 2 | 1.59 (1.56 to 1.61) | European | High income | Very High |
| Spain | Women | Early Adulthood | 10.51 | 9.64 | -0.87 | 2 | -0.33 (-0.35 to -0.30) | European | High income | Very High |
| Spain | Women | Later Adulthood | 32.57 | 25.13 | -7.43 | 3 | -0.93 (-0.94 to -0.92) | European | High income | Very High |
| Sri Lanka | Men | Early Adulthood | 0.63 | 6.1 | 5.46 | 2 | 8.42 (8.39 to 8.45) | South-East Asia | Lower middle income | High |
| Sri Lanka | Men | Later Adulthood | 0.73 | 5.84 | 5.11 | 1 | 7.75 (7.71 to 7.78) | South-East Asia | Lower middle income | High |
| Sri Lanka | Women | Early Adulthood | 1.97 | 13.71 | 11.74 | 3 | 7.16 (7.14 to 7.17) | South-East Asia | Lower middle income | High |
| Sri Lanka | Women | Later Adulthood | 1.93 | 12.37 | 10.45 | 3 | 6.86 (6.84 to 6.87) | South-East Asia | Lower middle income | High |
| Sudan | Men | Early Adulthood | 1.58 | 8.61 | 7.04 | 3 | 6.33 (6.31 to 6.34) | Eastern Mediterranean | Low income | Low |
| Sudan | Men | Later Adulthood | 2.74 | 12.53 | 9.79 | 3 | 5.59 (5.58 to 5.61) | Eastern Mediterranean | Low income | Low |
| Sudan | Women | Early Adulthood | 5.43 | 18.35 | 12.91 | 2 | 4.45 (4.44 to 4.46) | Eastern Mediterranean | Low income | Low |
| Sudan | Women | Later Adulthood | 9.78 | 26.83 | 17.04 | 3 | 3.62 (3.61 to 3.64) | Eastern Mediterranean | Low income | Low |
| Suriname | Men | Early Adulthood | 5.4 | 18.49 | 13.09 | 2 | 4.53 (4.51 to 4.55) | Americas | Upper middle income | High |
| Suriname | Men | Later Adulthood | 6.61 | 19.12 | 12.51 | 3 | 3.88 (3.87 to 3.89) | Americas | Upper middle income | High |
| Suriname | Women | Early Adulthood | 13.76 | 35.31 | 21.55 | 3 | 3.44 (3.43 to 3.46) | Americas | Upper middle income | High |
| Suriname | Women | Later Adulthood | 21.38 | 43.68 | 22.3 | 3 | 2.59 (2.58 to 2.60) | Americas | Upper middle income | High |
| Sweden | Men | Early Adulthood | 8.01 | 15.36 | 7.35 | 1 | 2.35 (2.32 to 2.38) | European | High income | Very High |
| Sweden | Men | Later Adulthood | 11.4 | 19.91 | 8.51 | 2 | 2.02 (2.01 to 2.04) | European | High income | Very High |
| Sweden | Women | Early Adulthood | 7.7 | 12.29 | 4.59 | 2 | 1.71 (1.70 to 1.72) | European | High income | Very High |
| Sweden | Women | Later Adulthood | 15.44 | 18.38 | 2.94 | 2 | 0.63 (0.62 to 0.64) | European | High income | Very High |
| Switzerland | Men | Early Adulthood | 7.92 | 12.19 | 4.27 | 1 | 1.54 (1.51 to 1.56) | European | High income | Very High |
| Switzerland | Men | Later Adulthood | 16.55 | 21.32 | 4.77 | 1 | 0.90 (0.88 to 0.92) | European | High income | Very High |
| Switzerland | Women | Early Adulthood | 5.42 | 8.3 | 2.88 | 3 | 1.58 (1.56 to 1.59) | European | High income | Very High |
| Switzerland | Women | Later Adulthood | 16.51 | 13.74 | -2.78 | 1 | -0.65 (-0.66 to -0.64) | European | High income | Very High |
| Syrian Arab Republic | Men | Early Adulthood | 9.59 | 22.65 | 13.06 | 2 | 3.04 (3.03 to 3.05) | Eastern Mediterranean | Low income | Medium |
| Syrian Arab Republic | Men | Later Adulthood | 13.43 | 30.85 | 17.42 | 3 | 3.01 (3.00 to 3.02) | Eastern Mediterranean | Low income | Medium |
| Syrian Arab Republic | Women | Early Adulthood | 20.41 | 32.86 | 12.44 | 3 | 1.61 (1.59 to 1.63) | Eastern Mediterranean | Low income | Medium |
| Syrian Arab Republic | Women | Later Adulthood | 33.06 | 57.53 | 24.46 | 3 | 1.99 (1.99 to 2.00) | Eastern Mediterranean | Low income | Medium |
| Taiwan | Men | Early Adulthood | 2.13 | 13.41 | 11.28 | 2 | 6.83 (6.81 to 6.85) | Western Pacific | High income | Very High |
| Taiwan | Men | Later Adulthood | 2.46 | 6.84 | 4.38 | 1 | 3.73 (3.71 to 3.74) | Western Pacific | High income | Very High |
| Taiwan | Women | Early Adulthood | 3.68 | 9.1 | 5.42 | 3 | 3.36 (3.34 to 3.37) | Western Pacific | High income | Very High |
| Taiwan | Women | Later Adulthood | 6.45 | 10.84 | 4.4 | 3 | 1.91 (1.91 to 1.92) | Western Pacific | High income | Very High |
| Tajikistan | Men | Early Adulthood | 5.01 | 14.86 | 9.85 | 3 | 3.94 (3.93 to 3.96) | European | Lower middle income | Medium |
| Tajikistan | Men | Later Adulthood | 9.23 | 26.27 | 17.03 | 2 | 3.74 (3.72 to 3.75) | European | Lower middle income | Medium |
| Tajikistan | Women | Early Adulthood | 5.23 | 16.08 | 10.85 | 1 | 4.09 (4.07 to 4.11) | European | Lower middle income | Medium |
| Tajikistan | Women | Later Adulthood | 15.45 | 45.54 | 30.1 | 3 | 3.87 (3.86 to 3.88) | European | Lower middle income | Medium |
| Tanzania | Men | Early Adulthood | 0.95 | 4.67 | 3.72 | 3 | 5.85 (5.83 to 5.86) | African | Lower middle income | Medium |
| Tanzania | Men | Later Adulthood | 1.91 | 7.8 | 5.88 | 2 | 5.23 (5.21 to 5.25) | African | Lower middle income | Medium |
| Tanzania | Women | Early Adulthood | 2.62 | 15.43 | 12.81 | 3 | 6.46 (6.44 to 6.47) | African | Lower middle income | Medium |
| Tanzania | Women | Later Adulthood | 4.31 | 19.06 | 14.75 | 3 | 5.37 (5.35 to 5.39) | African | Lower middle income | Medium |
| Thailand | Men | Early Adulthood | 1.59 | 12.83 | 11.25 | 2 | 7.81 (7.79 to 7.83) | South-East Asia | Upper middle income | High |
| Thailand | Men | Later Adulthood | 1.79 | 6.84 | 5.05 | 3 | 4.91 (4.90 to 4.93) | South-East Asia | Upper middle income | High |
| Thailand | Women | Early Adulthood | 5.76 | 19.03 | 13.27 | 2 | 4.41 (4.40 to 4.42) | South-East Asia | Upper middle income | High |
| Thailand | Women | Later Adulthood | 6.51 | 14.16 | 7.66 | 2 | 2.84 (2.82 to 2.85) | South-East Asia | Upper middle income | High |
| Timor-Leste | Men | Early Adulthood | 0.17 | 1.59 | 1.42 | 1 | 8.24 (8.18 to 8.29) | South-East Asia | Lower middle income | Medium |
| Timor-Leste | Men | Later Adulthood | 0.2 | 0.89 | 0.69 | 3 | 5.56 (5.53 to 5.60) | South-East Asia | Lower middle income | Medium |
| Timor-Leste | Women | Early Adulthood | 0.43 | 2.61 | 2.18 | 2 | 6.71 (6.69 to 6.73) | South-East Asia | Lower middle income | Medium |
| Timor-Leste | Women | Later Adulthood | 0.57 | 2.75 | 2.18 | 2 | 5.80 (5.79 to 5.82) | South-East Asia | Lower middle income | Medium |
| Togo | Men | Early Adulthood | 0.69 | 4.31 | 3.62 | 1 | 6.77 (6.74 to 6.81) | African | Low income | Medium |
| Togo | Men | Later Adulthood | 1.3 | 7 | 5.69 | 1 | 6.18 (6.14 to 6.22) | African | Low income | Medium |
| Togo | Women | Early Adulthood | 2.01 | 13.71 | 11.7 | 1 | 7.10 (7.08 to 7.13) | African | Low income | Medium |
| Togo | Women | Later Adulthood | 3.4 | 20.85 | 17.44 | 1 | 6.71 (6.68 to 6.74) | African | Low income | Medium |
| Tonga | Men | Early Adulthood | 36.96 | 62.49 | 25.53 | 3 | 1.90 (1.89 to 1.91) | Western Pacific | Upper middle income | High |
| Tonga | Men | Later Adulthood | 40.43 | 58.61 | 18.18 | 2 | 1.34 (1.33 to 1.34) | Western Pacific | Upper middle income | High |
| Tonga | Women | Early Adulthood | 55.58 | 79.39 | 23.81 | 2 | 1.28 (1.28 to 1.29) | Western Pacific | Upper middle income | High |
| Tonga | Women | Later Adulthood | 61.69 | 79.86 | 18.17 | 1 | 0.92 (0.92 to 0.93) | Western Pacific | Upper middle income | High |
| Trinidad and Tobago | Men | Early Adulthood | 10.76 | 22.9 | 12.14 | 3 | 2.80 (2.78 to 2.82) | Americas | High income | Very High |
| Trinidad and Tobago | Men | Later Adulthood | 11.89 | 22.56 | 10.67 | 2 | 2.33 (2.32 to 2.34) | Americas | High income | Very High |
| Trinidad and Tobago | Women | Early Adulthood | 19.2 | 33.74 | 14.54 | 1 | 2.08 (2.06 to 2.10) | Americas | High income | Very High |
| Trinidad and Tobago | Women | Later Adulthood | 25.03 | 36.96 | 11.93 | 3 | 1.40 (1.40 to 1.41) | Americas | High income | Very High |
| Tunisia | Men | Early Adulthood | 4.87 | 17.39 | 12.52 | 3 | 4.67 (4.65 to 4.69) | Eastern Mediterranean | Lower middle income | High |
| Tunisia | Men | Later Adulthood | 7.41 | 22.86 | 15.45 | 2 | 4.09 (4.07 to 4.11) | Eastern Mediterranean | Lower middle income | High |
| Tunisia | Women | Early Adulthood | 16.02 | 27.93 | 11.91 | 2 | 2.05 (2.04 to 2.06) | Eastern Mediterranean | Lower middle income | High |
| Tunisia | Women | Later Adulthood | 25.27 | 49.5 | 24.23 | 3 | 2.42 (2.41 to 2.42) | Eastern Mediterranean | Lower middle income | High |
| Turkiye | Men | Early Adulthood | 9.61 | 21.1 | 11.49 | 3 | 2.89 (2.88 to 2.90) | European | Upper middle income | Very High |
| Turkiye | Men | Later Adulthood | 15.63 | 35.23 | 19.59 | 3 | 2.98 (2.96 to 3.00) | European | Upper middle income | Very High |
| Turkiye | Women | Early Adulthood | 21.07 | 31.11 | 10.03 | 1 | 1.41 (1.39 to 1.42) | European | Upper middle income | Very High |
| Turkiye | Women | Later Adulthood | 38.05 | 64.4 | 26.35 | 3 | 1.90 (1.89 to 1.91) | European | Upper middle income | Very High |
| Turkmenistan | Men | Early Adulthood | 4.93 | 12.77 | 7.84 | 1 | 3.46 (3.45 to 3.48) | European | Upper middle income | High |
| Turkmenistan | Men | Later Adulthood | 10.95 | 28.18 | 17.23 | 3 | 3.39 (3.38 to 3.39) | European | Upper middle income | High |
| Turkmenistan | Women | Early Adulthood | 7.23 | 15.39 | 8.17 | 3 | 2.76 (2.74 to 2.77) | European | Upper middle income | High |
| Turkmenistan | Women | Later Adulthood | 16.67 | 39.7 | 23.03 | 2 | 3.12 (3.10 to 3.13) | European | Upper middle income | High |
| Tuvalu | Men | Early Adulthood | 42.35 | 57.57 | 15.22 | 2 | 1.16 (1.14 to 1.18) | Western Pacific | Upper middle income | Medium |
| Tuvalu | Men | Later Adulthood | 41.78 | 56.15 | 14.37 | 2 | 1.04 (1.03 to 1.05) | Western Pacific | Upper middle income | Medium |
| Tuvalu | Women | Early Adulthood | 55.23 | 70.76 | 15.53 | 3 | 0.96 (0.95 to 0.96) | Western Pacific | Upper middle income | Medium |
| Tuvalu | Women | Later Adulthood | 56.16 | 72.53 | 16.37 | 2 | 0.90 (0.89 to 0.91) | Western Pacific | Upper middle income | Medium |
| Uganda | Men | Early Adulthood | 0.25 | 2.92 | 2.68 | 2 | 9.31 (9.26 to 9.35) | African | Low income | Medium |
| Uganda | Men | Later Adulthood | 0.55 | 4.01 | 3.46 | 3 | 7.33 (7.30 to 7.36) | African | Low income | Medium |
| Uganda | Women | Early Adulthood | 1.53 | 9.18 | 7.65 | 1 | 6.63 (6.60 to 6.65) | African | Low income | Medium |
| Uganda | Women | Later Adulthood | 2.43 | 11.43 | 9 | 3 | 5.59 (5.57 to 5.60) | African | Low income | Medium |
| Ukraine | Men | Early Adulthood | 7.2 | 16.42 | 9.22 | 2 | 3.03 (2.99 to 3.06) | European | Upper middle income | High |
| Ukraine | Men | Later Adulthood | 15.08 | 29.9 | 14.82 | 3 | 2.43 (2.42 to 2.43) | European | Upper middle income | High |
| Ukraine | Women | Early Adulthood | 16.53 | 21.04 | 4.52 | 2 | 0.89 (0.87 to 0.92) | European | Upper middle income | High |
| Ukraine | Women | Later Adulthood | 36.68 | 43.59 | 6.91 | 1 | 0.62 (0.61 to 0.63) | European | Upper middle income | High |
| United Arab Emirates | Men | Early Adulthood | 16.22 | 28.28 | 12.06 | 3 | 2.05 (2.03 to 2.08) | Eastern Mediterranean | High income | Very High |
| United Arab Emirates | Men | Later Adulthood | 18.8 | 32.02 | 13.22 | 3 | 1.95 (1.92 to 1.98) | Eastern Mediterranean | High income | Very High |
| United Arab Emirates | Women | Early Adulthood | 28.97 | 32.72 | 3.75 | 3 | 0.45 (0.45 to 0.46) | Eastern Mediterranean | High income | Very High |
| United Arab Emirates | Women | Later Adulthood | 37.66 | 56.08 | 18.42 | 3 | 1.46 (1.44 to 1.47) | Eastern Mediterranean | High income | Very High |
| United Kingdom | Men | Early Adulthood | 10.75 | 23.21 | 12.46 | 2 | 2.84 (2.80 to 2.88) | European | High income | Very High |
| United Kingdom | Men | Later Adulthood | 14.87 | 33.45 | 18.58 | 2 | 2.98 (2.95 to 3.02) | European | High income | Very High |
| United Kingdom | Women | Early Adulthood | 12.53 | 25.73 | 13.2 | 3 | 2.67 (2.65 to 2.69) | European | High income | Very High |
| United Kingdom | Women | Later Adulthood | 19.74 | 32.49 | 12.75 | 3 | 1.82 (1.81 to 1.84) | European | High income | Very High |
| United States of America | Men | Early Adulthood | 17.38 | 38.8 | 21.42 | 2 | 2.94 (2.91 to 2.96) | Americas | High income | Very High |
| United States of America | Men | Later Adulthood | 20.65 | 44.7 | 24.04 | 2 | 2.80 (2.79 to 2.82) | Americas | High income | Very High |
| United States of America | Women | Early Adulthood | 20.94 | 41.31 | 20.37 | 3 | 2.50 (2.49 to 2.52) | Americas | High income | Very High |
| United States of America | Women | Later Adulthood | 25.38 | 45.15 | 19.76 | 3 | 2.08 (2.07 to 2.09) | Americas | High income | Very High |
| Uruguay | Men | Early Adulthood | 9.29 | 27.72 | 18.43 | 3 | 4.01 (4.00 to 4.02) | Americas | High income | Very High |
| Uruguay | Men | Later Adulthood | 15.16 | 33.74 | 18.57 | 1 | 2.93 (2.92 to 2.93) | Americas | High income | Very High |
| Uruguay | Women | Early Adulthood | 10.98 | 30.32 | 19.34 | 3 | 3.70 (3.69 to 3.71) | Americas | High income | Very High |
| Uruguay | Women | Later Adulthood | 25.87 | 44.27 | 18.39 | 1 | 1.96 (1.95 to 1.96) | Americas | High income | Very High |
| Uzbekistan | Men | Early Adulthood | 4.32 | 19.32 | 14.99 | 1 | 5.43 (5.41 to 5.45) | European | Lower middle income | High |
| Uzbekistan | Men | Later Adulthood | 10.3 | 37.19 | 26.89 | 2 | 4.59 (4.56 to 4.61) | European | Lower middle income | High |
| Uzbekistan | Women | Early Adulthood | 6.15 | 21.24 | 15.09 | 2 | 4.53 (4.51 to 4.55) | European | Lower middle income | High |
| Uzbekistan | Women | Later Adulthood | 18.03 | 53.64 | 35.61 | 3 | 3.90 (3.88 to 3.91) | European | Lower middle income | High |
| Vanuatu | Men | Early Adulthood | 11.2 | 14.05 | 2.85 | 2 | 0.88 (0.86 to 0.91) | Western Pacific | Lower middle income | Medium |
| Vanuatu | Men | Later Adulthood | 12.82 | 20.34 | 7.52 | 3 | 1.74 (1.73 to 1.76) | Western Pacific | Lower middle income | Medium |
| Vanuatu | Women | Early Adulthood | 16.11 | 23.44 | 7.34 | 2 | 1.44 (1.43 to 1.45) | Western Pacific | Lower middle income | Medium |
| Vanuatu | Women | Later Adulthood | 17.2 | 30.22 | 13.03 | 3 | 2.07 (2.06 to 2.08) | Western Pacific | Lower middle income | Medium |
| Venezuela | Men | Early Adulthood | 12.58 | 22.11 | 9.53 | 3 | 2.08 (2.06 to 2.11) | Americas | Unclassified | High |
| Venezuela | Men | Later Adulthood | 13.54 | 19.45 | 5.9 | 3 | 1.31 (1.28 to 1.34) | Americas | Unclassified | High |
| Venezuela | Women | Early Adulthood | 13.51 | 24.64 | 11.13 | 3 | 2.23 (2.20 to 2.25) | Americas | Unclassified | High |
| Venezuela | Women | Later Adulthood | 20.67 | 28.74 | 8.08 | 3 | 1.19 (1.18 to 1.21) | Americas | Unclassified | High |
| Viet Nam | Men | Early Adulthood | 0.08 | 1.78 | 1.69 | 2 | 11.32 (11.27 to 11.38) | Western Pacific | Lower middle income | High |
| Viet Nam | Men | Later Adulthood | 0.1 | 1.49 | 1.39 | 3 | 9.86 (9.80 to 9.91) | Western Pacific | Lower middle income | High |
| Viet Nam | Women | Early Adulthood | 0.32 | 1.25 | 0.92 | 3 | 4.82 (4.78 to 4.86) | Western Pacific | Lower middle income | High |
| Viet Nam | Women | Later Adulthood | 0.32 | 3.1 | 2.79 | 3 | 8.27 (8.24 to 8.30) | Western Pacific | Lower middle income | High |
| Yemen | Men | Early Adulthood | 2.26 | 8 | 5.75 | 1 | 4.60 (4.58 to 4.62) | Eastern Mediterranean | Low income | Low |
| Yemen | Men | Later Adulthood | 3.26 | 12.1 | 8.84 | 2 | 4.78 (4.78 to 4.78) | Eastern Mediterranean | Low income | Low |
| Yemen | Women | Early Adulthood | 4.02 | 12.13 | 8.11 | 3 | 3.97 (3.95 to 3.98) | Eastern Mediterranean | Low income | Low |
| Yemen | Women | Later Adulthood | 5.68 | 22.74 | 17.07 | 1 | 5.07 (5.04 to 5.09) | Eastern Mediterranean | Low income | Low |
| Zambia | Men | Early Adulthood | 0.57 | 3.42 | 2.85 | 3 | 6.59 (6.58 to 6.61) | African | Lower middle income | Medium |
| Zambia | Men | Later Adulthood | 1.32 | 6.75 | 5.43 | 3 | 6.02 (6.01 to 6.04) | African | Lower middle income | Medium |
| Zambia | Women | Early Adulthood | 2.38 | 12.74 | 10.36 | 3 | 6.09 (6.08 to 6.10) | African | Lower middle income | Medium |
| Zambia | Women | Later Adulthood | 5.56 | 19.77 | 14.21 | 3 | 4.56 (4.55 to 4.58) | African | Lower middle income | Medium |
| Zimbabwe | Men | Early Adulthood | 0.91 | 3.76 | 2.85 | 1 | 5.17 (5.08 to 5.27) | African | Lower middle income | Medium |
| Zimbabwe | Men | Later Adulthood | 1.62 | 7 | 5.38 | 3 | 5.38 (5.37 to 5.40) | African | Lower middle income | Medium |
| Zimbabwe | Women | Early Adulthood | 7.78 | 18.19 | 10.41 | 2 | 3.05 (2.99 to 3.10) | African | Lower middle income | Medium |
| Zimbabwe | Women | Later Adulthood | 13.41 | 24.07 | 10.65 | 2 | 2.07 (2.05 to 2.08) | African | Lower middle income | Medium |

AAPC: average annual percent change; CI: confidence interval; HDI: Human Development Index. World Bank income was categorized using 2022 fiscal year data, with Venezuela unclassified and excluded from the analysis.

**Supplementary Table S2. Median obesity prevalence at baseline (1990 to 1994) and endline (2018 to 2022), prevalence change, and average annual percent change (AAPC) by Human Development Index (HDI)**

| **HDI** | **N** | **Women, age 20-49** | **Women, age ≥50** | **Men, age 20-49** | **Men, age ≥50** |
| --- | --- | --- | --- | --- | --- |
|  |  | **Baseline Prevalence (%) 1990-1994 (Median (IQR))** | | | |
| Low | 26 | 2.1 (1.4 to 3.0) | 3.3 (2.0 to 5.0) | 0.8 (0.5 to 1.0) | 1.6 (0.9 to 1.8) |
| Medium | 42 | 4.4 (2.4 to 11.8) | 6.7 (3.8 to 15.7) | 1.3 (0.9 to 5.1) | 2.4 (1.5 to 8.9) |
| High | 50 | 12.8 (8.2 to 18.2) | 20.4 (15.5 to 28.0) | 5.3 (4.2 to 10.3) | 8.2 (5.1 to 14.5) |
| Very High | 77 | 11.7 (8.8 to 17.0) | 25.4 (20.2 to 30.6) | 9.6 (7.1 to 11.9) | 15.9 (11.4 to 18.1) |
|  |  | **Endline Prevalence (%) 2018-2022 (Median (IQR))** | | | |
| Low | 26 | 10.4 (7.3 to 13.7) | 14.4 (9.5 to 21.8) | 4.2 (3.1 to 7.0) | 7.4 (4.8 to 10.3) |
| Medium | 42 | 18.1 (12.8 to 28.0) | 23.9 (15.7 to 35.9) | 6.4 (4.2 to 14.4) | 10.1 (6.7 to 19.8) |
| High | 50 | 28.5 (21.9 to 39.6) | 43.6 (35.9 to 49.3) | 18.2 (13.2 to 24.6) | 22.9 (17.0 to 33.0) |
| Very High | 77 | 20.0 (15.0 to 33.7) | 38.1 (28.5 to 45.1) | 22.7 (17.3 to 28.3) | 30.8 (24.9 to 37.4) |
|  |  | **Prevalence Change (%) from 1990-1994 to 2018-2022 (Median (IQR))** | | | |
| Low | 26 | 8.1 (6.0 to 10.6) | 11.8 (7.8 to 16.5) | 3.4 (2.7 to 5.5) | 5.7 (4.0 to 8.5) |
| Medium | 42 | 11.0 (8.1 to 15.6) | 15.2 (10.2 to 18.6) | 4.7 (3.3 to 6.5) | 7.3 (5.0 to 9.8) |
| High | 50 | 15.1 (11.5 to 21.5) | 19.4 (14.8 to 23.2) | 12.4 (8.9 to 15.0) | 13.5 (10.7 to 17.8) |
| Very High | 77 | 8.2 (4.6 to 14.9) | 9.0 (2.4 to 19.7) | 12.5 (9.8 to 16.6) | 15.5 (11.3 to 19.0) |
|  |  | **AAPC 1990-2022 (Median (IQR))** | | | |
| Low | 26 | 5.9 (5.5 to 6.8) | 5.4 (4.8 to 6.4) | 6.3 (6.1 to 7.1) | 5.9 (5.5 to 6.3) |
| Medium | 42 | 4.6 (3.3 to 6.4) | 4.0 (2.8 to 5.3) | 5.6 (4.0 to 6.6) | 5.2 (3.7 to 5.8) |
| High | 50 | 3.3 (2.2 to 4.1) | 2.5 (1.9 to 3.1) | 4.5 (3.0 to 5.2) | 3.9 (2.9 to 4.6) |
| Very High | 77 | 2.0 (1.3 to 2.9) | 1.1 (0.4 to 2.1) | 3.0 (2.5 to 3.9) | 2.7 (2.0 to 3.4) |

N: number of countries and territories in each category. Interquartile range (IQR) consists of quartile 1 to quartile 3 values. HDI categories are based on the United Nations Development Programme classifications, with North Korea unclassified and excluded from the analysis.

**Supplementary Table S3. Prevalence of body mass index (BMI) ≥25 kg/m² and ≥30 kg/m² in South-East Asian and Western Pacific regions**

| **Obesity Definition** | WHO Region | **N** | **Women, age 20-49** | **Women, age ≥50** | **Men, age 20-49** | **Men, age ≥50** |
| --- | --- | --- | --- | --- | --- | --- |
|  |  |  | **Baseline Prevalence (%) in 1990-1994 (Median (IQR))** | | | |
| BMI ≥25 kg/m² | South-East Asia | 11 | 14.5 (5.6 to 14.9) | 13.6 (7.7 to 17.7) | 7.9 (6.3 to 11.0) | 10.0 (7.2 to 14.6) |
|  | Western Pacific | 28 | 23.8 (14.8 to 39.2) | 35.5 (19.2 to 43.4) | 23.1 (11.2 to 27.7) | 26.0 (15.1 to 32.8) |
| BMI ≥30 kg/m² | South-East Asia | 11 | 2.3 (0.8 to 3.0) | 2.8 (1.4 to 3.7) | 0.7 (0.5 to 1.2) | 0.8 (0.7 to 1.7) |
|  | Western Pacific | 28 | 14.5 (3.4 to 39.2) | 17.6 (5.7 to 39.4) | 11.0 (2.1 to 27.7) | 12.5 (2.2 to 30.1) |
| **Endline Prevalence (%) in 2018-2022 (Median (IQR))** | | | | | | |
| BMI ≥25 kg/m² | South-East Asia | 11 | 39.3 (31.3 to 49.1) | 41.8 (33.3 to 47.7) | 28.8 (22.3 to 34.5) | 26.8 (24.7 to 35.0) |
|  | Western Pacific | 28 | 37.4 (26.5 to 55.8) | 44.1 (36.2 to 63.8) | 39.8 (26.7 to 49.3) | 38.6 (32.3 to 53.3) |
| BMI ≥30 kg/m² | South-East Asia | 11 | 12.5 (7.6 to 15.3) | 12.2 (9.7 to 13.4) | 6.1 (4.4 to 8.0) | 5.8 (4.4 to 6.8) |
|  | Western Pacific | 28 | 26.6 (9.5 to 54.0) | 33.2 (10.9 to 56.2) | 20.9 (12.4 to 39.0) | 26.3 (6.6 to 38.4) |
| **Prevalence Change (%) from 1990-1994 to 2018-2022 (Median (IQR))** | | | | | | |
| BMI ≥25 kg/m² | South-East Asia | 11 | 27.5 (23.3 to 32.1) | 23.8 (21.3 to 30.4) | 21.6 (15.8 to 24.6) | 19.5 (15.1 to 20.5) |
|  | Western Pacific | 28 | 15.0 (9.9 to 19.5) | 14.9 (8.3 to 20.1) | 14.6 (11.1 to 23.5) | 15.3 (9.3 to 20.1) |
| BMI ≥30 kg/m² | South-East Asia | 11 | 9.5 (6.6 to 12.6) | 8.3 (7.4 to 10.1) | 5.5 (3.7 to 6.9) | 4.9 (3.7 to 5.2) |
|  | Western Pacific | 28 | 10.8 (6.2 to 15.2) | 11.5 (4.8 to 15.9) | 11.0 (6.2 to 14.2) | 8.2 (4.6 to 14.3) |
| **AAPC 1990-2022 (Median (IQR))** | | | | | | |
| BMI ≥25 kg/m² | South-East Asia | 11 | 4.5 (3.8 to 5.1) | 4.0 (3.0 to 4.5) | 4.4 (4.0 to 5.1) | 3.5 (3.3 to 4.2) |
|  | Western Pacific | 28 | 1.7 (0.9 to 2.9) | 1.5 (0.7 to 2.3) | 2.3 (1.2 to 3.0) | 1.7 (1.2 to 2.7) |
| BMI ≥30 kg/m² | South-East Asia | 11 | 6.8 (5.4 to 7.3) | 5.7 (4.1 to 6.6) | 7.9 (7.4 to 8.4) | 5.9 (5.5 to 6.9) |
|  | Western Pacific | 28 | 1.9 (1.3 to 3.9) | 1.8 (0.9 to 2.9) | 2.8 (1.2 to 6.5) | 2.5 (1.3 to 4.7) |

N: number of countries/territories. AAPC: average annual percent change. Interquartile range (IQR) consists of quartile 1 to quartile 3 values. The BMI ≥25 kg/m² prevalence was calculated for all 11 countries in the South-East Asian region. In the Western Pacific region, the BMI ≥25 kg/m² prevalence was not calculated for the following 16 countries and territories, which were instead assessed using BMI ≥30 kg/m²: American Samoa, Australia, Federated States of Micronesia, Fiji, French Polynesia, Kiribati, Marshall Islands, Nauru, New Zealand, Palau, Papua New Guinea, Samoa, Solomon Islands, Tonga, Tuvalu, and Vanuatu.

**Supplementary Table S4. Median obesity prevalence at baseline (1990 to 1994) and endline (2018 to 2022), prevalence change, and average annual percent change (AAPC) by 10-year age groups**

|  | **Category** | **N** | **20-29 years** | **30-39 years** | **40-49 years** | **50-59 years** | **60-69 years** | **≥70 years** |
| --- | --- | --- | --- | --- | --- | --- | --- | --- |
|  |  |  | **Baseline Prevalence (%) 1990-1994 (Median (IQR))** | | | | | |
|  | Global | 196 | 4.2 (1.4 to 7.2) | 8.9 (3.2 to 13.1) | 13.7 (4.9 to 18.3) | 16.3 (5.3 to 22.8) | 14.9 (4.0 to 22.6) | 10.4 (2.3 to 17.5) |
| WHO Region | African | 47 | 0.9 (0.5 to 2.0) | 2.3 (1.5 to 4.7) | 3.7 (2.4 to 6.8) | 4.2 (2.7 to 7.3) | 3.1 (2.0 to 5.7) | 1.7 (1.1 to 3.6) |
|  | Americas | 37 | 6.6 (5.0 to 8.4) | 12.6 (9.9 to 14.3) | 16.7 (13.7 to 19.0) | 18.6 (15.2 to 21.9) | 15.8 (13.8 to 21.1) | 10.7 (9.3 to 14.6) |
|  | Eastern Mediterranean | 21 | 9.0 (1.8 to 11.6) | 16.0 (4.3 to 21.0) | 21.1 (6.2 to 25.5) | 24.1 (7.2 to 27.5) | 19.5 (6.3 to 23.6) | 10.9 (4.4 to 15.4) |
|  | European | 52 | 4.7 (3.6 to 5.4) | 9.7 (8.1 to 11.5) | 15.8 (12.6 to 17.6) | 20.7 (17.6 to 24.3) | 23.2 (19.6 to 27.8) | 19.6 (16.1 to 23.4) |
|  | South-East Asia | 11 | 0.8 (0.2 to 1.1) | 1.9 (0.7 to 2.4) | 2.5 (1.2 to 3.2) | 2.3 (1.3 to 3.3) | 1.5 (1.0 to 2.6) | 0.7 (0.6 to 1.5) |
|  | Western Pacific | 28 | 8.0 (1.9 to 24.4) | 14.6 (2.9 to 39.8) | 17.6 (3.6 to 45.1) | 19.5 (4.1 to 42.5) | 13.8 (4.0 to 34.2) | 8.1 (3.0 to 23.4) |
| World Bank Income Level | Low income | 26 | 0.6 (0.4 to 0.9) | 1.7 (1.1 to 2.3) | 2.7 (1.7 to 3.4) | 2.9 (1.7 to 3.5) | 2.1 (1.3 to 2.7) | 1.2 (0.7 to 1.6) |
|  | Lower middle income | 50 | 1.5 (0.8 to 4.3) | 3.7 (2.3 to 8.9) | 5.4 (3.4 to 12.9) | 5.7 (3.8 to 14.8) | 4.4 (2.9 to 12.8) | 2.6 (1.6 to 7.6) |
|  | Upper middle income | 53 | 5.1 (3.2 to 7.4) | 11.1 (7.7 to 13.2) | 15.9 (11.9 to 19.6) | 18.7 (13.8 to 24.4) | 16.9 (12.2 to 23.3) | 11.0 (7.5 to 19.3) |
|  | High income | 66 | 5.6 (4.2 to 9.6) | 11.3 (8.7 to 15.6) | 17.2 (13.9 to 21.5) | 21.6 (18.3 to 25.7) | 22.1 (18.3 to 26.4) | 16.8 (13.2 to 21.6) |
| Baseline (1990-1994) prevalence level | Low | 66 | 0.9 (0.5 to 1.4) | 2.2 (1.2 to 3.2) | 3.1 (1.9 to 4.9) | 3.2 (2.1 to 5.3) | 2.6 (1.6 to 4.0) | 1.6 (0.9 to 2.3) |
|  | Medium | 65 | 4.2 (3.4 to 5.0) | 8.9 (7.7 to 10.3) | 13.7 (11.7 to 15.8) | 16.3 (13.8 to 19.2) | 15.0 (12.5 to 18.3) | 10.5 (7.7 to 12.9) |
|  | High | 65 | 8.9 (7.2 to 13.8) | 16.0 (13.1 to 21.6) | 21.3 (18.4 to 27.1) | 25.7 (22.8 to 31.3) | 25.5 (22.8 to 32.3) | 20.0 (17.5 to 25.1) |
| **Endline Prevalence (%) 2018-2022 (Median (IQR))** | | | | | | | | |
| Global | | 196 | 11.5 (6.7 to 19.4) | 19.1 (11.9 to 29.5) | 26.8 (15.6 to 35.4) | 30.8 (16.4 to 38.8) | 30.0 (15.5 to 40.6) | 23.9 (10.9 to 34.4) |
| WHO Region | African | 47 | 5.4 (3.8 to 8.5) | 11.4 (8.2 to 16.1) | 14.6 (10.1 to 20.2) | 15.2 (10.7 to 20.7) | 14.1 (9.5 to 19.3) | 10.2 (6.4 to 15.0) |
|  | Americas | 37 | 20.5 (18.3 to 25.3) | 32.0 (28.7 to 35.7) | 38.1 (33.7 to 41.2) | 37.1 (33.7 to 42.5) | 32.6 (31.4 to 39.7) | 25.5 (22.8 to 28.9) |
|  | Eastern Mediterranean | 21 | 17.6 (12.0 to 23.9) | 28.8 (19.4 to 34.9) | 35.4 (26.9 to 46.7) | 40.7 (26.3 to 49.6) | 41.0 (22.3 to 49.6) | 35.3 (16.2 to 44.4) |
|  | European | 52 | 10.1 (7.9 to 13.2) | 17.8 (15.4 to 21.3) | 26.0 (21.5 to 30.3) | 33.6 (26.8 to 38.0) | 37.1 (29.3 to 42.8) | 34.4 (27.4 to 39.9) |
|  | South-East Asia | 11 | 6.8 (3.6 to 8.3) | 11.1 (7.1 to 12.3) | 12.2 (8.8 to 14.0) | 11.4 (8.5 to 12.5) | 8.6 (7.0 to 10.2) | 5.8 (3.9 to 6.5) |
|  | Western Pacific | 28 | 19.9 (9.6 to 35.4) | 26.1 (11.4 to 52.2) | 31.1 (10.8 to 56.1) | 32.2 (9.6 to 52.6) | 30.6 (8.7 to 44.7) | 25.1 (7.4 to 33.3) |
| World Bank Income Level | Low income | 26 | 4.0 (2.7 to 6.5) | 8.9 (6.2 to 11.5) | 11.4 (8.4 to 16.4) | 11.1 (8.5 to 18.2) | 9.7 (7.0 to 15.8) | 6.6 (4.7 to 10.8) |
|  | Lower middle income | 50 | 6.8 (4.9 to 12.9) | 14.3 (9.9 to 22.6) | 19.0 (12.6 to 29.0) | 19.5 (13.8 to 34.0) | 17.0 (11.5 to 30.7) | 11.5 (8.2 to 22.2) |
|  | Upper middle income | 53 | 15.4 (10.2 to 21.0) | 26.8 (17.5 to 32.8) | 31.7 (25.4 to 38.6) | 35.1 (31.8 to 40.9) | 34.2 (29.7 to 42.4) | 26.9 (22.4 to 37.5) |
|  | High income | 66 | 14.9 (10.2 to 24.6) | 24.8 (16.7 to 34.5) | 30.3 (22.4 to 38.4) | 36.1 (26.7 to 42.6) | 36.2 (28.4 to 43.3) | 31.3 (24.7 to 38.0) |
| Baseline (1990-1994) prevalence level | Low | 66 | 5.4 (3.6 to 7.2) | 10.5 (7.1 to 12.8) | 12.7 (8.8 to 16.6) | 13.3 (9.1 to 17.4) | 10.8 (7.6 to 15.5) | 8.1 (5.0 to 11.3) |
|  | Medium | 65 | 10.7 (8.2 to 13.8) | 19.1 (16.5 to 23.5) | 28.4 (22.6 to 33.7) | 33.1 (27.8 to 37.2) | 31.7 (26.9 to 36.6) | 24.4 (19.9 to 30.6) |
|  | High | 65 | 23.4 (18.8 to 29.8) | 34.0 (27.7 to 41.2) | 39.2 (30.5 to 47.3) | 42.5 (36.2 to 49.6) | 42.7 (38.7 to 47.7) | 37.9 (31.8 to 44.0) |
| **Prevalence Change (%) from 1990-1994 to 2018-2022 (Median (IQR))** | | | | | | | | |
| Global | | 196 | 7.2 (4.1 to 11.8) | 10.7 (7.2 to 15.3) | 11.9 (7.7 to 17.7) | 12.3 (7.3 to 18.3) | 12.6 (7.1 to 18.5) | 11.3 (6.3 to 16.4) |
| WHO Region | African | 47 | 4.3 (2.9 to 6.3) | 8.5 (6.1 to 11.3) | 11.0 (7.5 to 13.6) | 10.9 (7.2 to 13.9) | 10.7 (6.7 to 12.8) | 8.3 (5.1 to 10.2) |
|  | Americas | 37 | 14.2 (13.3 to 18.3) | 20.2 (19.0 to 22.0) | 21.3 (19.5 to 22.3) | 20.0 (18.0 to 21.1) | 18.4 (16.3 to 19.5) | 15.1 (13.3 to 16.5) |
|  | Eastern Mediterranean | 21 | 8.5 (6.8 to 12.3) | 12.1 (10.7 to 15.5) | 15.1 (13.5 to 18.0) | 17.8 (15.5 to 20.6) | 21.2 (16.7 to 24.2) | 24.4 (14.1 to 27.4) |
|  | European | 52 | 5.7 (3.8 to 9.0) | 7.8 (6.2 to 11.0) | 9.3 (6.1 to 13.4) | 9.9 (5.7 to 15.2) | 10.7 (6.0 to 16.7) | 11.6 (6.4 to 18.6) |
|  | South-East Asia | 11 | 6.0 (3.3 to 7.2) | 8.9 (5.7 to 10.4) | 8.9 (6.9 to 10.6) | 8.1 (6.8 to 9.4) | 6.4 (5.9 to 7.5) | 4.5 (3.3 to 5.5) |
|  | Western Pacific | 28 | 9.5 (5.0 to 14.7) | 9.8 (6.9 to 14.0) | 9.0 (6.4 to 14.4) | 9.4 (5.3 to 14.2) | 10.5 (5.2 to 16.1) | 9.7 (4.1 to 16.0) |
| World Bank Income Level | Low income | 26 | 3.5 (2.3 to 5.3) | 7.1 (5.0 to 9.1) | 8.4 (6.3 to 11.6) | 8.3 (6.5 to 13.1) | 7.5 (5.9 to 12.2) | 5.1 (4.0 to 9.2) |
|  | Lower middle income | 50 | 5.1 (3.6 to 7.6) | 9.5 (7.4 to 11.8) | 11.8 (8.8 to 14.7) | 11.9 (9.2 to 16.8) | 11.0 (8.3 to 18.6) | 8.6 (5.8 to 14.5) |
|  | Upper middle income | 53 | 10.5 (6.9 to 14.0) | 14.0 (10.6 to 19.4) | 16.1 (11.8 to 19.6) | 17.2 (12.1 to 18.7) | 16.7 (12.8 to 19.5) | 14.6 (11.3 to 17.7) |
|  | High income | 66 | 9.2 (5.8 to 13.5) | 10.5 (6.7 to 15.4) | 10.4 (6.4 to 17.9) | 10.4 (5.6 to 19.0) | 11.2 (5.8 to 19.0) | 12.2 (6.2 to 17.7) |
| Baseline (1990-1994) prevalence level | Low | 66 | 4.2 (3.1 to 6.2) | 7.7 (5.7 to 10.1) | 9.7 (6.8 to 11.8) | 9.4 (6.5 to 12.3) | 8.0 (5.7 to 11.6) | 6.2 (4.1 to 8.7) |
|  | Medium | 65 | 6.5 (4.4 to 10.1) | 10.6 (7.3 to 15.7) | 14.5 (9.4 to 19.5) | 17.4 (10.1 to 19.4) | 17.3 (12.4 to 19.5) | 14.6 (11.3 to 17.1) |
|  | High | 65 | 12.3 (9.2 to 16.5) | 14.0 (10.9 to 20.6) | 13.9 (9.3 to 20.4) | 13.6 (8.7 to 20.3) | 15.1 (8.4 to 20.3) | 15.1 (9.8 to 21.6) |
| **AAPC 1990-2022 (Median (IQR))** | | | | | | | | |
| Global | | 196 | 4.1 (2.9 to 6.2) | 3.3 (2.1 to 5.1) | 2.8 (1.8 to 4.4) | 2.5 (1.5 to 4.3) | 2.8 (1.7 to 4.7) | 3.4 (2.1 to 5.5) |
| WHO Region | African | 47 | 6.6 (5.0 to 7.5) | 5.6 (4.4 to 6.5) | 5.0 (3.8 to 5.8) | 4.7 (3.6 to 5.6) | 5.3 (3.9 to 6.1) | 5.9 (4.5 to 6.7) |
|  | Americas | 37 | 4.2 (3.8 to 4.8) | 3.6 (3.2 to 4.0) | 3.0 (2.6 to 3.4) | 2.6 (2.4 to 3.0) | 2.6 (2.4 to 3.2) | 3.2 (2.8 to 3.5) |
|  | Eastern Mediterranean | 21 | 2.9 (2.4 to 4.8) | 2.3 (2.0 to 4.0) | 2.1 (1.8 to 3.8) | 2.3 (1.9 to 4.0) | 3.0 (2.7 to 4.2) | 4.2 (3.7 to 5.4) |
|  | European | 52 | 3.0 (2.3 to 3.6) | 2.3 (1.7 to 2.7) | 1.7 (1.2 to 2.2) | 1.4 (0.8 to 2.0) | 1.4 (0.8 to 2.2) | 1.5 (1.0 to 2.5) |
|  | South-East Asia | 11 | 8.2 (7.1 to 8.8) | 6.9 (5.4 to 7.5) | 6.1 (4.5 to 6.7) | 5.8 (4.3 to 6.6) | 6.1 (4.7 to 7.2) | 6.5 (5.6 to 7.9) |
|  | Western Pacific | 28 | 3.1 (1.4 to 6.1) | 2.4 (1.0 to 4.9) | 2.0 (0.9 to 4.0) | 2.0 (0.9 to 3.4) | 2.0 (1.2 to 3.3) | 2.8 (1.6 to 4.3) |
| World Bank Income Level | Low income | 26 | 7.2 (6.6 to 8.0) | 6.4 (5.7 to 6.9) | 5.8 (5.0 to 6.2) | 5.6 (4.6 to 6.0) | 6.0 (5.2 to 6.4) | 6.6 (5.9 to 7.2) |
|  | Lower middle income | 50 | 5.6 (3.4 to 6.9) | 4.8 (3.3 to 5.9) | 4.3 (3.0 to 5.2) | 4.2 (3.0 to 5.0) | 4.8 (3.2 to 5.4) | 5.5 (3.9 to 6.3) |
|  | Upper middle income | 53 | 4.0 (2.6 to 4.8) | 3.3 (2.3 to 3.8) | 2.7 (1.9 to 3.4) | 2.5 (1.7 to 3.0) | 2.7 (1.9 to 3.2) | 3.2 (2.3 to 3.8) |
|  | High income | 66 | 3.3 (2.6 to 3.9) | 2.5 (1.7 to 2.9) | 1.9 (1.3 to 2.5) | 1.6 (0.9 to 2.3) | 1.6 (0.8 to 2.4) | 2.0 (1.2 to 3.0) |
| Baseline (1990-1994) prevalence level | Low | 66 | 7.0 (6.1 to 8.0) | 5.9 (5.1 to 6.9) | 5.2 (4.4 to 6.1) | 4.8 (4.3 to 6.0) | 5.4 (4.7 to 6.3) | 6.1 (5.4 to 7.1) |
|  | Medium | 65 | 3.4 (2.7 to 4.5) | 2.8 (2.1 to 3.7) | 2.7 (1.8 to 3.3) | 2.5 (1.7 to 3.1) | 2.8 (2.1 to 3.3) | 3.3 (2.6 to 4.1) |
|  | High | 65 | 2.9 (2.2 to 3.9) | 2.2 (1.4 to 3.0) | 1.8 (1.1 to 2.3) | 1.5 (0.9 to 2.1) | 1.5 (0.9 to 2.3) | 1.8 (1.2 to 2.7) |

N: number of countries/territories. Interquartile range (IQR) consists of quartile 1 to quartile 3 values. Prevalence estimates are 5-year averages for both sexes combined. The ranges for low (bottom 33%), middle (middle 33%), and high (top 33%) tertiles of averaged 1990-1994 baseline prevalence are: for age 20-29 (<2.42%, 2.42-5.61%, >5.61%), age 30-39 (<6.13%, 6.13-11.78%, >11.78%), age 40-49 (<9.18%, 9.18-16.91%, >16.91%), age 50-59 (<10.33%, 10.33-20.51%, >20.51%), age 60-69 (<8.58%, 8.58-21.10%, >21.10%), and age ≥70 (<5.37%, 5.37-15.17%, >15.17%). World Bank income was categorized using 2022 fiscal year data, with Venezuela unclassified and excluded from the analysis.

**Supplementary Figure S1. Global obesity prevalence in early adulthood and later adulthood at 1990-1994 and 2018-2022 and the change in prevalence magnitude from 1990-1994 to 2018-2022, for women and men**

**(a)** Global prevalence of early adulthood (age 20-49 years) and later adulthood (age ≥50 years) obesity in 1990-1994 and 2018-2022 in women. **(b)** Change in early adulthood and later adulthood obesity prevalence magnitude from 1990-1994 to 2018-2022 in women. **(c)** Global prevalence of early adulthood and later adulthood obesity in 1990-1994 and 2018-2022 in men. **(d)** Change in early adulthood and later adulthood obesity prevalence magnitude from 1990-1994 to 2018-2022 in men.

Prevalence change is calculated as the difference between the mean prevalence in 2018-2022 and 1990-1994.

**Supplementary Figure S2. Quartiles of average annual percent change (AAPC) for obesity prevalence in early adulthood and later adulthood from 1990 to 2022 among women and men**

**(a)** AAPC by quartiles for early adulthood (age 20-49 years) and later adulthood (age ≥50 years) obesity in women. **(b)** AAPC by quartiles for early adulthood and later adulthood obesity in men. Q4 (red) represents the quartile with fastest increasing prevalence (highest AAPC values), whereas Q1 (blue) represents the quartile with the slowest increasing prevalence (lowest AAPC values).

**Supplementary Figure S3. Comparing changes in the magnitude and rate (average annual percent change (AAPC) of early adulthood and later adulthood obesity prevalence over time across 196 countries and territories for women and men**

**(a)** Change in prevalence magnitude from 1990–1994 to 2018–2022 for early adulthood (age 20-49 years) and later adulthood (age ≥50 years) obesity in women. **(b)** AAPC from 1990–2022 for early adulthood and later adulthood obesity in women. **(c)** Change in prevalence magnitude from 1990–1994 to 2018–2022 for early adulthood and later adulthood obesity in men. **(d)** AAPC from 1990–2022 for early adulthood and later adulthood obesity in men.

N: number of countries/territories. The dashed diagonal line represents equal change in prevalence over time for early adulthood and later adulthood obesity. Points below the diagonal represent countries/territories where the increase in magnitude or rate over time was greater for obesity prevalence in early adulthood than in later adulthood, whereas points above the diagonal indicate the opposite.
